# Multiple strategy enhanced hybrid algorithm BAGWO combining beetle antennae search and grey wolf optimizer for global optimization

**DOI:** 10.1038/s41598-025-98816-0

**Published:** 2025-05-02

**Authors:** Fan Zhang, Chuankai Liu, Peng Liu, Shuiting Ding, Tian Qiu, Jiajun Wang, Huipeng Du

**Affiliations:** 1 Research Institute of Aero-Engine, Beihang University, 37 Xueyuan Road, Haidian District, Beijing, 100191 China; 2 Aircraft/Engine Integrated System Safety Beijing Key Laboratory, Beihang University, 37 Xueyuan Road, Haidian District, Beijing, 100191 China

**Keywords:** Hybrid algorithm, Grey Wolf Optimizer, Beetle Antennae Search algorithm, Ablation experiments, Global optimization, BAGWO, Computational science, Applied mathematics

## Abstract

This study proposes BAGWO, a novel hybrid optimization algorithm that integrates the Beetle Antennae Search algorithm (BAS) and the Grey Wolf Optimizer (GWO) to leverage their complementary strengths while enhancing their original strategies. BAGWO introduces three key improvements: the charisma concept and its update strategy based on the sigmoid function, the local exploitation frequency update strategy driven by the cosine function, and the switching strategy for the antennae length decay rate. These improvements are rigorously validated through ablation experiments. Comprehensive evaluations on 24 benchmark functions from CEC 2005 and CEC 2017, along with eight real-world engineering problems, demonstrate that BAGWO achieves stable convergence and superior optimization performance. Extensive testing and quantitative statistical analyses confirm that BAGWO significantly outperforms competing algorithms in terms of solution accuracy and stability, highlighting its strong competitiveness and potential for practical applications in global optimization.

**Supplementary Information:**

The online version contains supplementary material available at 10.1038/s41598-025-98816-0.

## Introduction

Optimization is the act of finding the best solution from a decision space given certain constraints and objectives (single or multi-objective)^1^. In real-world production practices, it is often faced with numerous optimization problems, such as minimizing cost, risk and time and maximizing efficiency, profit and quality^2^,These optimization problems are prevalent in agricultural production, mechanical design and machining, production scheduling, path planning, aviation and aerospace, water conservancy infrastructure and various other aspects of production and daily life, significantly impacting our lives.

Optimization problems can be categorized into single-objective optimization and multi-objective optimization based on the number of optimization objectives. Multi-objective optimization is often more complex than single-objective optimization, which can be simplified into single-objective optimization problems using methods such as the objective constraint method, weighted sum method, and objective programming method^3^. In this paper, we focus on studying algorithms for solving single-objective optimization problems. Optimization problems can be classified into constrained optimization problems and unconstrained optimization problems according to the presence or absence of constraints. The most common way to solve constrained optimization problems is to transform them into unconstrained optimization problems using the penalty function method^4^. Optimization problems can be categorized into linear optimization problems and nonlinear optimization problems based on the characteristics of constraints and the objective function. In nonlinear optimization problems, the relationship between the constraints, the objective function, and the decision variables is nonlinear. Solving nonlinear optimization problems is typically more challenging than solving linear optimization problems. Real-life optimization problems frequently involve nonlinear optimization problems. The objective function corresponding to the optimization problem can be classified into unimodal functions and multimodal functions based on the number of extreme in the feasible domain. A unimodal function has only one global extremum in the feasible domain, which is typically the optimal solution being sought. On the other hand, multimodal functions have multiple extremes in the feasible domain, which makes it easier to get trapped in local extremes when solving for the optimal value. Many objective functions in real-world continuity optimization problems are multimodal functions. There are many classifications of optimization problems. The classification and recognition of optimization problems are helpful in selecting the appropriate optimization methods to solve them.

**Fig. 1:**
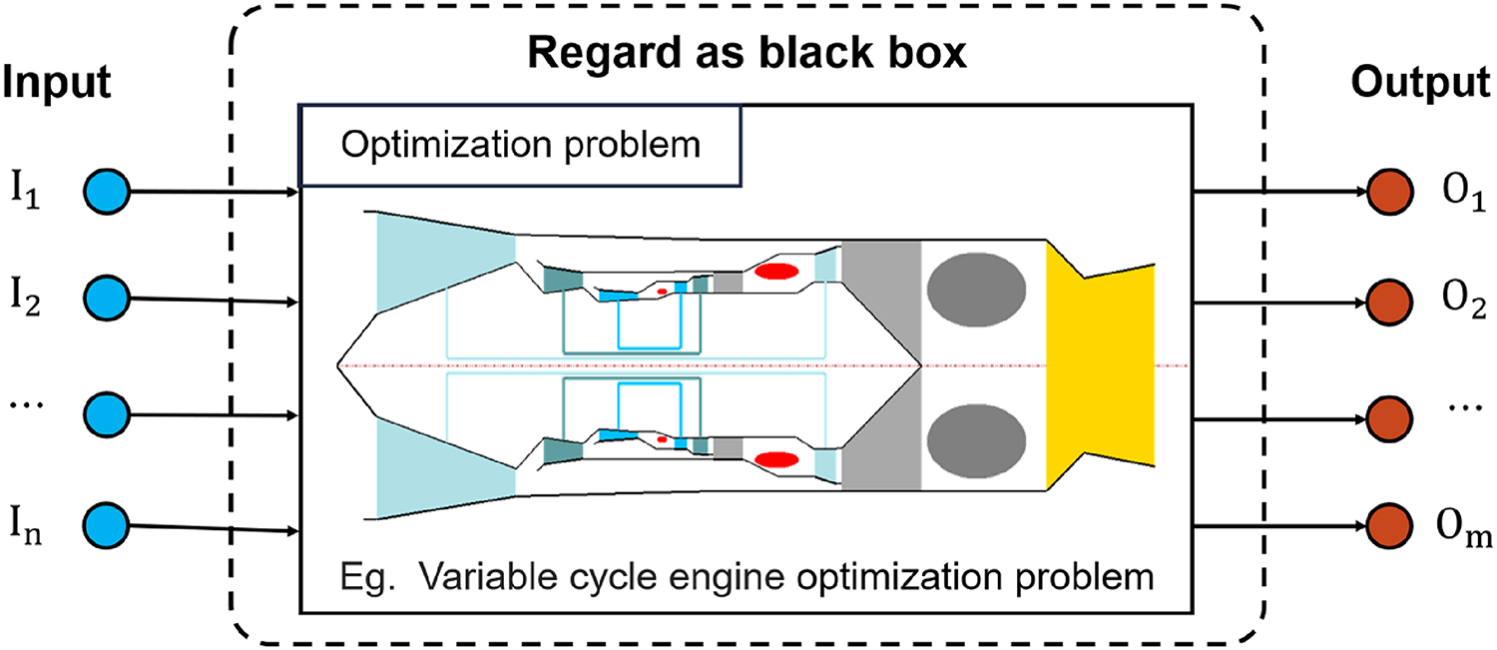
Simple/Complex optimization problems are regarded as black boxes.

In order to solve optimization problems, deterministic optimization methods such as linear and nonlinear programming methods were first developed. These methods utilize functional features or gradient information of optimization problems to find optimal solutions and are commonly employed in solving optimization problems^5^. In contrast, non-deterministic (stochastic) optimization algorithms solve optimization problems based on stochastic properties, which are characterized by their simplicity, ease of implementation, independence from gradient information during optimization, and their effectiveness in optimizing multimodal functions. Therefore, they are increasingly used to solve optimization problems across various domains. In recent decades, non-deterministic optimization algorithms have garnered significant attention and have rapidly developed. They are increasingly utilized to solve optimization problems. When using a non-deterministic optimization algorithm to solve an optimization problem, there is no need to be concerned about the form of the optimization objective function or compute the gradient information. This is because the problem being optimized can be viewed as a black box, where deterministic inputs can be provided to obtain deterministic outputs without needing to consider the internal workings of the black box. By this method, the complexity of solving the optimization problem is significantly simplified. It is only necessary to ensure that the input to the black box meets the optimization constraints, and this can be achieved through the use of a penalty function. Figure 1 illustrates the schematic diagram of the black box model.

Among the non-deterministic optimization algorithms, the most concerning is the metaheuristic algorithm. Metaheuristic algorithms are optimization algorithms used to address complex issues that cannot be solved using standard approaches^6^. Metaheuristic algorithms rely on two key search mechanisms in the optimization process: exploration and exploitation. Exploration involves visiting regions that have not been previously explored in the feasible solution globally, aiming to cover as many regions as possible. It helps in escaping local optima. Exploitation, on the other hand, involves a detailed search of explored regions, particularly those likely to contain globally optimal solutions. It is beneficial for enhancing the quality and accuracy of optimization results. Exploration and exploitation are two contrasting search processes. Emphasizing the exploration process improves the likelihood of reaching the vicinity of the actual global optimum, but the quality and stability of the optimization results may not be guaranteed. Emphasizing the exploitation process enhances the quality of the optimization results but also increases the likelihood of getting trapped in a local optimum and prematurely converging. Therefore, the essence of metaheuristic algorithms lies in balancing exploration and exploitation to obtain or approximate the optimal solution. Fortunately, nature is always the best teacher, providing numerous sources of inspiration for metaheuristic algorithms. Many researchers have developed numerous practical metaheuristic algorithms by drawing inspiration from biological behaviors or natural physical phenomena. These algorithms can be classified into the following categories based on their sources of inspiration^5,7^:

1. Evolution-based: It simulates the process of natural evolution of organisms. According to Darwin’s concept of “survival of the fittest,” superior variations and their descendants are more likely to survive and reproduce. Typical algorithms include Genetic Algorithm (GA)^8^, Differential Evolution (DE)^9^, Evolution Strategy (ES)^10^, and so on.
2. Swarm-based: It simulates the social behavior of birds, insects and animals. Typical algorithms include Particle Swarm Optimization (PSO)^11^, Sparrow Search Algorithm (SSA)^12^, Artificial Fish Swarm Algorithm (AFSA)^13^, Artificial Bee Colony (ABC)^14^, Whale Optimization Algorithm (WOA)^15^, Grey Wolf Optimizer (GWO)^16^, Chameleon Swarm Algorithm (CSA)^5^, and so on.
3. Physics-based: It simulates the laws of nature and natural physical phenomena. Typical algorithms include Gravitational Search Algorithm (GSA)^17^, Light Spectrum Optimizer (LSO)^7^, Simulated Annealing (SA)^18^, Water Cycle Algorithm (WCA)^19^, Chemical Reaction Optimization (CRO)^20^, and so on.
4. Human-based: It simulates human body systems, human brain thinking, and human behavior in society. Typical algorithms include Immune Algorithm (IA)^21^, Teaching-Learning-Based Optimization (TLBO)^22^, Artificial Neural Network (ANN)^23^, and so on.
5. Others: It includes metaheuristic algorithms that are not inspired by biological behavior or natural physical phenomena. Typical algorithms include Sine-Cosine Algorithm (SCA)^24^, Yin-Yang-Pair Optimization (YYPO)^25^, Five-Elements Cycle Optimization (FECO)^26^, and so on.

The “No Free Lunch” (NFL) theorem states that there is no single metaheuristic algorithm that can solve all types of optimization problems optimally. Each optimization algorithm has its own scope of application^27,28^. Some metaheuristic algorithms optimize well for unimodal functions but generally perform poorly for multimodal functions. Typical algorithms include GWO, WOA, and others. In contrast, some algorithms optimize well for multimodal functions but perform poorly for unimodal functions. Typical algorithms include the Firefly Algorithm (FA)^29^, Beetle Antennae Search algorithm (BAS)^30^, and so on. Therefore, integrating the existing unimodal function-solving advantage algorithms and multimodal function-solving advantage algorithms is an effective approach to improve the comprehensive optimization capability of optimization algorithms without violating the NFL theorem. As mentioned in Mirjalili’s paper^24^, the research on metaheuristic algorithms is mainly divided into three main directions: improving the current techniques, hybridizing different algorithms, and proposing new algorithms. Among them, improving the current techniques and hybridizing different algorithms are two crucial ways to improve the performance of the algorithms and broaden the use scenarios. There are numerous instances supporting this notion. For improving the current techniques, typical algorithms include IGWO^31^, MPSO^32^, IGA^33^, etc. For hybridizing different algorithms, typical algorithms include, but are not limited to BAS-PSO^34^, WPO^35^, SCCSA^36^, SA-PSO^37^, etc. However, these improvements or hybridization methods do not work well for both unimodal and multimodal problems at the same time. The purpose of this paper is to hybridize and improve two algorithms that have advantages in solving unimodal and multimodal functions, respectively, in order to improve the overall optimization performance of the proposed hybrid algorithm. It provides a competitive and viable option for solving practical optimization problems.

GWO was proposed by Mirjalili^16^et al. in 2014 and is inspired by the social hierarchy of grey wolves and the collaborative process of prey hunting. Since its introduction in 2014, GWO has gained widespread adoption in both academic and engineering fields due to its excellent optimization performance and straightforward implementation. However, Makhadmeh^38^et al. pointed out that the original GWO faces challenges such as a tendency to fall into local optima, high parameter sensitivity, and insufficient global optimization capabilities. Over the past decade, researchers have conducted extensive studies to enhance the optimization capabilities and applicability of the original GWO. In 2024, Makhadmeh and Mirjalili, the inventor of GWO, systematically summarized the development of GWOand its improved versions over the last ten years^38^. This paper organizes their research findings into two main categories for single-objective optimization improvements: Modified versions and Hybridized versions, as shown in Table 1. Modified versions have significantly enhanced the performance of GWO by incorporating various strategies such as chaos mechanisms, opposition-based learning, and adaptive strategies, making it more effective in handling complex optimization problems. However, these improvements also introduce challenges such as increased computational complexity and heightened parameter sensitivity, which require careful consideration in practical applications. Hybridized versions, on the other hand, combine GWO with other optimization algorithms to leverage their respective strengths, demonstrating exceptional performance in tackling complex, multi-objective, and large-scale optimization problems. Nevertheless, their higher implementation complexity and computational costs may limit their applicability. In the future, the development of GWO will focus on structured population design, adaptive parameter adjustment, and hybrid strategy optimization. These improvements are expected to further enhance the algorithm’s performance and applicability.

**Table 1:** GWO and related improvement strategies.

Improvement approach		Specific improvement strategies	Pros and cons	Related algorithms
Modified versions	Binary GWO	Use S-shaped and V-shaped transfer functions to convert continuous GWO to binary version for feature selection, text classification, etc.	**Pros**: suitable for binary search spaces. **Cons**: may lose diversity in continuous problems.	BGWO, BIGWO, SCGWO, RL-GWO, EOCSGWO, etc.
	Adaptive GWO	Dynamically adjust GWO parameters with adaptive mechanisms for neural network training, power system optimization, etc.	**Pros**: balances exploration and exploitation. **Cons**: increased computational complexity.	cmaGWO, etc.
	Chaotic GWO	Introduce chaotic mapping mechanisms to enhance diversity and avoid local optima.	**Pros**: improves global search ability. **Cons**: sensitive to chaotic map selection.	SCGWO, etc.
	Dynamic GWO	Dynamically adjust wolf positions or population size with nonlinear operators to enhance flexibility and tracking ability.	**Pros**: adapts to complex search spaces. **Cons**: may slow convergence in simple problems.	VAGWO, etc.
	Opposition-based GWO	Introduce opposition-based learning strategies to enhance exploration and avoid local optima.	**Pros**: enhances exploration. **Cons**: may increase computational cost.	RL-GWO, EOCSGWO, etc.
	Structured population GWO	Divide the population into subgroups to enhance diversity and search capabilities.	**Pros**: improves diversity. **Cons**: complex implementation.	AP-TLB-IGWO, etc.
	Fractional GWO	Combine fractional-order techniques for multi-view video super-resolution, natural gas and coal consumption prediction, etc.	**Pros**: handles complex systems. **Cons**: high computational cost.	FGWO, etc.
	Mutation-based GWO	Introduce mutation operations to enhance local search and convergence speed.	**Pros**: improves local search. **Cons**: risk of premature convergence.	MGWO, etc.
	Greedy strategy GWO	Combine greedy selection and crossover operations for multi-objective power flow optimization, economic load dispatch, etc.	**Pros**: fast convergence. **Cons**: may get stuck in local optima.	G-SCNHGWO, etc.
	Hybrid strategy GWO	Combine with other optimization algorithms to enhance global search and convergence speed.	**Pros**: balances exploration and exploitation. **Cons**: increased complexity.	DE-GWO, etc.
Hybridized versions	Combined with Local Search	Combine with local search algorithms to enhance local search capabilities.	**Pros**: improves local search. **Cons**: may slow global search.	MbGWOSFS, etc.
	Combined with swarm intelligence	Combine with swarm intelligence algorithms (e.g., Jaya optimizer, symbiotic organisms search) to enhance global search capabilities.	**Pros**: enhances global search. **Cons**: may increase computational cost.	DA-GWO, CS-GWO, etc.
	Combined with evolutionary algorithms	Combine with evolutionary algorithms to enhance population diversity and global search capabilities.	**Pros**: improves diversity. **Cons**: complex implementation.	EGWO-GA, etc.
	Combined with other algorithms	Combine with other algorithms for specific optimization problems.	**Pros**: tailored for specific problems. **Cons**: limited generalizability.	ELM-GWO, etc.

BAS is a metaheuristic algorithm proposed in 2017 by Jiang^30^et al., which is inspired by the foraging and mate-seeking behavior of beetles. BAS has the advantages of fewer parameters, better global search capability, being not easy to fall into local optima, and straightforward programming implementation. Due to its simple principles and efficient implementation, BAS has been widely adopted and continuously refined in both academic and engineering fields. Chen^39^ et al. systematically reviewed recent advancements in BAS, categorizing its single-objective optimization improvement strategies into four main classes, as shown in Table 2. These methods have demonstrated significant optimization results within their respective applicable scopes. Chen^39^ et al. further highlighted that integrating BAS with other high-performance algorithms represents a promising approach to enhancing its global search capabilities, indicating a fruitful direction for future research.

**Table 2:** BAS and related improvement strategies.

Improvement approach	Specific improvement strategies	Pros and cons	Related algorithms
Parameter adjustment	Adjusts step size, beetle spacing, introduces beetle populations.	**Pros**: Enhances global/local search. **Cons**: High complexity, sensitive to parameters.	VSBAS, BSAS, BASL, etc.
Adaptive mechanisms	Uses inertia weights, elite selection, fallback mechanisms.	**Pros**: Fast convergence, robust. **Cons**: Complexity, local optima risk.	BAS-ADAM, WSBAS, EBAS, ENBAS, FBAS, etc.
Hybrid heuristics	Combines PSO, ABC, FPA, GA, ACO, etc. for global/local search.	**Pros**: Combines strengths, versatile. **Cons**: High complexity, tuning needed.	BSO, BAS-PSO, BAPSO, MBAS, BAS-ABC, etc.
Deep learning	Optimizes neural networks (BP, CNN, ELM) with BAS.	**Pros**: Improves training speed/accuracy. **Cons**: High complexity, resource-heavy.	BASNNC, BASZNN, BAS-CNN, etc.

From the above analysis of GWO and BAS, it is evident that BAS boasts advantages such as a simple structure, fewer parameters, and strong global search capabilities, particularly excelling in optimizing multimodal functions, although its local search ability is relatively weak. On the other hand, GWO also features a simple structure and fewer parameters, with strong local search capabilities, but its global search ability is somewhat limited. The two algorithms complement each other’s strengths. Based on the analysis and future prospects presented in the studies by Makhadmeh^38^and Chen^39^, integrating the two algorithms with complementary characteristics and designing improvement strategies tailored to their respective features holds promise for developing a highly competitive global optimization algorithm. Therefore, this study integrates BAS and GWO, proposing improvement strategies for BAS’s antenna length update, GWO’s population summoning mechanism, and the balance between exploration and exploitation, resulting in a high-performance new algorithm named BAGWO. This paper will conduct a detailed study and discussion on the design, improvement strategies, and overall performance of BAGWO.

The main research and contributions of this paper are listed as follows:

1. A novel algorithm named BAGWO is proposed by hybridizing GWO and BAS. This algorithm replaces grey wolves with beetles and simplifies the hierarchical structure. Key improvements include the swarm position update strategy, the local exploitation frequency switching strategy, and the beetle antenna length update strategy. Experimental results demonstrate that BAGWO significantly outperforms BAS and GWO in comprehensive optimization performance, with ablation tests further confirming the effectiveness of these enhancements.
2. BAGWO incorporates three improvement strategies: it prioritizes global exploration in the early stages to increase the likelihood of approaching the global optimum, while focusing on local exploitation in the later stages to enhance the stability and precision of the optimization results. Extensive experiments validate the effectiveness of this design approach.
3. The optimization performance of BAGWO is rigorously evaluated using 24 benchmark functions from CEC2005 and CEC2017, as well as eight challenging real-world engineering problems. Statistical analysis shows that BAGWO exhibits strong competitiveness in comprehensive optimization performance and global optimization compared to other widely-used optimization algorithms.

The remainder of this paper is structured as follows. In Sect. 2, a brief overview of the fundamental principles and core characteristics of GWO and BAS is provided. In Sect. 3, the BAGWO, formed by integrating and improving GWO and BAS, is introduced, along with a detailed description of the improvement strategies, algorithm principles, and pseudocode. In Sect. 4, the optimization performance of the proposed BAGWO is tested by CEC benchmark functions, and the test results are analyzed using statistical methods. In Sect. 5, the proposed BAGWO is applied to real-world engineering problems. Finally, in Sect. 6, the proposed BAGWO is summarized, and future applications are outlined.

## Background

This section briefly introduces the BAS and the GWO, discusses the inspiration behind the two algorithms and the abstract model, and provides the necessary background knowledge for the content in Sect. 3.

### Beetle antennae search algorithm

**Fig. 2:**
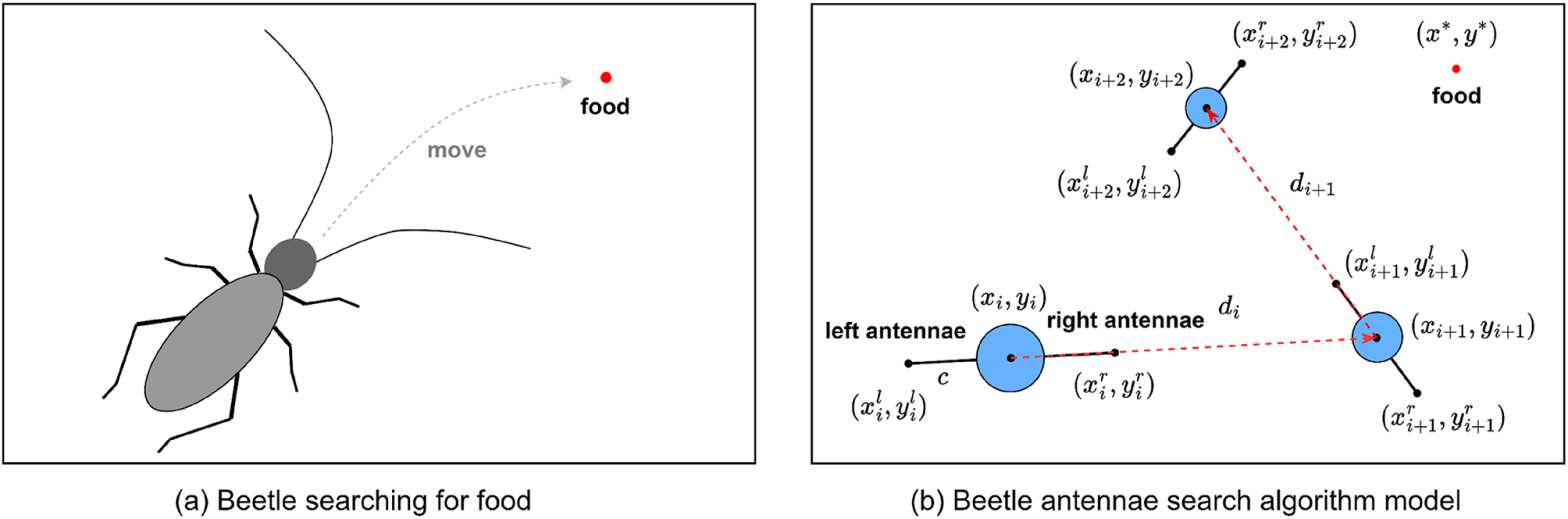
Beetle antennae search algorithm model.

As shown in Fig. 2(a), which depicts the foraging process of the beetle, the length of the beetle’s antennae tends to be longer than its body length. When foraging for food or searching for a mate, the beetles use two antennae to randomly explore the nearby area. When a higher odor concentration is detected on one side, the beetles adjust their body in that direction and move. Conversely, they adjust their body in the opposite direction and move towards the higher odor concentration until reaching the vicinity of food or a mate. Using the example of searching for food, the action steps of a beetle can be broken down as follows:

1. A beetle arrives in an area where food is available.
2. The orientation of the beetle’s head is stochastic, utilizing information from the left and right antennae to detect the concentration of food odors in the directions of both antennae.
3. The beetle rotates its body towards the side with a higher odor concentration on the antennae and moves forward a certain distance.
4. Repeat steps (2) and (3) until food is found.

The BAS optimization algorithm model can be obtained through the bionic principle of the beetle’s foraging behavior. As shown in Fig. 2(b) the beetle’s body is abstracted as a center of mass, the left and right antennae are line segments of the same length extending from the center of mass in opposite directions, and the length of a single antennae is $\:c$. The process of beetle foraging can be described as follows:

1. The initial time $\:{t}_{0}$ initializes the initial position $\:({x}_{0},{y}_{0})$ of the beetle, defines the length of the antennae at the initial time of the beetle as $\:{c}_{0}$, and the orientation $\:\theta\:$ of the beetle’s head is randomly given.
2. At time $\:{t}_{i}$, the beetle detects the food position through its antennae, and calculates the positions $\:({x}_{i}^{\text{l}},{y}_{i}^{\text{l}})$ and $\:({x}_{i}^{\text{r}},{y}_{i}^{\text{r}})$ of the left and right antennae respectively. The calculation formulas of the antennae positions are as shown in Eq. (1).

1 $$\:\begin{array}{c}\left\{\begin{array}{c}{x}_{i}^{\text{l}},{y}_{i}^{\text{l}}=p\left({x}_{i},{y}_{i},{c}_{i},{\theta\:}_{i},l\right)\\\:{x}_{i}^{\text{r}},{y}_{i}^{\text{r}}=p\left({x}_{i},{y}_{i},{c}_{i},{\theta\:}_{i},r\right)\end{array}\right.\end{array}$$

Here, $\:{\theta\:}_{i}$ is the angle of the beetle antennae relative to the coordinate system at time $\:{t}_{i}$, and the function $\:p\left({x}_{i},{y}_{i},{c}_{i},{\theta\:}_{i},m\right)$ represents the calculation function of the beetle antennae coordinate. The $\:m$ in the function input is used to judge the left and right of the antennae.

(3) Obtaining the food concentration at both antennae ends, the beetle then moves a distance $\:{d}_{i}$ along the antennae on the side with the higher food concentration and randomizes the orientation of the beetle’s head, and the concentration of the odor (also known as fitness) is calculated as shown in Eq. (2).

2 $$\:\begin{array}{c}o=o\left(x,y\right)\end{array}$$

Where $\:o\left(x,y\right)$ represents the fitness function and $\:(x,y)$ represents the position at the antennae’ end.

(4) Update the length $\:{c}_{i}$ of the beetle antennae and the step length $\:{d}_{i}$ of the beetle ‘s movement. The update formula is as shown in Eq. (3) and Eq. (4).

3 $$\:\begin{array}{c}{c}_{i}=q\left({c}_{i-1}\right)\end{array}$$

4 $$\:\begin{array}{c}{d}_{i}=g\left({d}_{i-1}\right)\end{array}$$

Where the functions $\:q\left({c}_{i-1}\right)$ and $\:g\left({d}_{i-1}\right)$ are the length update function of the beetle antennae and the step length update function of the beetle, respectively.

(5) Repeat steps (2) and (4) until the optimization results reach a certain accuracy $\:{\upepsilon\:}$, or reach the maximum number of iteration times *N*.
(6) One of the conditions for the solution to reach the iteration termination of precision $\:\epsilon\:$ is as follows.

5 $$\:\begin{array}{c}\left|o\left({x}_{i}^{\text{l}},{y}_{i}^{\text{l}}\right)-o\left({x}_{i}^{\text{r}},{y}_{i}^{\text{r}}\right)\right|\le\:\epsilon\:\end{array}$$

(7) Output the optimization result $\:({x}_{\text{b}},{y}_{\text{b}})$ and its corresponding fitness $\:o\left({x}_{\text{b}},{y}_{\text{b}}\right)$ and the actual number of iteration times $\:{N}_{a}$, the solution is finished.

BAS shows good application potential in optimization problems with its unique advantages. The main features of the algorithm include^40,41^:

1. Avoiding local optima: Compared with the gradient descent algorithm, BAS can effectively escape local optimal solutions by randomly adjusting the search direction and incorporating an appropriate step length update rule. This enhances the likelihood of discovering the global optimal solution.
2. Easy to implement: The structure and implementation process of BAS are very simple. Compared with the gradient descent algorithm, BAS does not need the gradient information of the objective function, which simplifies the calculation process.
3. Suitable for low-dimensional optimization problems: When dealing with low-dimensional optimization problems, BAS performs particularly well. Without knowing the details of the objective function, it can perform effective optimization calculations, especially for solving multimodal optimization problems.
4. Easy to integrate with other algorithms: Due to its simple form, BAS is easy to combine with other algorithms to form new hybrid optimization algorithms without significantly increasing the complexity of the algorithm.

### Grey Wolf optimizer

**Fig. 3:**
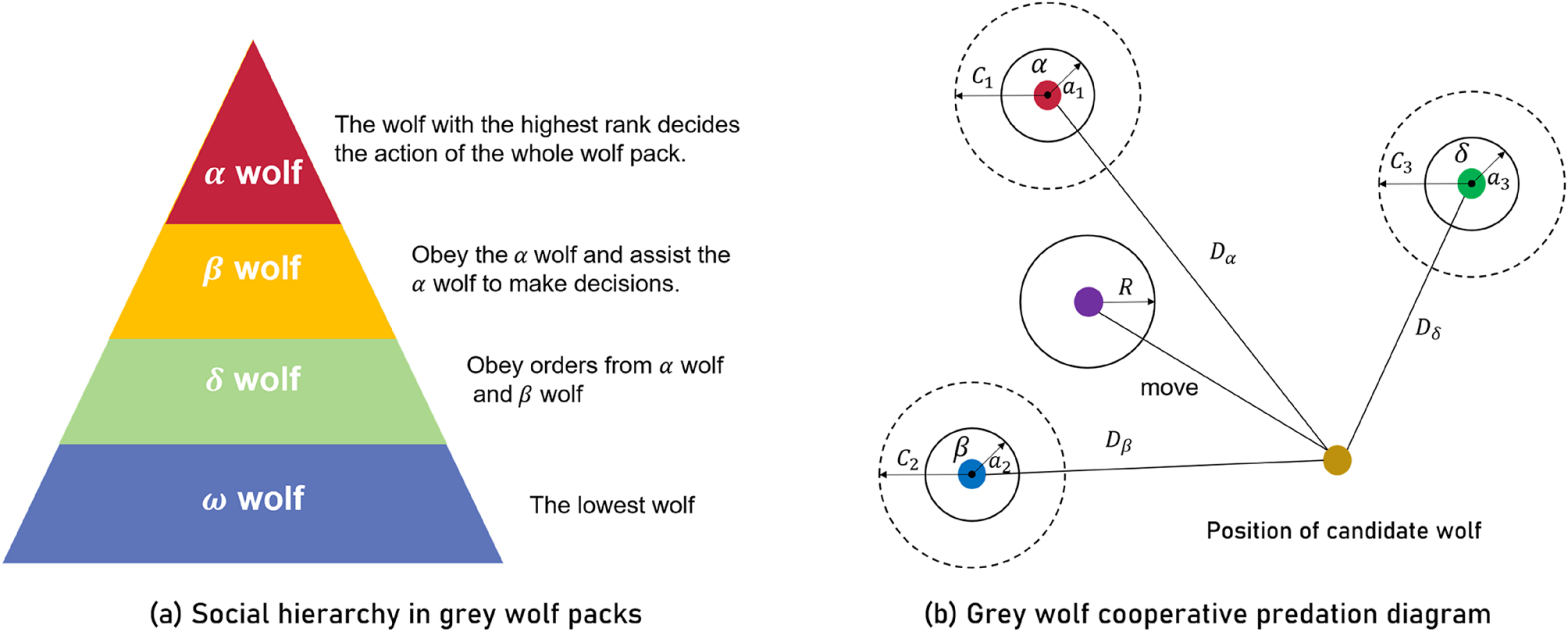
Grey wolf optimizer model^16^.

As a carnivore that feeds on small to medium-sized prey such as goats, bison, and hares, the grey wolf often hunts prey predominantly as a group. Similar to the hierarchy that exists in human society, there are different social classes within the grey wolf group, which can be classified as $\:\alpha\:$-wolf, $\:\beta\:$-wolf, $\:\delta\:$-wolf, and $\:\omega\:$-wolf according to the class, in order from high to low, as shown in Fig. 3(a)^16^. Where $\:\alpha\:$-wolf is the leader of a pack of grey wolves and is responsible for directing the hunting process of the entire pack, $\:\beta\:$-wolf is the subordinate of $\:\alpha\:$-wolf and takes orders from $\:\alpha\:$-wolf and helps $\:\alpha\:$-wolf in decision making and directing other lower ranked wolves, $\:\delta\:$-wolf is the subordinate of $\:\beta\:$- and $\:\alpha\:$-wolf, and is responsible for overseeing and directing $\:\omega\:$-wolf, and $\:\omega\:$-wolf are the lowest ranked wolves in the pack of grey wolves, and are subservient to the dominance and directing of the other ranked wolves^16,42^.

When hunting prey, grey wolf packs can be broken down into the processes of stalking and approaching the prey, encircling the prey, and attacking the prey until it is captured. The hunting process of grey wolf packs can be specifically broken down into the following steps:

1. Prey found in an area.
2. Stalking, approaching prey.
3. Chasing, surrounding, and harassing prey until it stops moving.
4. Attacking prey.

Through the bionics principle of grey wolf predation, the GWO model can be obtained. The specific algorithm details can be viewed in the article written by Mirjalili^16^ et al. in 2014. As shown in Fig. 3(b), the optimization procedure of GWO is as follows:

1. Initialize the grey wolves’ position and the parameters $\:a,\:A,\:C$ in the algorithm.
2. update the position of each grey wolf.
3. Update the fitness of each grey wolf.
4. loop procedure (2)-(3) until the maximum number of iterations is reached.
5. Output the optimal calculation results.

GWO solves the optimization problems by simulating the group hunting behavior of grey wolves. It has unique characteristics and has been widely used in practical engineering applications. The main features of the algorithm include:

1. The parameters are less, and the implementation is relatively simple: the number of parameters involved in the grey wolf algorithm is small, and the parameter adjustment is simple.
2. Excellent local search ability: The algorithm has excellent local search ability, especially suitable for the optimization of unimodal functions, and has satisfactory results.
3. Without gradient information: GWO does not need to calculate the derivative during the operation, and is suitable for the optimization of various types of non-differentiable or derivative difficult to obtain problems.
4. Poor global search ability and sensitive to the initial distribution: GWO in the global optimization problem solving is general, especially for the multimodal functions’ optimization effect is not as good as its unimodal functions’ optimization effect; In addition, GWO is sensitive to the initial distribution of the swarm, the different initial distribution of the swarm has an impact on its optimization performance.

In summary, GWO has the advantages of few parameters, relatively easy to implement, and no derivative information is required in the solution process. It is especially suitable for solving unimodal function optimization problems, but the disadvantage is that the effect of solving multimodal function problems is general, and it is sensitive to the initial distribution of the swarm.

## Proposed BAGWO

In this section, we introduce the newly proposed hybrid optimization algorithm, which combines the advantages of BAS and GWO. The comprehensive optimization performance of the hybrid algorithm is expected to surpass that of the two original algorithms. The direction of movement during the exploration of the BAS is determined by the information received at the two antennae. The optimization process does not require the use of gradient information, making it highly effective in optimizing low-dimensional problems and multimodal functions. In addition, since the BAS is simple enough, combining it with other algorithms does not significantly increase the complexity. GWO has fewer parameters, is relatively easy to implement, has good local search ability, and has an exceptional optimization effect on unimodal functions. It can be seen that BAS and GWO complement each other’s advantages and are very suitable for cross-integration to enhance the two algorithms. The enhanced algorithm can yield significant optimization benefits for both unimodal and multimodal functions.

The combination of BAS and GWO forms BAGWO. The advantages of GWO and BAS are preserved in BAGWO, with enhancements to the exploration and exploitation strategies of BAGWO. The optimization solving process within BAGWO can be divided into two distinct phases based on the collective and individual behaviors of search agents in the swarm: global exploration phase and local exploitation phase.

**Fig. 4:**
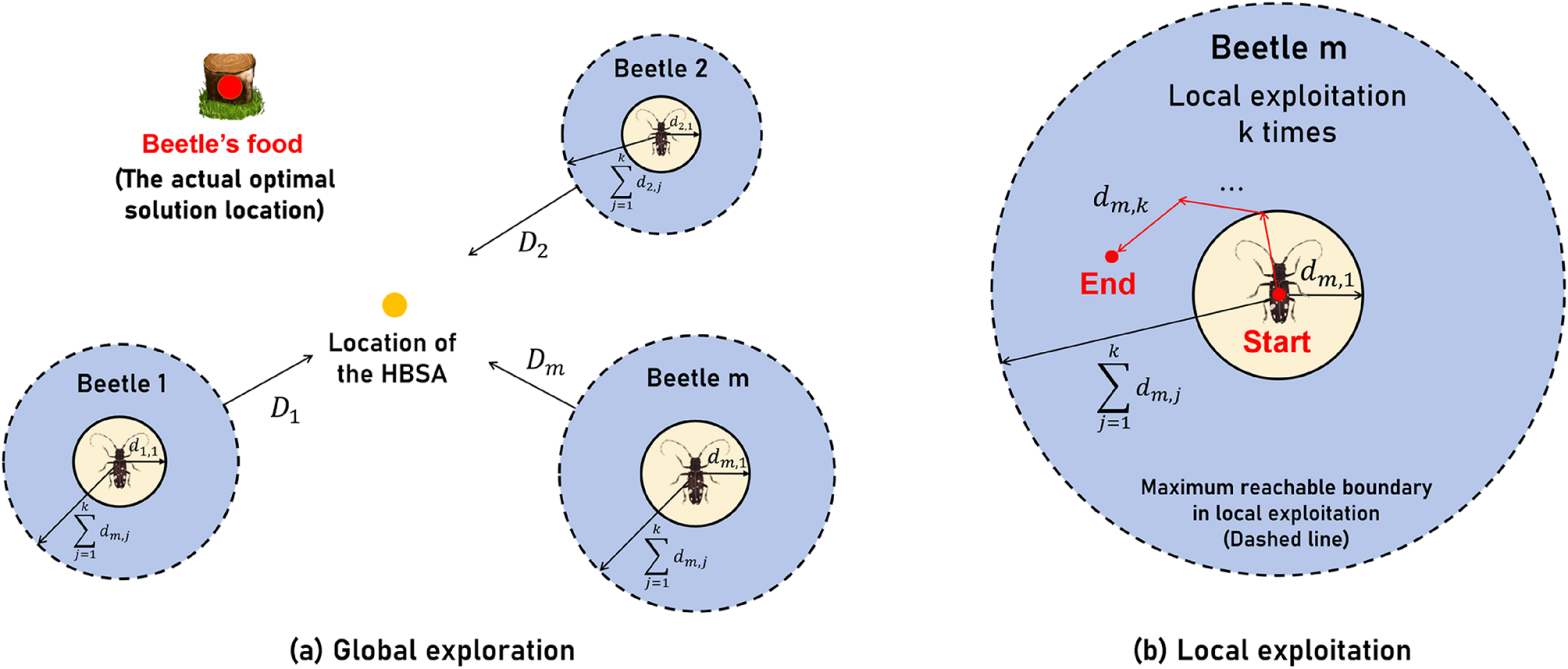
Position update in BAGWO.

**Global exploration phase:** As shown in Fig. 4(a), the schematic diagram illustrates the principle of BAGWO. In BAGWO, the search agent is replaced from a grey wolf to a beetle. The position updating method of each search agent in the respective swarm during the search for the optimal solution precisely mirrors the position updating method of a beetle in the BAS. This method ensures that the search agents consistently moves towards a non-inferior solution during the position updating process. Unlike GWO, each search agent in BAGWO is treated as an equal individual without social hierarchy. When updating its position, a search agent moves toward the direction indicated by the Historically Best Search Agent (HBSA), responding to its calling and attraction. The HBSA refers to the agent with the best fitness value since the start of the optimization process, continuously updated and tracked during the search. Through this global exploration mechanism, all search agents update their positions under the guidance of the HBSA while performing local exploitation. This approach helps focus the search on the region containing the actual global optimum and improves global optimization performance.

**Local exploitation phase:** A single search agent in the BAGWO swarm during local exploitation is illustrated in Fig. 4(b), where $\:{d}_{m,j}$ represents the step length of the search agent as it moves and $\:k$ represents the number of times for local exploitation under a certain number of iterations. It can be seen that in the local exploitation process, the search agent can only exploit in a region centered on the starting movement point with a radius of $\:\sum\:_{j=1}^{k}{d}_{m,j}$ (The subscript $\:m$ denotes the index of the search agent within the swarm, while $\:j$ represents the current number of local exploitation), and each search agent of the swarm performs such a local exploitation process. In the process of local exploitation, each search agent moves according to how beetles update their positions in BAS. They determine the direction of movement based on the fitness information received from their two antennae. While effectively exploring the local region, this approach also helps to escape from local optima. After the local exploitation process of all search agents in the swarm, the HBSA is updated and recorded to update the historical global optimal solution.

The HBSA’s position at iteration times $\:i$ is denoted as $\:{\varvec{X}}_{\text{b}}^{i}$, and the corresponding fitness is $\:{F}_{\text{b}}^{i}$, the position of the search agents in the swarm after the execution of the local exploitation process is $\:\left\{{\varvec{X}}_{1}^{i},{\varvec{X}}_{2}^{i},\cdots\:,{\varvec{X}}_{m}^{i}\right\}$, and the corresponding fitness is $\:\left\{{F}_{1}^{i},{F}_{2}^{i},\cdots\:,{F}_{m}^{i}\right\}$, the update formula for the position of HBSA $\:{\varvec{X}}_{\text{b}}^{i}$ is shown in Eqs. (6) and (7). Equation (6) represents that after all search agents in the swarm complete one round of searching, both their current fitness values and the historical best fitness value from the previous iteration are sorted. This process identifies the best fitness value $\:{F}_{\text{b}}^{i+1}$ for the current iteration. Equation (7), on the other hand, utilizes this best fitness value $\:{F}_{\text{b}}^{i+1}$ obtained from Eq. (6) to derive the corresponding historical position $\:{\varvec{X}}_{\text{b}}^{i+1}$ of the search agent (expressed through an inverse function). It is essential to note that the $\:{\varvec{X}}_{\text{b}}^{i+1}$ we aim to solve for must be based on the actual trajectory data of the BAGWO optimizer’s search process.

6 $$\:\begin{array}{c}{F}_{\text{b}}^{i+1}=\text{min}\left({F}_{1}^{i},{F}_{2}^{i},\cdots\:,{F}_{m}^{i},{F}_{\text{b}}^{i}\right)\end{array}$$

7 $$\:\begin{array}{c}{\varvec{X}}_{\text{b}}^{i+1}={f\left({F}_{\text{b}}^{i+1}\right)}^{-1}\end{array}$$

In BAGWO, not only are the characteristics of BAS and GWO combined, but also the charisma (newly proposed concept), antennae length switching strategy, and the switching strategy of the frequency of local exploitation have been researched and improved to varying degrees. These improvements enhance the comprehensive optimization performance of BAGWO. In the following subsections, the specific details of these improvements will be elaborated.

### Hybrid algorithm improvement strategy

#### The charisma and its update strategy

The charisma, derived from GWO, indicates the leadership or influence of the α-wolf over other grey wolves. In this paper, the charisma refers to the HBSA’s ability to attract search agents within the BAGWO framework, represented as a real number between 0 and 1. A charisma closer to 1 means that the HBSA strongly draws search agents towards its position, causing them to move there immediately when the charisma is 1. Conversely, as the charisma approaches 0, the HBSA’s attraction diminishes, leading search agents to return to their original positions, remaining stationary when the charisma equals 0.

For swarm intelligence optimization algorithms, the trade-off between exploration and exploitation is a crucial consideration. Exploration signifies the algorithm’s ability to conduct a global search to explore unexplored regions, while exploitation represents the algorithm’s local search capability to meticulously exploit already explored regions. The actual computational results demonstrate that emphasizing exploration in the early stage and exploitation in the later stage is conducive to improving the algorithm’s capability to discover the true optimal solution. Therefore, it is necessary to adjust the charisma based on the number of iterations. A smaller charisma should be used when the number of iterations is small, and a larger charisma when the number of iterations is large, until it reaches 1.0.

**Fig. 5:**
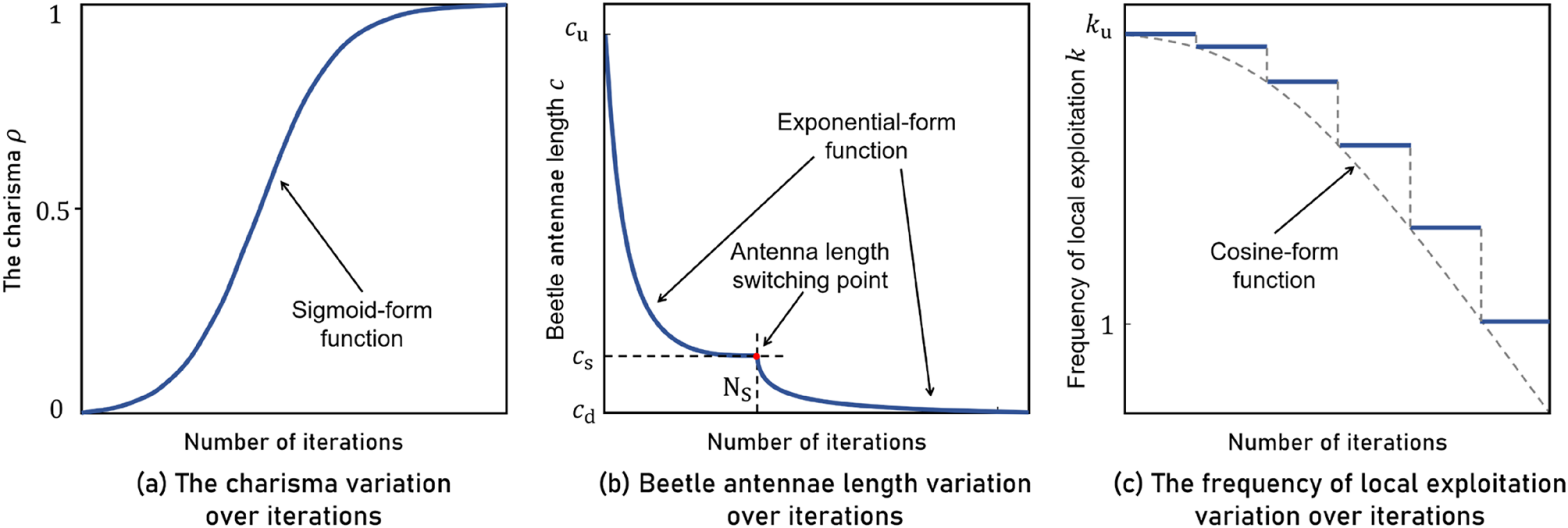
The charisma, beetle antennae length, and the frequency of local exploitation variation over iterations.

In mathematics, the sigmoid function is referred to as a growth curve due to its S-shaped curve. When the input is small, the output is close to 0, while a large input yields an output close to 1. Therefore, the sigmoid function is particularly suitable for representing the relationship between the charisma $\:\rho\:$ and the number of iterations $\:{N}^{i}$, and the functional relationship between the two is given directly in Eq. (8). $\:{N}_{\text{u}}$ in Eq. (8) represents the maximum number of iterative running times, $\:s$ represents the shape coefficient. The larger $\:s$ is, the more drastic the change of the charisma $\:\rho\:$ is, and vice versa, the gentler the change is. $\:h$ represents the final charisma, which is a parameter that determines the final level of charisma. The smaller the value of $\:h$, the larger the range of swarm aggregation at the maximum number of iterations $\:{N}_{\text{u}}$, which is beneficial for global exploration but detrimental to local exploitation, potentially affecting the stability of the optimization results. Conversely, as the value of $\:h$ approaches 1, the range of swarm aggregation becomes smaller at the maximum number of iterations $\:{N}_{\text{u}}$, which aids in local exploitation during the later stages of the algorithm and helps improve solution stability, but also increases the probability of falling into local optimum solutions. The trend of the charisma is shown in Fig. 5(a), and in the BAGWO proposed in this paper, the value of the shape factor $\:s$ is set to 100 by default, and the value of the final charisma $\:h$ is typically around 1, commonly approximated as 0.99.

8 $$\:\begin{array}{c}\rho\:={\left[1+s{\left(\frac{1-h}{s}\right)}^{\frac{{N}^{i}}{{N}_{\text{u}}}}\right]}^{-1}\end{array}$$

#### Switching strategy of antennae length decay rate

In BAGWO, the antenna length of the search agent represents the ratio of the detection and perception distance to the distance between the upper and lower bounds of the decision variables during the optimization process. This ratio is a relative value between 0 and 1. When this value is 1, it means that the distance of the search agent’s unilateral antenna length$\:\:c$ is equal to the distance between the upper and lower bounds of the decision variables. It is important to note that in the native BAS algorithm, the antenna length $\:c$ is an absolute value, which differs from the concept presented in this paper.

To enhance the accuracy of the optimization solution, it is necessary for the antennae length of the search agents to decrease progressively throughout the iterative optimization process. There are various methods to adjust the antennae length, among which the commonly used formula is shown in Eq. (9)^30^, in which $\:{c}^{i}$ represents the unilateral antennae length of the search agents when the number of iterations is $\:i$, $\:\sigma\:$ represents the decay rate of the search agents’ antennae length (It represents the rate of change in the antennae length of the search agent), and $\:{c}_{\text{d}}$ represents the minimum antennae length. Equation (9) can be simplified to the form of Eq. (10) when $\:{c}_{\text{d}}$ is set to be 0, in which $\:{c}_{\text{u}}$ represents the initial antennae length of the beetle.

9 $$\:\begin{array}{c}{c}^{i}=\sigma\:\cdot\:{c}^{i-1}+{c}_{\text{d}}\end{array}$$

10 $$\:\begin{array}{c}{c}^{i}={c}_{\text{u}}\cdot\:{\sigma\:}^{i-1}\end{array}$$

After benchmarking, it was found that there are limitations in updating the antennae length using the approach shown in Eqs. (9) and (10). Some benchmark functions are well-optimized when the Antennae Length Decay Rate (ALDR) $\:\sigma\:$ is large, while others are well-optimized when the ALDR is small. In order to solve the matching problem of the ALDR $\:\sigma\:$ is small. In order to solve the matching problem of the ALDR $\:\sigma\:$, the approach shown in Fig. 5(b) is adopted. A larger ALDR $\:{\sigma\:}_{1}$ is used before a certain iteration times $\:{N}_{\text{s}}$, and a smaller ALDR $\:{\sigma\:}_{2}$ is used after $\:{N}_{\text{s}}$. The corresponding antennae length for an iteration times of $\:{N}_{\text{s}}$ is the switching point antennae length, denoted by $\:{c}_{\text{s}}$. In the actual parameter setting, since the solution accuracy is often related to the final antennae length, and people are more sensitive to the value of antennae length than the ALDR $\:\sigma\:$, It is more intuitive to express the antennae length updating formula as a function of the final antennae length and the number of iterations as shown in Eq. (11), where $\:{c}_{\text{s}}$ can be calculated by Eq. (12), and the coefficients $\:a$ and $\:b$ in Eq. (11) represent the pre- and post- antennae length factors, which can be calculated by Eq. (13) and Eq. (14), respectively.

11 $$\:\begin{array}{c}{c}^{i}=\left\{\begin{array}{c}{c}_{\text{u}}{\left(\frac{a}{{c}_{\text{u}}}\right)}^{\frac{i-1}{{N}_{\text{s}}}}\:\:\:\:\:\:\:\:\:\:\:\:i<{N}_{\text{s}}\\\:{c}_{\text{s}}{\left(\frac{b}{{c}_{\text{s}}}\right)}^{\frac{i-{N}_{\text{s}}}{{N}_{\text{u}}-{N}_{\text{s}}}}\:\:\:\:\:\:\:\:i\ge\:{N}_{\text{s}}\end{array}\right.\:\end{array}$$

12 $$\:\begin{array}{c}{c}_{\text{s}}={c}_{\text{u}}{\left(\frac{a}{{c}_{\text{u}}}\right)}^{\frac{{N}_{\text{s}}-2}{{N}_{\text{s}}}}\end{array}$$

13 $$\:\begin{array}{c}a={{N}_{\text{u}}}^{-1}\end{array}$$

14 $$\:\begin{array}{c}b={10}^{-0.7928{{N}_{\text{u}}}^{0.5031}}\end{array}$$

There is a connection between the parameter selection of $\:{N}_{\text{s}}$ and the maximum iteration times $\:{N}_{\text{u}}$, when the maximum iteration times $\:{N}_{\text{u}}$ is small, in order to ensure enough exploration times to avoid falling into a local optimum, $\:{N}_{\text{s}}$ should take a larger value. Conversely, when the maximum iteration times $\:{N}_{\text{u}}$ is large, it can be ensured that the solution space is sufficiently explored, and a relatively small value of $\:{N}_{\text{s}}$ should be taken to ensure that the solution space is sufficiently exploited. Equation (15) is the empirical relationship between $\:{N}_{\text{s}}$ and $\:{N}_{\text{u}}$, and the middle square bracket in the formula indicates upward rounding.

15 $$\:\begin{array}{c}{N}_{\text{s}}=\left[{{N}_{\text{u}}\cdot\:2}^{-0.6342{{N}_{\text{u}}}^{0.1775}}\right]\end{array}$$

#### The frequency of local exploitation update strategy

In order to make the algorithm focus on exploration in the early stage, faster and better to reach the actual optimal solution neighborhood and to reduce the running cost of the algorithm to some extent. Another useful improvement is to couple the frequency of local exploitation with the number of iterations of the algorithm, and the function relationship between the two is in the form of cosine function, as shown in Fig. 5(c). Through this mechanism, the swarm is able to maintain extensive global exploration during the early iterations. As the iteration progresses and the charisma value $\:h$ increases, the swarm gradually shifts its focus towards local exploitation. Consequently, the frequency of local exploitation can be appropriately reduced in this phase. Local exploitation is a process of searching for the optimal solution only within the local area of the search space, and it is a concept in contrast to global exploration. The specific functional relationship is shown in Eq. (16), where $\:{N}^{i}$ represents the current iteration times, $\:{N}_{\text{u}}$ represents the maximum iteration running times, $\:{k}_{\text{u}}$ represents the local maximum exploitation times, which is a constant, and $\:k$ represents the frequency of local exploitation corresponding to the current iteration times. The square brackets in Eq. (16) represent upward rounding, so the variation rule of the frequency of local exploitation with the number of iterations is actually shown as the horizontal line in Fig. 5(c), and the number of horizontal lines is equal to $\:{k}_{\text{u}}$.

16 $$\:\begin{array}{c}k={[k}_{\text{u}}\cdot\:\text{cos}\left(\frac{\pi\:}{2}\cdot\:\frac{{N}^{i}}{{N}_{\text{u}}}\right)]\end{array}$$

### Summary of parameters in BAGWO

Summarizing the above introduction, there are a total of five parameters that need to be set when BAGWO is actually used, which are described as follows.

1. $\:\varvec{B}$, Number of search agents in the swarm: In general, the more search agents in the swarm, the better the optimization performance of the algorithm. However, this improvement comes at the cost of increased time consumption. Considering the balance between the effectiveness of the solution to the optimization problem and the time consumption, it is generally recommended that the number of search agents falls within the range of 5 to 50, with 30 being a commonly accepted value.
2. $\:{\varvec{c}}_{\mathbf{u}}$, Initial antennae length: The initial length of the search agent’s antennae is a relative value ranging from 0 to 1. The greater the value, the larger the initial exploration space. A commonly used value is 1.0.
3. $\:{\varvec{N}}_{\text{u}}$, Maximum number of iteration times: If the number of iterations exceeds $\:{N}_{\text{u}}$, the algorithm stops running and outputs the calculation results.
4. $\:\varvec{h}$, Final charisma value: The charisma when the number of iterations reaches the maximum number of iteration times $\:{N}_{\text{u}}$, which generally takes the value of 0.99.
5. $\:{\varvec{k}}_{\text{u}}$, The maximum frequency of local exploitation for each search agent: The smaller the value of $\:{k}_{\text{u}}$, the faster the algorithm optimizes the solution. However, the corresponding optimization performance will decrease to some extent. Conversely, the larger $\:{k}_{\text{u}}$ is, the better the optimization performance, but the speed of optimizing the solution will decrease. Considering the balance between the effectiveness and time consumption of the optimization problem solution, the value of the maximum frequency of local exploitation is generally recommended to be between 2 and 20.

In practical applications, the selection of algorithm parameters varies based on the specific requirements of the tasks being optimized. For tasks that are not sensitive to computation time, high configuration parameters can be selected. in this case, the optimization performance of BAGWO can be released enough. For optimization tasks that are time-sensitive, low configuration parameters should be chosen, in this case, the optimization performance of BAGWO is somewhat limited, but it still yields acceptable results.

### Computational procedures and pseudo-code of BAGWO

A detailed description of how BAGWO is formed and improved is given above, this subsection presents the detailed computational procedures and pseudo-code of BAGWO. The detailed procedures are given below.

1. Define the objective function $\:f\left(\varvec{X}\right)$ to be solved for optimization, where $\:\varvec{X}$ is the decision variable of the optimization problem, a *n*-dimensional vector, and the upper and lower bounds corresponding to the decision variable $\:\varvec{X}$ are $\:{\varvec{X}}_{\text{u}}$ and $\:{\varvec{X}}_{\text{d}}$, respectively.
2. Initialize the algorithm parameters, and assign initial values to the number of search agents $\:B$, the initial antennae length $\:{c}_{\text{u}}$, the maximum iteration times $\:{N}_{\text{u}}$, the final charisma value $\:h$, and the local initial exploration times $\:{k}_{\text{u}}$ for BAGWO.
3. The initial distribution of the swarm was sampled using the Latin Hypercube Sampling (LHS) method to obtain the initial decision variable values$\:\:{\varvec{X}}_{0}$. It should be noted that random uniform sampling is also an optional sampling method.
4. Calculate anterior antennae length coefficient $\:a$ by Eq. (13). Determine the iteration times $\:{N}_{\text{s}}$ by Eq. (15), corresponding to the transition of antennae length decay rate.
5. Calculate initial antennae length decay rate, contained within Eq. (11).
6. Update the frequency of local exploitation of the search agents $\:k$ by Eq. (16).
7. For each search agent in the swarm, update its position by the movement mode of the beetle in BAS. For any search agent in the swarm, the specific steps are as follows:
  - Randomly initialize the orientation of the search agent, use an *n*-dimensional vector to represent this orientation, and normalize it.

17 $$\:\begin{array}{c}\theta\:=\frac{\varvec{r}}{\left|\left|\varvec{r}\right|\right|}\end{array}$$

In the Eq. (17), $\:\varvec{r}$ is the generated random n-dimensional vector, $\:\varvec{\theta\:}$ is the result of normalization, the norm in the equation is the Euclidean norm.

b) Calculate the left antenna end position$\:{\varvec{X}}^{\text{l},i}$ and the right antenna end position $\:{\varvec{X}}^{\text{r},i}$ of the search agent.

18 $$\:\begin{array}{c}\begin{array}{c}{\varvec{X}}_{m,j}^{\text{r},i}={\varvec{X}}_{m,j}^{i}+{c}_{i}\varvec{\theta\:}({\varvec{X}}_{\text{u}}-{\varvec{X}}_{\text{d}})\\\:{\varvec{X}}_{m,j}^{\text{l},i}={\varvec{X}}_{m,j}^{i}-{c}_{i}\varvec{\theta\:}({\varvec{X}}_{\text{u}}-{\varvec{X}}_{\text{d}})\end{array}\end{array}$$

In Eq. (18), $\:{\varvec{X}}_{m,j}^{i}$is the position of the search agent center, $\:i$ in the superscript represents the number of current iteration times, $\:j$ represents the frequency of local exploitation, and $\:m$ represents the serial number of search agent in the swarm.

c) Then the fitness $\:f\left({\varvec{X}}_{m,j}^{\text{r},i}\right)$, $\:f\left({\varvec{X}}_{m,j}^{\text{l},i}\right)$ corresponding to the end of the left and right antennae of the search agent can be calculated.
d) Calculate the new position of the search agent according to the fitness.

1. If $$\:\text{min}\left(f\left({\varvec{X}}_{m,j}^{\text{r},i}\right),\:f\left({\varvec{X}}_{m,j}^{\text{l},i}\right)\right)<{F}_{m}^{i}$$

19 $$\:\begin{array}{c}{F}_{m}^{i}=\text{min}\left(f\left({\varvec{X}}_{m,j}^{\text{r},i}\right),\:f\left({\varvec{X}}_{m,j}^{\text{l},i}\right)\right)\end{array}$$

20 $$\:\begin{array}{c}{\varvec{X}}_{m,j+1}^{i}={\varvec{X}}_{m,j}^{i}-2{c}^{i}\theta\:\left({\varvec{X}}_{\text{u}}-{\varvec{X}}_{\text{d}}\right)s\left(f\left({\varvec{X}}_{m,j}^{\text{r},i}\right)-f\left({\varvec{X}}_{m,j}^{\text{l},i}\right)\right)\end{array}$$

2) If $$\:\text{min}\left(f\left({\varvec{X}}_{m,j}^{\text{r},i}\right),\:f\left({\varvec{X}}_{m,j}^{\text{l},i}\right)\right)\ge\:{F}_{m}^{i}$$

21 $$\:\begin{array}{c}{\varvec{X}}_{m,j+1}^{i}={\varvec{X}}_{m,j}^{i}-0.5{c}^{i}\theta\:\left({\varvec{X}}_{\text{u}}-{\varvec{X}}_{\text{d}}\right)s\left(f\left({\varvec{X}}_{m,j}^{\text{r},i}\right)-f\left({\varvec{X}}_{m,j}^{\text{l},i}\right)\right)\end{array}$$

In Eq. (20) and Eq. (21), $\:s\left(x\right)$ is the symbol function,$\:\:{F}_{m}^{i}$ represents the fitness of the $\:m$-th search agent in the global iteration times $\:i$.

e) Repeat steps a) to d) until the local exploitation is completed for $\:k$ times, $\:j=j+1$.

(8) The position $\:{\varvec{X}}_{\text{b}}^{i+1}$ and the fitness $\:{F}_{\text{b}}^{i+1}$ of the HBSA are updated according to Eqs. (6) and (7) with $\:i$ in the superscript representing the current iteration times.
(9) Summons the search agents in the swarm to move in the direction of the HBSA. For any search agent in the swarm, the formula for the movement of the search agent is as follows.

22 $$\:\begin{array}{c}{\varvec{X}}_{m}^{i+1}={\varvec{X}}_{m}^{i}+\rho\:\left({\varvec{X}}_{\text{b}}^{i+1}-{\varvec{X}}_{m}^{i}\right)\end{array}$$

(10) If $\:{N}^{i}={N}_{\text{s}}$, Calculate hind antennae length coefficient $\:b$ by Eq. (14), Update antennae length decay rate, contained within Eq. (11).
(11) Update the charisma according to Eq. (8).
(12) Update the antennae length of the search agents according to Eq. (11).
(13) Runs the above steps (6)-(12) until the maximum number of iteration times $\:{N}_{\text{u}}$ is reached or other iteration convergence conditions are satisfied.
(14) Output the final optimal result, $\:{\varvec{X}}_{\text{b}}$ and $\:{F}_{\text{b}}$.

The pseudo-code is shown in **Algorithm 1**. The MATLAB source code and other resources on BAGWO are available at https://github.com/auroraua/BAGWO.

**Figure:**
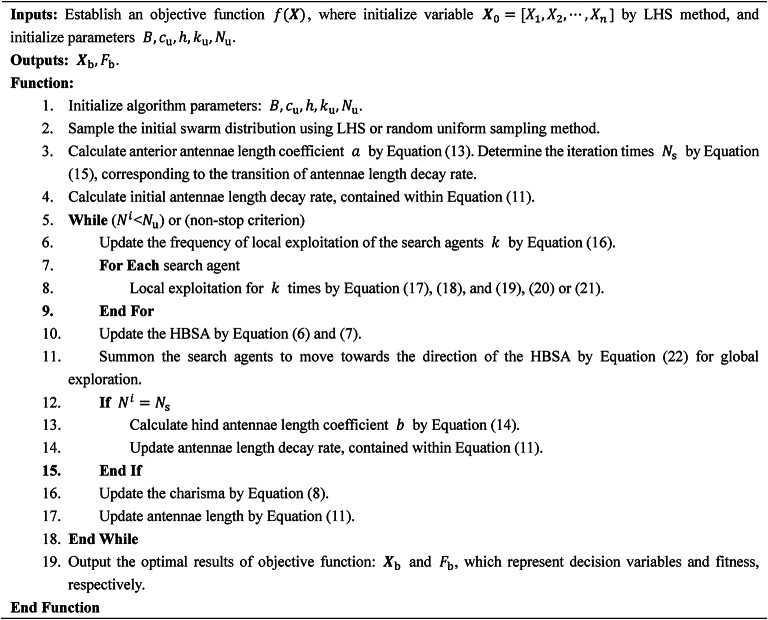
Algorithm 1: BAGWO

## Results and discussion

In this section, the proposed BAGWO and other common competitive optimization algorithms are tested on 24 benchmark functions, and the test results are analyzed using statistical analysis methods. The algorithms involved in the comparison, their parameter settings, benchmark functions selection, statistical analysis methods, and conclusions are described in detail below.

### Benchmark functions selection

The Congress on Evolutionary Computation (CEC) is held annually to explore the topic of evolutionary computation from theory to practical application. During the annual CEC meeting, a set of benchmark functions is introduced to assess the performance of different optimization algorithms in an objective and fair manner. In unconstrained single-objective optimization, benchmark functions mainly include unimodal functions, multimodal functions, hybrid functions, and compositional functions^16,36^, The differences between each function are as follows:

1. Unimodal functions: In a given interval, this type of function has only one strictly real-valued local maxima or local minima. These functions are primarily utilized to assess the convergence speed and optimization capability of the algorithm.
2. Multimodal functions: In a given interval, this type of function has multiple real-valued local maximum or local minimum. These functions are primarily used to assess the local optimal escape ability and global exploration ability of the algorithm.
3. Hybrid functions: This type of function is a hybrid of several different unimodal and multimodal functions that exhibit distinct characteristics in various regions. It is primarily used to examine the ability of optimization algorithms to flexibly switch between different search stages, search areas, and search strategies.
4. Compositional functions: This type of function is formed by combining several different unimodal or multimodal functions according to specific rules, resulting in a completely new and complex function. The optimization difficulty of these combined functions is generally greater than that of the above three categories of benchmark functions. A small but essential set of combined functions is placed within the benchmark function collection to challenge the performance of optimization algorithms and to identify those algorithms that still perform well under complex conditions.

**Table 3:** The benchmark functions in this paper.

Functions	Source	Dim	Range	$\:{\varvec{f}}_{\varvec{m}\varvec{i}\varvec{n}}$	Function type
F1	CEC 2005 F1	10/30/50/100	[−100, 100$\:{]}^{\text{d}\text{i}\text{m}}$	0	unimodal
F2	CEC 2005 F3	10/30/50/100	[−100, 100$\:{]}^{\text{d}\text{i}\text{m}}$	0	unimodal
F3	CEC 2005 F6	10/30/50/100	[−100, 100$\:{]}^{\text{d}\text{i}\text{m}}$	0	unimodal
F4	CEC 2017 F1	10/30/50/100	[−100, 100$\:{]}^{\text{d}\text{i}\text{m}}$	100	unimodal
F5	CEC 2005 F8	10/30/50/100	[−500, 500$\:{]}^{\text{d}\text{i}\text{m}}$	−418.98$\:\times\:30$	multimodal
F6	CEC 2005 F10	10/30/50/100	[−32, 32$\:{]}^{\text{d}\text{i}\text{m}}$	0	multimodal
F7	CEC 2005 F12	10/30/50/100	[−50, 50$\:{]}^{\text{d}\text{i}\text{m}}$	0	multimodal
F8	CEC 2005 F14	2	[−65.536, 65.536]	≈ 0.998	multimodal
F9	CEC 2005 F16	2	[−5, 5$\:{]}^{\text{d}\text{i}\text{m}}$	≈ −1.0316	multimodal
F10	CEC 2005 F19	3	[0, 1$\:{]}^{\text{d}\text{i}\text{m}}$	≈ −3.86	multimodal
F11	CEC 2005 F21	4	[0, 10$\:{]}^{\text{d}\text{i}\text{m}}$	≈ −10.1532	multimodal
F12	CEC 2005 F23	4	[0, 10$\:{]}^{\text{d}\text{i}\text{m}}$	≈ −10.5364	multimodal
F13	CEC 2017 F4	10/30/50/100	[−100, 100$\:{]}^{\text{d}\text{i}\text{m}}$	400	multimodal
F14	CEC 2017 F6	10/30/50/100	[−100, 100$\:{]}^{\text{d}\text{i}\text{m}}$	600	multimodal
F15	CEC 2017 F8	10/30/50/100	[−100, 100$\:{]}^{\text{d}\text{i}\text{m}}$	800	multimodal
F16	CEC 2017 F11	10/30/50/100	[−100, 100$\:{]}^{\text{d}\text{i}\text{m}}$	1100	multimodal
F17	CEC 2017 F13	10/30/50/100	[−100, 100$\:{]}^{\text{d}\text{i}\text{m}}$	1300	hybrid
F18	CEC 2017 F15	10/30/50/100	[−100, 100$\:{]}^{\text{d}\text{i}\text{m}}$	1500	hybrid
F19	CEC 2017 F17	10/30/50/100	[−100, 100$\:{]}^{\text{d}\text{i}\text{m}}$	1700	hybrid
F20	CEC 2017 F19	10/30/50/100	[−100, 100$\:{]}^{\text{d}\text{i}\text{m}}$	1900	hybrid
F21	CEC 2017 F21	10/30/50/100	[−100, 100$\:{]}^{\text{d}\text{i}\text{m}}$	2200	compositional
F22	CEC 2017 F25	10/30/50/100	[−100, 100$\:{]}^{\text{d}\text{i}\text{m}}$	2500	compositional
F23	CEC 2017 F27	10/30/50/100	[−100, 100$\:{]}^{\text{d}\text{i}\text{m}}$	2700	compositional
F24	CEC 2017 F29	10/30/50/100	[−100, 100$\:{]}^{\text{d}\text{i}\text{m}}$	2900	compositional

### Algorithms involved in the comparison and their parameter settings

In order to evaluate the performance and effectiveness of the proposed BAGWO, 14 commonly used competitive optimization algorithms are selected, including classic algorithms such as DE, GA, PSO, SA. It also includes competitive recently proposed algorithms such as GWO, IGWO, CSA, BAS, Dragonfly Algorithm (DA)^47^, Grasshopper Optimization Algorithm (GOA)^48^, Moth-Flame Optimization algorithm (MFO)^45^, Multi-Verse Optimizer (MVO)^44^, SCA, WOA. Table 4 shows the parameter settings of BAGWO and 14 other algorithms in this paper. Apart from the parameters for the BAGWO algorithm, the parameters for the other algorithms are set to the default values used or recommended in their original algorithm publications. It is important to note that due to many algorithms involved in the comparisons, adjusting their parameters would increase the complexity of the problem; therefore, their parameter settings remain unchanged throughout all the benchmark tests in this paper. For the BAGWO algorithm, setting the parameter $\:{c}_{\text{u}}$ to 1.0 aims to maximize the search range during its initial run. The value $\:h$=0.99 is the conventional default parameter mentioned in Sect. 3.1.1, while $\:{k}_{\text{u}}=10$ indicates that each search agent can perform a maximum of 10 local exploitations during the initial optimization phase. A smaller $\:{k}_{\text{u}}$ may result in insufficient local exploration, so 10 is considered a more suitable value.

Table 5 shows the feature classification of all comparative algorithms involved in this study. It can be observed that the vast majority belong to swarm-based algorithms, which are also commonly used in practical applications.

**Table 4:** All algorithms parameter settings.

Algorithm	Parameters	Algorithm category
All algorithms	$\:\text{S}\text{w}\text{a}\text{r}\text{m}\:\text{s}\text{i}\text{z}\text{e}\:B=30,\:\text{I}\text{t}\text{e}\text{r}\text{a}\text{t}\text{i}\text{o}\text{n}\text{s}\:N=500$	
BAGWO	$\:{c}_{\text{u}}=1.0,\:h=0.99,\:{k}_{\text{u}}=10$	
DE	$\:\beta\:=[0.2,\:0.8],{p}_{\text{c}\text{r}}=0.2$	Classic algorithms
GA	$\:{p}_{c}=0.8,{p}_{m}=0.05$	
PSO	$\:{v}_{\text{m}\text{a}\text{x}}=6,{w}_{\text{m}\text{a}\text{x}}=0.9,{w}_{\text{m}\text{i}\text{n}}=0.6,{c}_{1}=2,{c}_{2}=2$	
SA	$\:{\tau\:}_{f}={10}^{-10}$	
BAS	$\:{\sigma\:}_{\text{d}}=0.95,\:{\sigma\:}_{\text{d},\text{m}\text{i}\text{n}}=0.001,\:{\sigma\:}_{{\updelta\:}}=0.95,\:{d}_{0}=3.0,\:{\sigma\:}_{0}=0.8$	Recently proposed algorithms
CSA	$\:{\uprho\:}=1.0,{p}_{1}=2.0,{p}_{2}=2.0,{c}_{1}=2.0,{c}_{2}=1.8,\alpha\:=4.0,\beta\:=3.0,\gamma\:=2.0$	
DA	$\:\beta\:=1.5$	
GOA	$\:{c}_{\text{m}\text{i}\text{n}}=0.00004,{c}_{\text{m}\text{a}\text{x}}=1.0$	
GWO	$\:a=\left[\text{2,0}\right]$(The variable$\:a$decreases linearly from 2 to 0.)	
IGWO	$\:a=\left[\text{2,0}\right]$(The variable$\:a$decreases linearly from 2 to 0.)	
MFO	$\:a=[-1,-2]$(The variable$\:a$decreases linearly from − 1 to −2.)	
MVO	$\:{WEP}_{\text{m}\text{a}\text{x}}=1.0,{WEP}_{\text{m}\text{i}\text{n}}=0.2$	
SCA	$\:a=2$	
WOA	$\:a=\left[\text{2,0}\right],\:b=1$(The variable$\:a$decreases linearly from 2 to 0.)	

**Table 5:** Feature classification of all algorithms.

Features	Name
Evolution-based	DE, GA
Swarm-based	BAS, CSA, DA, GOA, GWO, IGWO, MFO, PSO, WOA, BAGWO
Physics-based	SA
Others	MVO, SCA

### BAGWO optimization performance evaluation and comparison

In this subsection, the optimization performance of the proposed BAGWO is evaluated using the CEC benchmark functions, and the test results are compared with 14 other commonly used optimization algorithms to comprehensively assess the optimization performance of the BAGWO. In the process of evaluation and analysis, statistical analysis methods are used to quantitatively analyze and evaluate the results.

#### Comparison of calculation results between BAGWO and other algorithms

Among the 24 benchmark functions selected in this paper, the input dimensions of the five benchmark functions F8–F12 are fixed, while the input dimensions of the 19 benchmark functions F1–F7 and F13–F24 are variable. The size of the input dimension represents the number of decision variables. In the comparative analysis of the test results in this section, the dimension of the benchmark functions with variable input dimension is set to 30, which is also the number of dimensions often selected in many similar works. In Table 3, detailed information on all benchmark functions was provided, where F1–F4 are unimodal, F5–F16 are multimodal, F17–F20 are hybrid, and F21–F24 are compositional functions, and Table 4 contains the parameter settings for all comparison algorithms participating in the study. In order to minimize the impact of random factors in each optimization process, each benchmark function is repeated 30 times when evaluating the optimization performance of the algorithm. and the average value and standard deviation of the calculated data are used to objectively represent the optimization result of the optimization algorithm on a specific benchmark function. The optimization problems addressed in this paper aim to minimize the value. Therefore, the lower the average value, the better the optimization performance of the algorithm, and the smaller the standard deviation, the greater the numerical stability of the optimization algorithm. The use of average value and standard deviation can reduce the influence of random factors on the calculation results to a certain extent. However, it should be noted that the larger outliers of the calculation results may deteriorate the average value and standard deviation. The boxplot can display the median, quartiles, and outliers in the data. Therefore, the box plot can be used as a valuable tool for comprehensively and intuitively analyzing and comparing the calculated data. However, in order to objectively and quantitatively analyze and evaluate the performance of the algorithms, statistical analysis methods will be mentioned and utilized later.

**Fig. 6:**
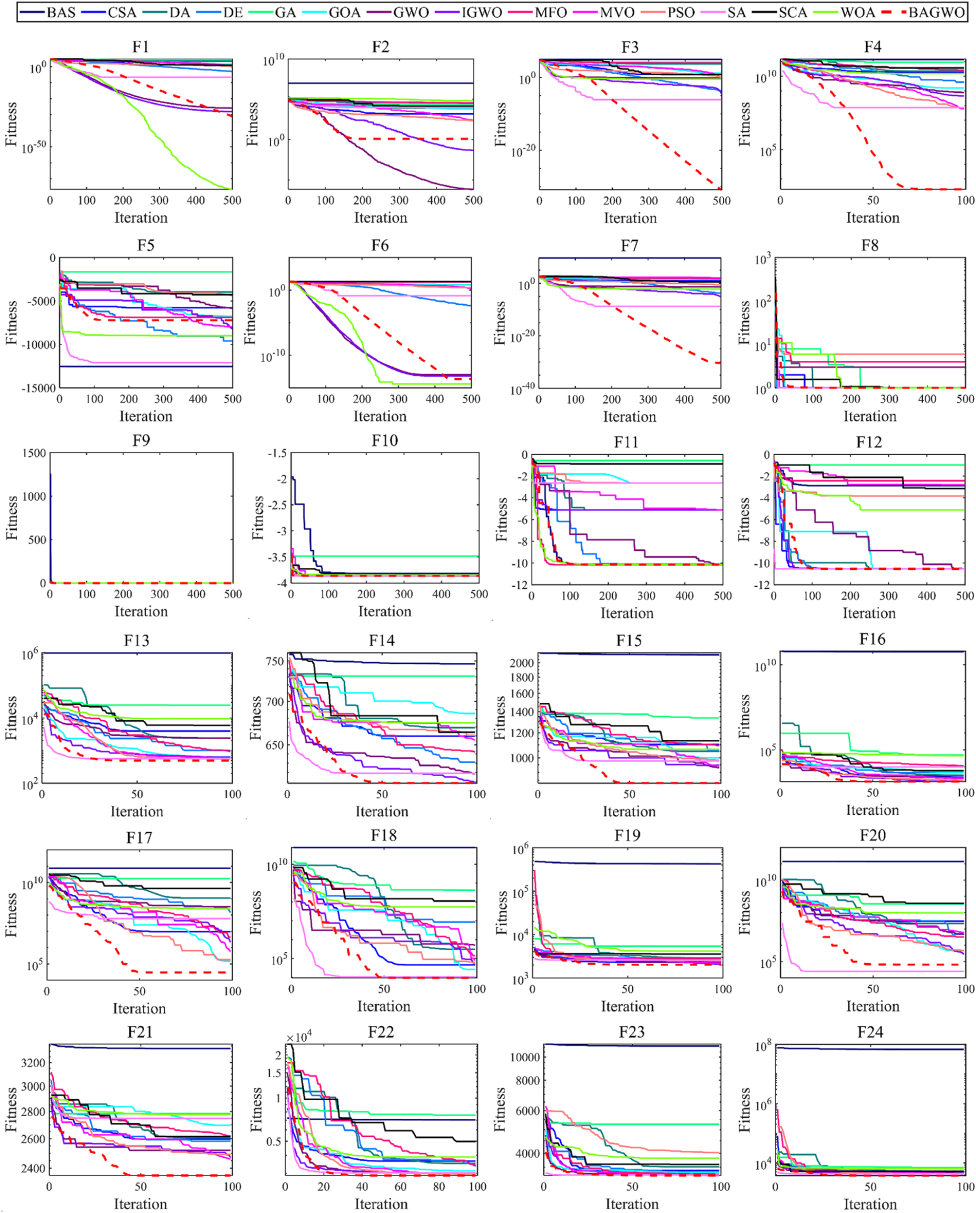
Comparison of the optimization performance of the BAGWO with 14 other algorithms across 24 benchmark functions when the function dimension is 30 (F8–F12 input dimensions fixed).

As depicted in Fig. 6, the comparison diagram illustrates the optimal fitness calculation process of 15 optimization algorithms, including BAGWO, for 24 benchmark functions. The calculation results of BAGWO are represented by red dotted lines in the figure. From the qualitative analysis of the comparison curve data in the graph, it can be seen that BAGWO has the best comprehensive optimization performance, especially in the 8 benchmark functions of F3, F4, F7, F8, F15, F17, F19 and F21. The optimization results are significantly better than those of other optimization algorithms. In addition, the optimization effect is in a dominant position in the 11 benchmark functions of F9, F10, F11, F12, F13, F14, F16, F20, F22, F23 and F24. Moreover, the optimization effect is also significant in other benchmark functions not explicitly mentioned. In Fig. 6, it can also be observed that BAGWO demonstrates superior convergence compared to the other 14 algorithms. It can rapidly approach the global optimal value area within a small number of iterations, thus validating the effectiveness of the “exploration and development” strategy proposed in this paper. In addition, it can be observed that the comprehensive optimization effect of BAGWO is superior to that of GWO, BAS and IGWO, further confirming the effectiveness and outstanding performance of BAGWO.

**Fig. 7:**
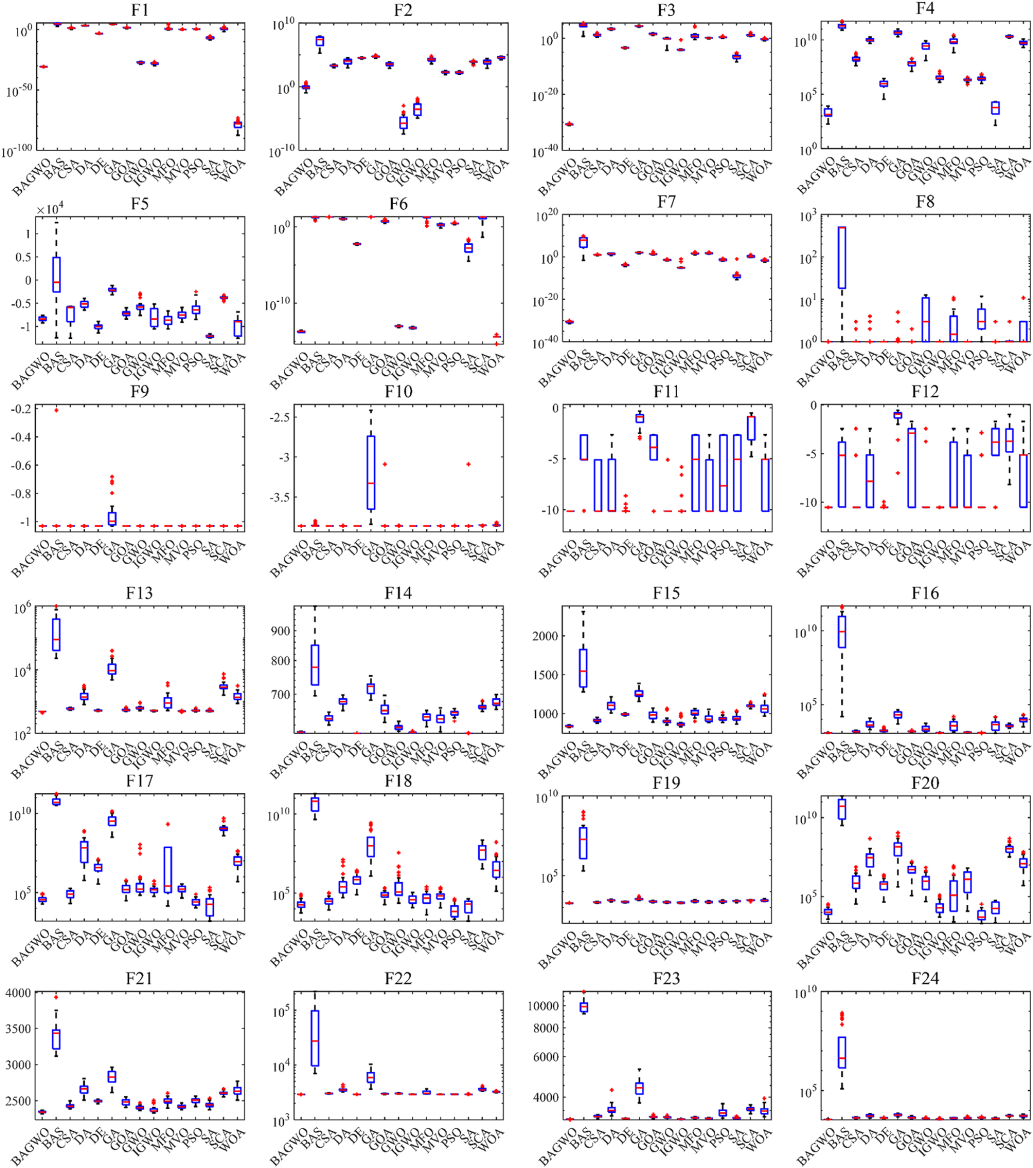
Box plot analysis for benchmark functions F1–F24.

Figure 7 shows the boxplot of the optimization results of the algorithms participating in the comparison across 24 benchmark functions. The reason for using boxplot is that they can intuitively present the data distribution of multiple optimization results from different algorithms across various benchmark functions, as well as key statistical information such as the median and interquartile range. In the same boxplot, the central red horizontal line indicates the median of the optimization results; the lower its position, the better the average optimization performance of the algorithm. The length of the box in the vertical direction reflects the degree of dispersion of the optimization results: a longer box signifies poorer stability of the optimization results, which corresponds to worse performance of the respective algorithm on the current benchmark function. In simple terms, algorithms with a lower red line position and shorter boxes in the boxplot demonstrate better performance on the current benchmark function, providing a visual and qualitative assessment of the comprehensive optimization performance of the algorithms. In qualitative analysis, it can be observed from the boxplot that the optimization results of BAGWO are concentrated. The median and average values are low in the figure, indicating superiority over other algorithms in the calculation results of most benchmark functions. In **Table** **A.1** and **Table** **A.3** of Supplementary Material, the average value and standard deviation of the calculation results for 15 algorithms across 24 benchmark functions are presented, using the same data source as Fig. 7, it is evident that the optimization results of BAGWO are significantly superior in most benchmark functions. Based on the data in Figs. 6 and 7, the data in the Supplementary Material, and the corresponding qualitative analysis conclusions, it can be seen that the optimization performance of BAGWO is superior to that of the other 14 algorithms included in the comparison. BAGWO demonstrates better accuracy, stability, and convergence speed in solving the optimization problem. However, to ensure the validity of this conclusion, the next section will further quantitatively analyze the calculation results across various dimensions.

#### Convergence behavior analysis

**Fig. 8:**
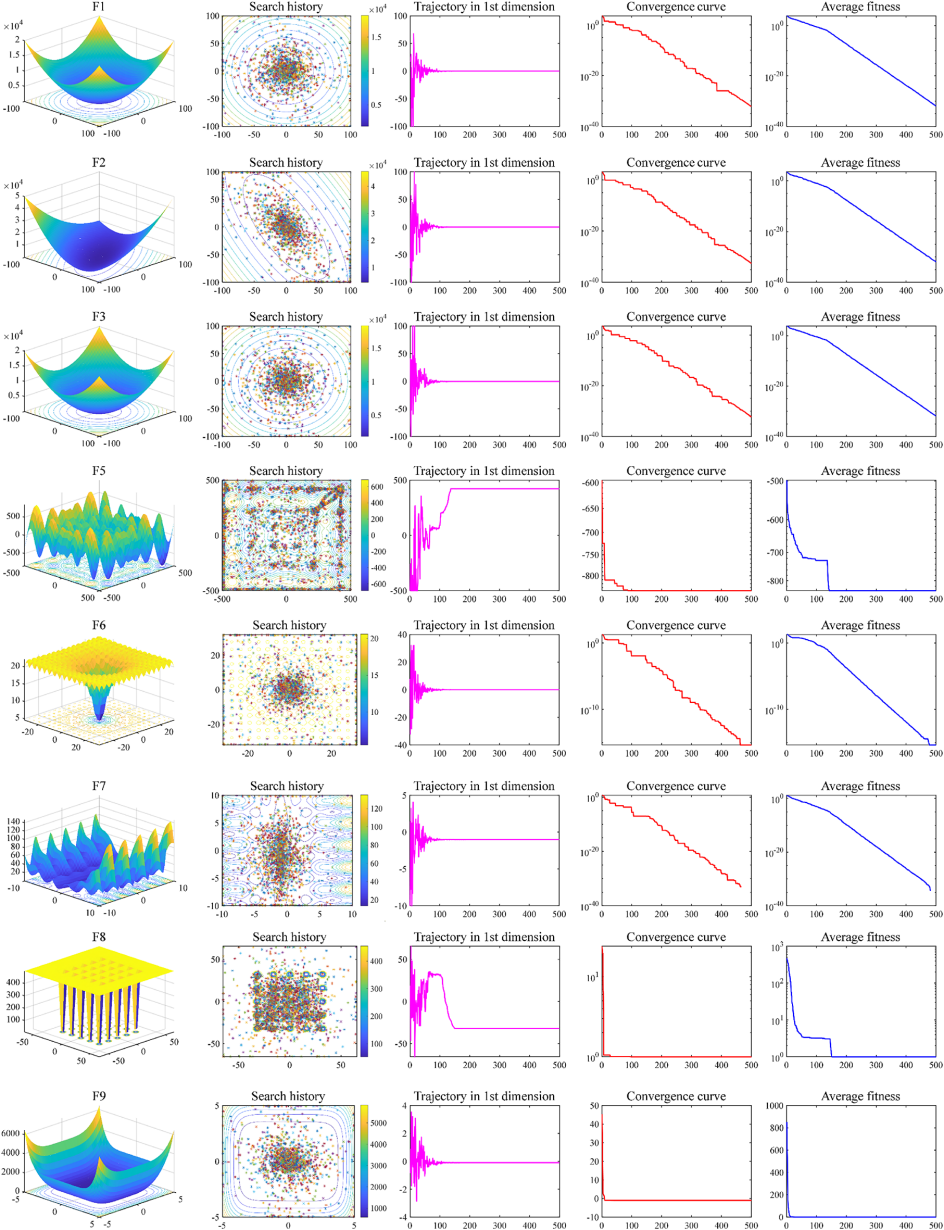
Search history and trajectory of the first particle in the first dimension (F1–F3, F5–F9).

**Fig. 9:**
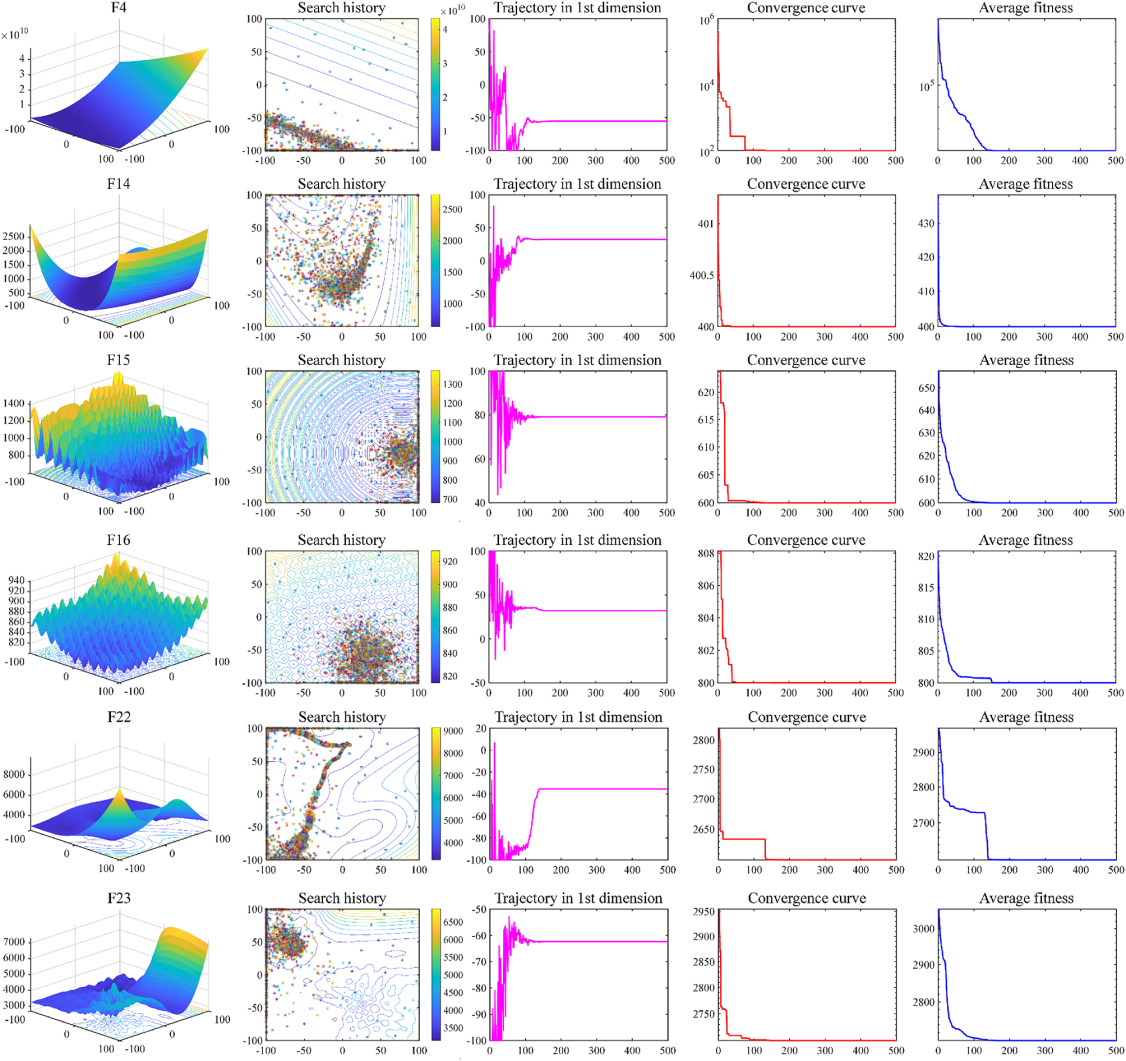
Search history and trajectory of the first particle in the first dimension (F4, F14–F16, F22, F23).

Improvement strategies for the BAGWO are discussed in Sect. 3 of this paper, including the improvement of the charisma, the improvement of the switching strategy for ALDR, the improvement of the frequency of local exploitation, and the improvement of the initial distribution of the swarm. The conclusions of the qualitative analysis in the previous subsection validate the effectiveness of the improvement. This subsection will analyze how the improvement works. A total of 14 benchmark functions are selected here, namely F1–F9, F14–F16, F22, F23. Figures 8 and 9 depict the trends of various parameters as the number of iterations increases during the optimization process of BAGWO in these benchmark functions. The meanings of the subgraphs represented in the figures, in order of columns from left to right, are as follows: the subgraph of the benchmark functions dimension is 2D, the contour subgraph shows the historical optimization process of the swarm in the two-dimensional space, the historical trajectory subgraph of the one-dimensional optimization variable, the convergence process subgraph of the best fitness, and the convergence process subgraph of the average fitness of the swarm. From the one-dimensional history trajectory graph in the third column and the swarm search history graph in the second column in Figs. 8 and 9, it can be seen that the search agents in the swarm change their positions drastically when the number of iterations is small. During this drastic change of position, the swarm mainly focuses on exploration, which corresponds to the left tail of the function curve of the sigmoid function of the charisma. At this time, the charisma value is small, and the frequency of local exploitation is also large. Therefore, the probability of the swarm moving in the region near the optimal solution is high. As the number of iterations increases, the rate of change in position rapidly levels off, at this time the charisma value gradually increases, the swarm is more and more focused on exploitation, and it gradually tends to exploit the region near the global optimum until the global optimal solution is found. From the above analysis, it can be seen that the BAGWO enables the swarm to quickly locate the region where the optimal solution is found in the exploration-oriented process. The swarm then exploits this region in detail during the exploitation-oriented process, achieving a balanced exploration and exploitation in BAGWO. This balance enhances accuracy, stability, and convergence speed.

To further verify the convergence characteristics of BAGWO, this study employs a quantitative analysis method to compare the convergence speed of BAGWO with other comparative algorithms across 24 benchmark functions. Based on the convergence criteria derived from the iterative solving process, the algorithm is considered to have reached a stable convergence state when the relative error between the current iteration result $\:{R}_{L}$ at step $\:L$ and the final iteration result $\:{R}_{N}$ exactly does not exceed 0.1% of the relative error between the initial iteration result $\:{R}_{0}$ and the final iteration result $\:{R}_{N}$. This convergence criterion, as shown in Eq. (23), provides a quantitative basis for the convergence analysis of the algorithm.

23 $$\frac{R_L-R_N}{R_0-R_N}\leqslant0.001$$

**Fig. 10:**
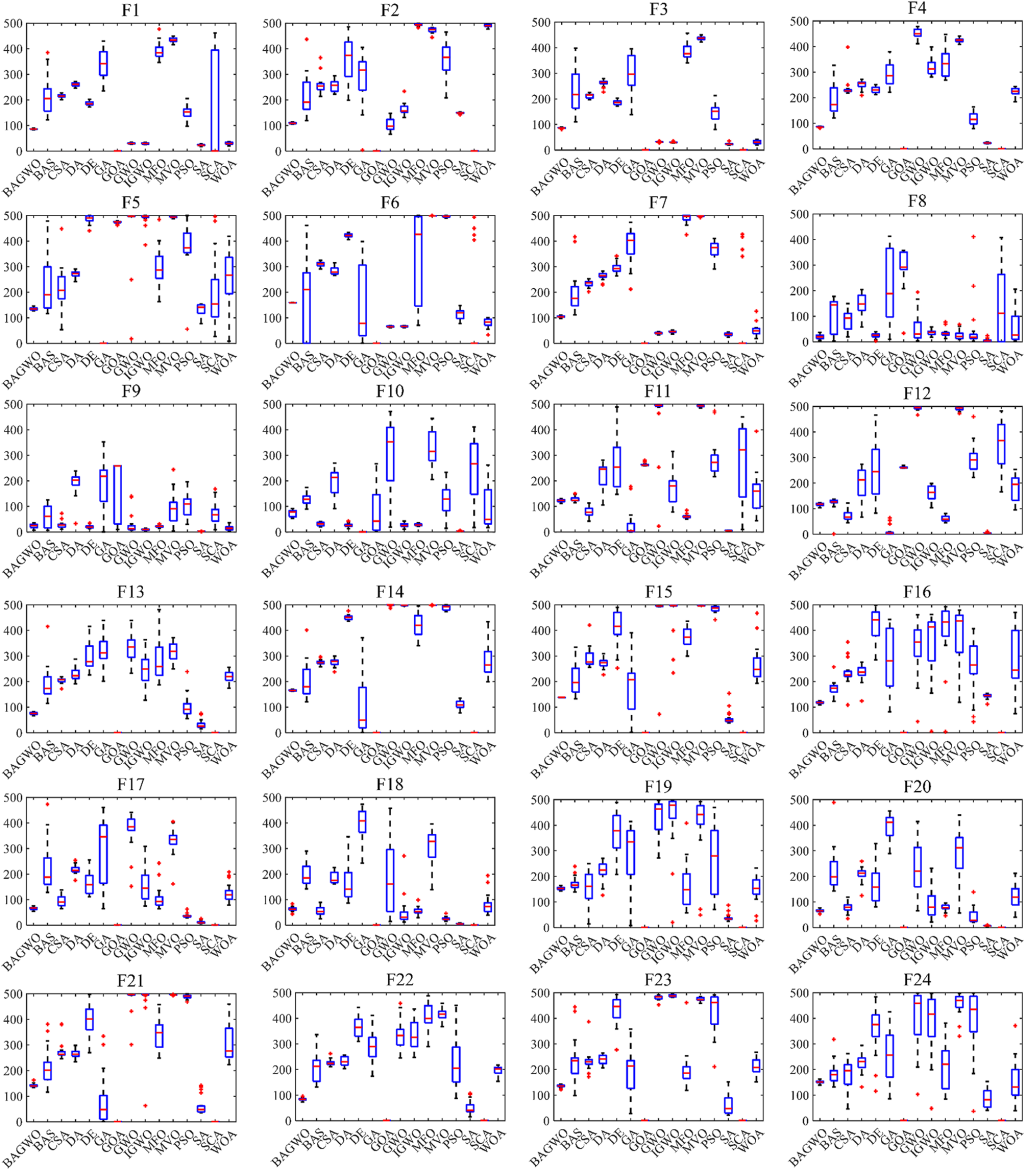
Boxplot of convergence speeds of the algorithms.

The box plot shown in Fig. 10 illustrates the minimum number of iterations required for different algorithms to reach a stable convergence state when solving 24 benchmark functions, calculated according to Eq. (23). It should be noted that the data in the figure represent the results of each algorithm independently running 30 times on each benchmark function. Furthermore, the convergence speed discussed in this paper is based on the stability of the algorithm’s final output results, rather than the actual global optimal solution of the optimization problem. As can be seen from Fig. 10, BAGWO is able to achieve stable convergence within 100–200 iterations in most cases (with a maximum iteration limit of 500), which is a reasonable and efficient convergence speed. An excessively fast convergence speed may lead the algorithm to get stuck in a local optimal solution, while a slow convergence could affect the stability and accuracy of the solution. It is noteworthy that the number of iterations required for BAGWO to achieve stable convergence on the same benchmark functions demonstrates a high degree of stability, with its standard deviation significantly lower than that of other comparative algorithms, which is beneficial for the stability of the final optimization results. This stable and efficient convergence characteristic exhibited by BAGWO is closely related to the three improvement strategies proposed in Sect. 3.1. This conclusion is further validated by the experimental results in Sect. 4.4 and 4.5.

#### Optimization performance comparison in different dimensions

**Fig. 11:**
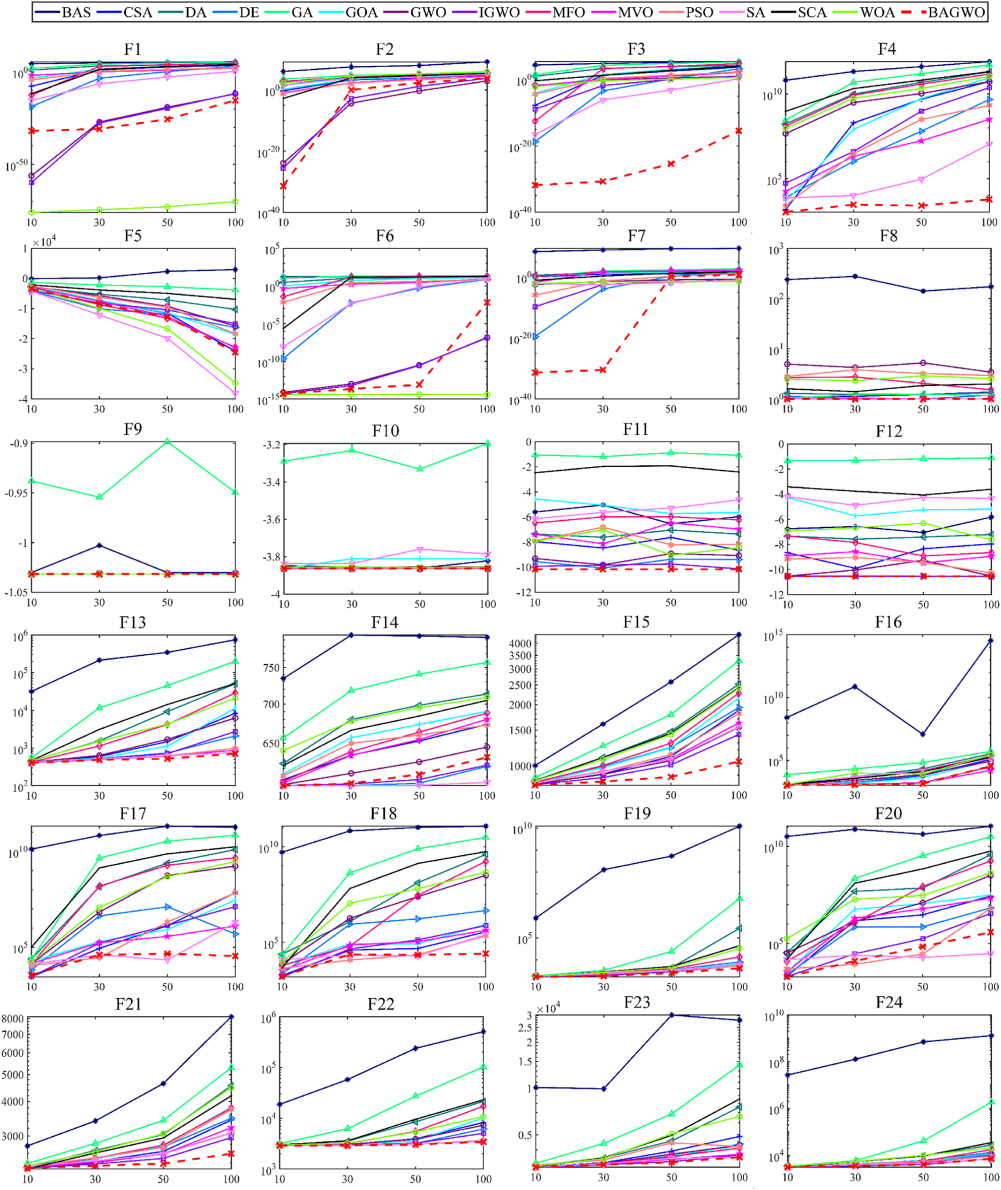
Comparison of optimization performance of various algorithms under different dimensions.

The previous qualitative analysis of the benchmark functions’ results was conducted with a dimension of 30. However, in practical applications, the dimension of the optimization problems varies based on the number of decision variables. Therefore, it is essential to investigate whether the optimization performance of BAGWO deteriorates compared to other algorithms across different dimensions. This research is crucial for the future practical implementation of BAGWO. In this subsection, the optimization effects of 15 algorithms with dimensions of 10, 30, 50, and 100 on 24 benchmark functions are studied, respectively. The results are shown in Fig. 11, it can be observed that in F1, F4, F8, F9, F10, F11, F12, and F13, the optimization effect of BAGWO remains essentially unchanged, in the remaining benchmark functions, although the optimization effect is slightly deteriorated (except for F5), the degree of deterioration is not significant compared to other algorithms participating in the comparison, and the ranking of optimization ability remains basically unchanged.

A detailed statistical result of the number for algorithms that have a dominant solving position among benchmark functions across different dimensions is presented in Table 6. For more detailed data on different algorithms in different dimensions, please refer to **Tables** **A.2**, **Tables** **A.3**, **Tables** **A.4**, and **Tables** **A.5** in the Supplementary Material. Based on the above analysis, when the number of decision variables in the optimization problems changes, the relative optimization performance of BAGWO remains essentially unchanged compared to other algorithms. This stability is crucial for the practical application of BAGWO in optimization problems.

**Table 6:** Results and comparison of different algorithms on 24 benchmark functions (fixed dim results are included).

Algorithm name	Dominant quantity			
	Dim = 10	Dim = 30	Dim = 50	Dim = 100
BAGWO	14	14	12	12
BAS	0	0	0	0
CSA	1	0	0	0
DA	0	0	0	0
DE	3	0	0	0
GA	0	0	0	0
GOA	0	0	0	0
GWO	0	1	1	1
IGWO	2	1	1	1
MFO	0	0	0	0
MVO	0	0	0	1
PSO	1	4	2	1
SA	0	2	6	5
SCA	1	0	0	0
WOA	2	2	2	3

#### Comparative analysis of BAGWO time-consuming

**Fig. 12:**
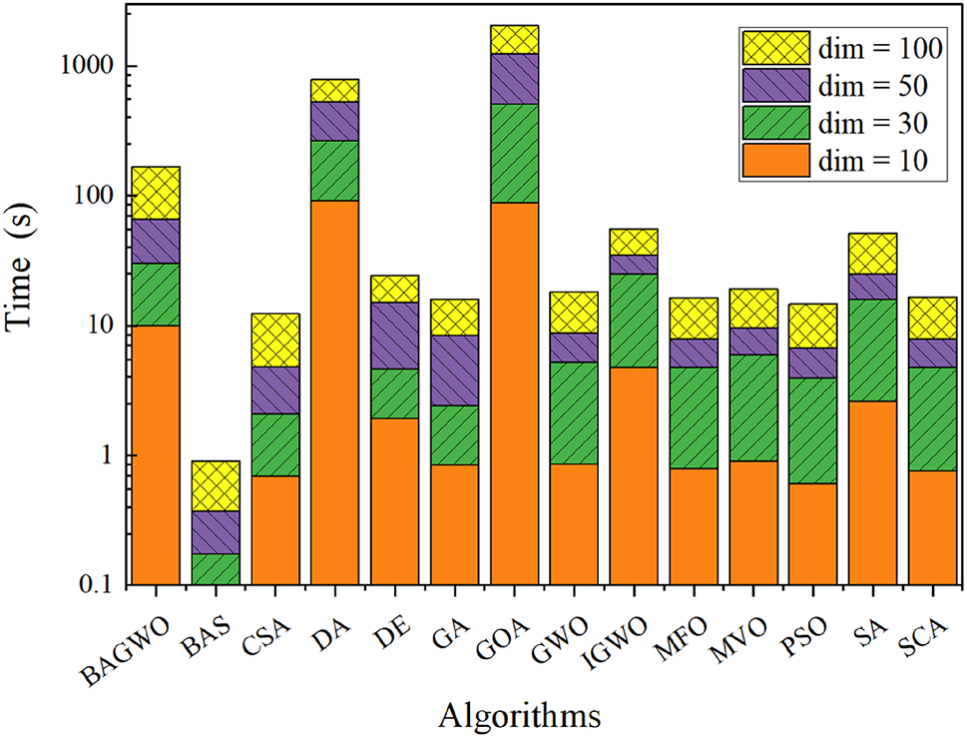
Average computation time for algorithms to calculate F1–F24 under different dimensions.

In the solution of optimization problems, in addition to the need for optimal performance, computational time consumption is also a crucial consideration for optimization problems with high time complexity or significant computational costs. Figure 12 illustrates the time taken by the 15 algorithms included in the comparison to complete a round of F1–F24 benchmark functions based on the parameter settings outlined in Table 4 and various dimensions. It can be observed that as the number of dimensions increases, the time consumption of various algorithm optimization solutions also increases. Among these solutions, GOA takes the most time. The proposed BAGWO does not offer an advantage in terms of time consumption under the current parameter settings. However, this does not indicate that BAGWO is not superior in computation time. The optimization algorithm’s time consumption is often related to the number of calls to the optimization objective function in a round of iterations. The frequency of local exploitation in Sect. 3.3 of this paper directly affects the number of calls to the optimization objective function, and thus influences the time consumption of the optimization computation process.

**Fig. 13:**
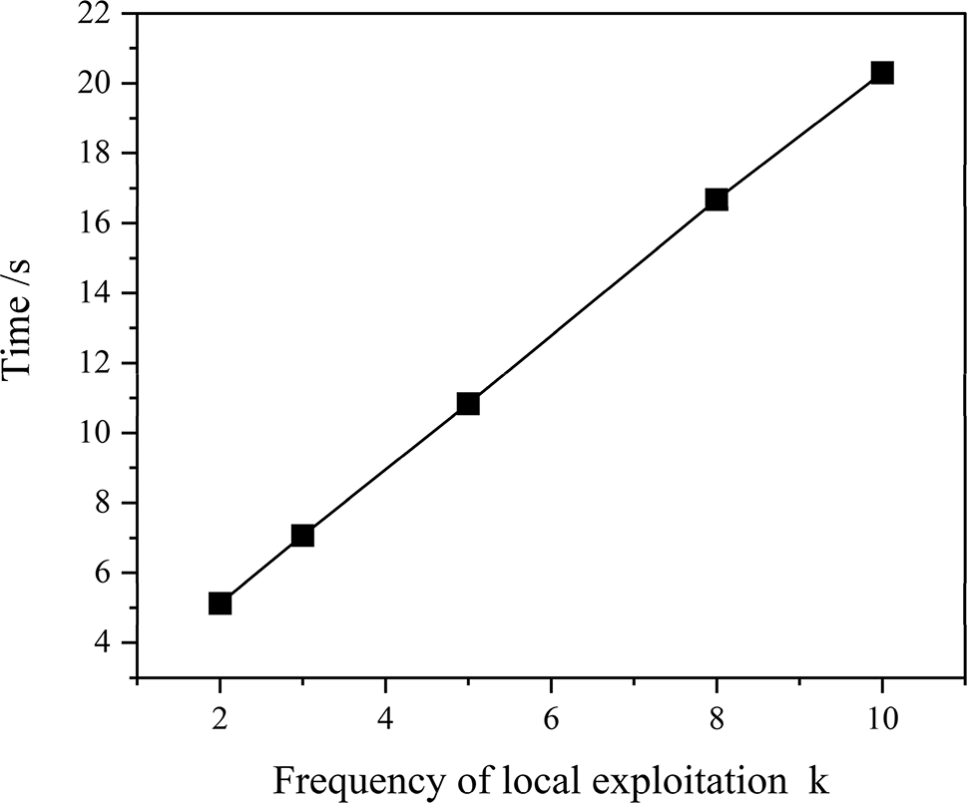
Average time consumption under different frequencies of local exploitation conditions.

**Fig. 14:**
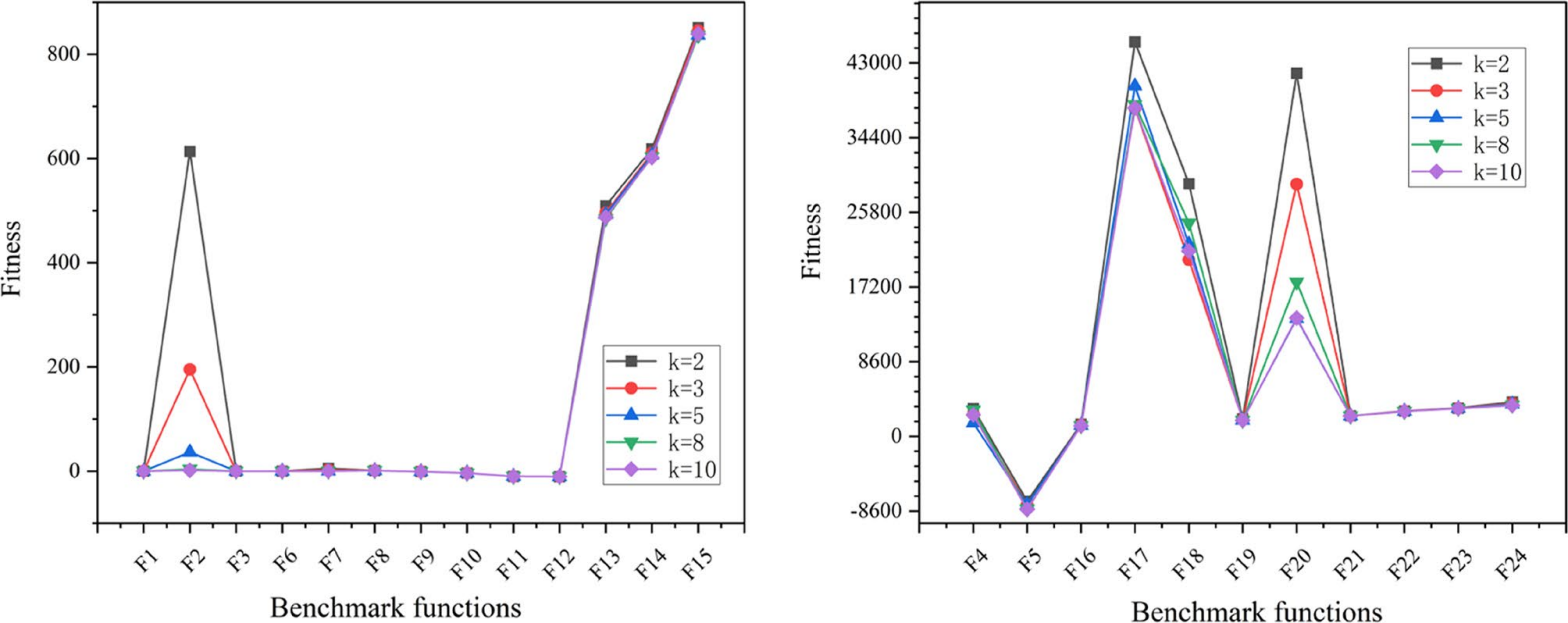
Average optimization results under different frequencies of local exploitation conditions.

In the parameter setting of Table 4, the frequency of local exploitation $\:{k}_{\text{u}}$ of BAGWO is 10. When the value of $\:k$ decreases, the time consumption of the optimization process will decrease, but this may affect the accuracy and stability of the optimization algorithm. Therefore, under the five local exploitation cases of $\:k=2$, $\:k=3$, $\:k=5$, $\:k=8,\:k=10$, the time consumed by BAGWO to complete a round of F1–F24 benchmark functions and the optimization results of F1–F24 benchmark functions are taken respectively. In the optimization calculation process, each benchmark function runs 30 times. Figure 13 illustrates the impact of the frequency of local exploitation $\:k$ on consumption time. The square of the correlation coefficient of the fitting line of the curve is $\:{R}^{2}=0.9999$, indicating that the frequency of local exploitation$\:\:k$ has a strong linear relationship with the consumption time. Figure 14 illustrates the average value of 30 optimization results of benchmark functions under different frequencies of local exploitation. It can be seen that the frequency of local exploitation significantly influences the optimization results of F2, F17, F18, and F20, while having a relatively minor impact on the optimization results of other benchmark functions. Therefore, for time-sensitive optimization problems, selecting a lower frequency of local exploitation $\:k$ can maintain a good optimization effect with a high probability while reducing time consumption. For general time-insensitive optimization problems, the parameter settings in Table 4 can be utilized.

During the execution of optimization algorithms, a critical factor affecting computational cost is the number of times the algorithm calls the objective function of the optimization problem. Therefore, it is necessary to specifically compare and analyze the frequency of objective function calls between BAGWO, BAS, and GWO. For consistency in analysis, assuming that the maximum number of iterations is $\:{N}_{\text{u}}$and the number of search agents in the swarm is $\:B$.

For the BAS algorithm, as it is not a swarm intelligence algorithm and requires calculating the objective function values at both ends of the left and right antennae during each iteration, the total number of objective function calls is $\:2{N}_{\text{u}}$. Regarding the GWO algorithm, since each search agent invokes the objective function only once per iteration, the total number of objective function calls amounts to $\:{BN}_{\text{u}}$. As for BAGWO, which incorporates features of BAS, each search agent invokes the objective function twice during a single computation. However, due to the adoption of a cosine-based local exploitation update strategy, the calculation of how many times the objective function is called per iteration by a single search agent becomes more complex. It can be demonstrated that when $\:{k}_{\text{u}}\ge\:1$, the average number of times the objective function is called by each search agent in a single iteration falls between $\:{k}_{\text{u}}+1$ and $\:{2k}_{\text{u}}$. Consequently, the total number of objective function calls for BAGWO ranges from $\:{(k}_{\text{u}}+1){BN}_{\text{u}}$ to $\:{{2k}_{\text{u}}BN}_{\text{u}}$. Thus, for any given optimization problem, the number of objective function calls made by BAGWO is at least $\:{(k}_{\text{u}}+1)B/2$ times that of the BAS algorithm and at least $\:{k}_{\text{u}}+1$ times that of GWO. This analysis further confirms that BAGWO is suitable for optimization problems where computational cost is less of a concern.

### Non-parametric statistical analysis

The optimization performance of the algorithms is compared by the average value and standard deviation of the test results of the benchmark functions, this is a very intuitive comparison method. However, the conclusion obtained by this method is not comprehensive, because the test results of each algorithm after each execution may not be consistent, this may cause the existence of outliers to cause the average value and standard deviation of the calculate results to be high. In addition, the optimization performance of the algorithms through the average value and standard deviation are compared by the test results of a single benchmark function. It is hard to say that the proposed BAGWOis necessarily superior to a certain algorithm. Therefore, it is very necessary to study whether it is certain that the proposed algorithm is superior to other algorithms^43^. Fortunately, the mentioned problems can be solved by statistical analysis.

Because the distribution of the test results of the optimization algorithms are unknown and the number of samples are relatively small, non-parametric test methods are used in statistical analysis, among which Friedman test and Wilcoxon rank-sum test are the most commonly used.

#### Wilcoxon rank-sum test

The Wilcoxon rank-sum test is utilized to determine if there is a significant difference between the medians of two related samples. It is employed to assess the performance of the proposed algorithm against other algorithms. The test is conducted at a significance level of 5%. After conducting the test, we obtain a p-value for each comparison. The p-value represents the probability of observing the current data or more extreme data under the premise that the null hypothesis (there is no difference in the medians of the two results) holds. When comparing BAGWO with another algorithm, if the p-value is less than the significance level of 0.05, we reject the null hypothesis, which means that there is a statistically significant difference in the median performance between BAGWO and that algorithm. In the Wilcoxon rank-sum test statistical results, the symbol “+” indicates that BAGWO has better optimization performance than the algorithms being compared, the symbol “=” indicates that BAGWO has comparable optimization performance to the algorithms being compared, and the symbol “-” indicates that BAGWO has worse optimization performance than the algorithms being compared. In Table 7, the optimization performance comparison results of BAGWO are presented in comparison with other algorithms across various dimensions (10, 30, 50, 100). It can be seen from the table that the optimization performance of BAGWO is significantly superior to that of other algorithms compared across various dimensions.

**Table 7:** Wilcoxon rank-sum test results.

Compared algorithms BAGWO vs.	Different dimensions (+/=/-)			
	10	30	50	100
BAS	24/0/0	24/0/0	24/0/0	24/0/0
CSA	22/1/1	23/1/0	22/2/0	24/0/0
DA	24/0/0	24/0/0	24/0/0	24/0/0
DE	17/5/2	19/3/2	19/3/2	20/3/1
GA	24/0/0	24/0/0	24/0/0	24/0/0
GOA	24/0/0	24/0/0	24/0/0	24/0/0
GWO	23/0/1	23/0/1	22/0/2	22/0/2
IGWO	17/4/3	18/4/2	20/1/3	19/3/2
MFO	21/3/0	20/4/0	20/3/1	22/2/0
MVO	24/0/0	24/0/0	23/1/0	23/0/1
PSO	24/0/0	21/0/3	20/2/2	23/1/0
SA	21/2/1	20/1/3	18/1/5	16/3/5
SCA	23/1/0	24/0/0	24/0/0	24/0/0
WOA	22/1/1	21/0/3	20/0/4	20/0/4
Overall (+/=/-)	**310/17/9**	**309/13/14**	**304/13/19**	**309/12/15**
Dominant ratio	**92.26%**	**91.96%**	**90.48%**	**91.96%**

#### Friedman test

The Friedman test is used to determine if the overall distribution, represented by more than two groups of samples, is the same. It is used to test for differences between the test results of the proposed BAGWO and other algorithms. The algorithm’s optimization performance is ranked on average, and the test is conducted at a 5% significance level. Like the Wilcoxon rank-sum test, here, strict statistical judgments are also made through the p-value of the optimization result comparison data. If the p-value is less than the 5% significance level we set (*p* < 0.05), we can reject the null hypothesis, which means that we have sufficient evidence to show that the optimization performance distributions of different algorithms are not the same, and the ranking advantage of BAGWO is not merely by chance. Table 8 presents the optimization performance comparison results of BAGWO relative to other algorithms across different dimensions (10, 30, 50, 100, respectively). It can be seen from the table that the optimization performance of BAGWO ranks first in various dimensions.

The results of the Friedman test and Wilcoxon rank-sum test quantitatively demonstrate that the proposed BAGWO shows excellent comprehensive optimization performance. Combined with the qualitative analysis conclusions in the previous section, it is evident that compared with other algorithms participated in the comparison, BAGWO exhibits excellent accuracy, stability, and convergence speed. However, it should be noted that although the CEC benchmark functions provide a fair and unified testing platform for evaluating algorithm performance, these functions do not cover a wide range of unconstrained optimization problems. Of course, this is understandable, as testing on all possible optimization problems is impractical. Therefore, the conclusion regarding the superior performance of BAGWO in this section still requires further validation and substantiation through testing on more optimization problems in the future.

**Table 8:** Friedman test results.

Compared algorithms	Average rank under different dimensions				Average rank	Ranking
	10	30	50	100		
BAGWO	**2.223**	**2.115**	**2.242**	**2.151**	**2.183**	**1**
BAS	14.351	14.183	14.086	13.996	14.154	15
CSA	5.917	6.734	6.896	6.933	6.620	6
DA	10.62	10.725	10.874	10.763	10.746	12
DE	4.389	5.563	5.946	6.59	5.622	4
GA	13.093	13.854	14.028	14.074	13.762	14
GOA	8.418	8.302	8.123	8.4	8.311	10
GWO	7.609	6.993	7.026	6.697	7.081	8
IGWO	3.679	3.708	4.081	4.199	3.917	2
MFO	6.928	7.944	8.38	8.932	8.046	9
MVO	7.697	6.643	6.417	6.108	6.716	7
PSO	7.589	6.374	5.896	5.634	6.373	5
SA	7.044	5.375	4.832	4.607	5.465	3
SCA	10.675	11.872	11.992	11.989	11.632	13
WOA	9.766	9.615	9.182	8.928	9.373	11

#### Sensitivity analyses of BAGWO parameters

As indicated in Sect. 3.2, BAGWO has three specific parameters: Initial antennae length $\:{c}_{\text{u}}$, Final charisma value $\:h$, and the maximum frequency of local exploitation for each search agent $\:{k}_{\text{u}}$. In previous computations, these parameters were set to $\:{c}_{\text{u}}=1.0$, $\:h=0.99$, and $\:{k}_{\text{u}}=10$. To investigate the impact of these three distinct parameter settings on BAGWO’s performance, sensitivity analyses were conducted on $\:{c}_{\text{u}}$, $\:h$, and $\:{k}_{\text{u}}$ individually. During these analyses, all other conditions remained consistent with those described in Sect. 4.3.1: number of search agents in the swarm $\:B=30$, a maximum number of iterations $\:{N}_{\text{u}}=500$, and tests performed on 24 selected CEC benchmark functions with a dimensionality of 30. Each benchmark function was executed 30 times to minimize the effect of random errors.

For the sensitivity analysis of $\:{c}_{\text{u}}$, the following values were examined: 0.05, 0.1, 0.2, 0.4, 0.6, 0.8, and 1.0. For $\:h$, the values were 0, 0.2, 0.4, 0.6, 0.8, 0.9, 0.99, 0.999, 0.9999, and 0.99999. For $\:{k}_{\text{u}}$, the values tested were 1, 2, 4, 6, 8, and 10. When analyzing the sensitivity of one parameter, the other two parameters were kept at their original settings as specified in Table 4.

**Fig. 15:**
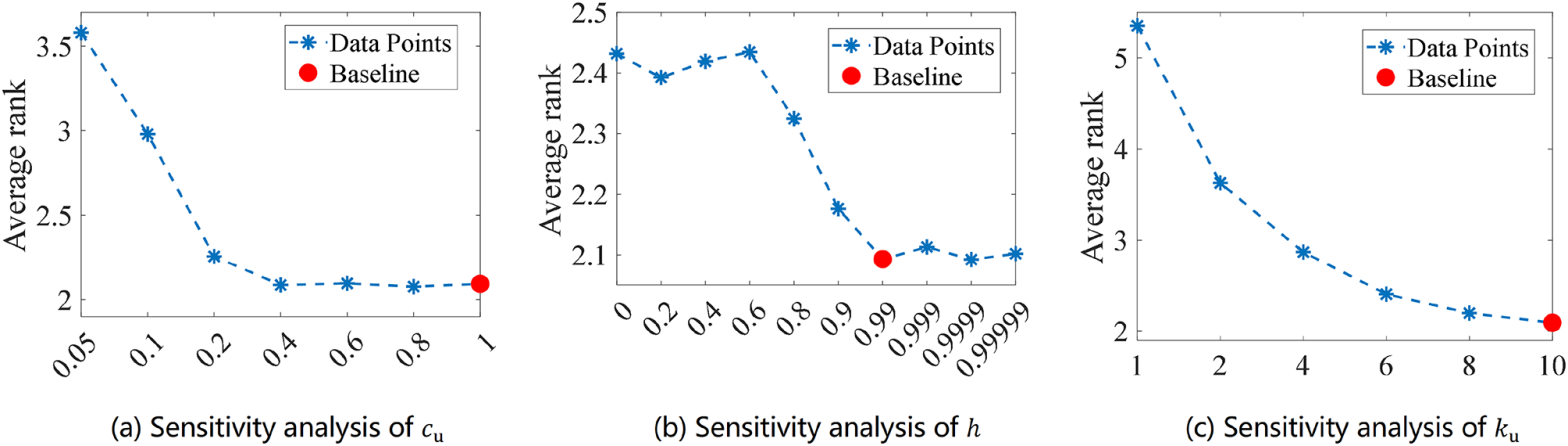
Sensitivity analyses of BAGWO parameters based on Friedman test average rankings.

The results from these analyses were processed using the Friedman test and compared with the statistical analysis outcomes presented in Table 8 for the average rank at dimension of 30, which serves as the Baseline. The findings, illustrated in Fig. 15, reveal that variations in the values of $\:{c}_{\text{u}}$, $\:h$, and $\:{k}_{\text{u}}$ significantly affect the overall optimization performance of BAGWO. Specifically, changes in parameter $\:h$ have the least impact, while changes in $\:{c}_{\text{u}}$ have a moderate effect, and $\:{k}_{\text{u}}$ exhibits the most significant influence. Notably, when $\:{c}_{\text{u}}$ is less than 0.2, the overall optimization performance of BAGWO decreases substantially. Similarly, when $\:h$ is below 0.99, there is a slight decline in performance, and for $\:{k}_{\text{u}}$, smaller values lead to a more pronounced decrease in performance. Moreover, the parameters selected in Table 4 demonstrate excellent performance for BAGWO. This analysis provides valuable insights into the selection of parameters for BAGWO.

### Ablation experiments on BAGWO

The ablation experiment is a crucial method commonly used to verify the effectiveness of algorithm and model components^49^. Its characteristic lies in the application of the control variable method, specifically disabling and removing certain functions and modules. Subsequently, a comprehensive performance comparison is carried out between the original algorithm (or model) and the ablated version, precisely evaluating the specific contributions and achievements of these functions and modules to the improvement of the system performance. This article focuses on the BAGWO, which is a hybrid and improved version of the GWO and the BAS. Three improvement strategies which mentioned in subsection 3.1 have been integrated. In this subsection, a comprehensive analysis of these three improvement strategies is conducted through ablation experiments, and the specific impacts of these improvements on the optimization performance of the BAGWO algorithm are analyzed via quantitative statistical analysis methods.

In this subsection, in order to conduct the ablation experiment on BAGWO, three improvement strategies will be removed from BAGWO respectively, as follows:

1. The BAGWO with “The charisma and its update strategy” removed is called BAGWO_A, aiming to investigate the impact and contribution of this strategy on the performance of BAGWO;
2. The BAGWO with “Switching strategy of antennae length decay rate” removed is named BAGWO_B, which is used to explore the influence and contribution of this strategy on the performance of BAGWO;
3. The BAGWO with “The frequency of local exploitation update strategy” removed is named BAGWO_C, in order to examine the impact and contribution of this strategy on the performance of BAGWO.

Based on the 24 benchmark functions selected in Sect. 4.1, we perform optimization calculations for BAGWO, BAGWO_A, BAGWO_B, and BAGWO_C at different decision variable dimensions. Each algorithm is run 30 times on each benchmark function. Subsequently, the Wilcoxon rank-sum test is used to perform statistical analysis on the optimization calculation results, and the test is conducted at a 5% significance level. The symbol and meaning of the Wilcoxon rank-sum test are the same as those in Sect. 4.4.1. The statistical results are shown in Table 9.

Based on the statistical analysis results presented in Table 9, the following conclusions can be drawn:

1. Compared to BAGWO_A, which removes “The charisma and its update strategy”, BAGWO demonstrates significant performance improvements in the majority (over 50%) of the 24 benchmark functions. Although performance degradation is observed in some benchmark functions, this does not imply that the strategy is ineffective or unexplainable. Specifically, when BAGWO’s final charisma value $\:h$ is set relatively high, the gradual decrease in charisma value leads the algorithm to exhibit a stronger propensity for local search during later iterations. This characteristic, while enhancing local exploitation, may consequently impose certain limitations on the algorithm’s global exploration capabilities. It is noteworthy that these conclusions are based on a final charisma value of $\:h=0.99$, and adjusting the value of $\:h$ could optimize the strategy’s performance. Essentially, the BAGWO_A algorithm corresponds to the case where $\:h=0$.
2. Compared to BAGWO_B, which removes “Switching strategy of antennae length decay rate”, BAGWO shows significant performance enhancements in the vast majority of benchmark functions without any instances of performance degradation, fully demonstrating the effectiveness of this strategy.
3. Compared to BAGWO_C, which removes “The frequency of local exploitation update strategy”, BAGWO achieves notable performance improvements in nearly all tested benchmark functions, with no cases of performance deterioration observed. This further validates the universal effectiveness of the strategy.

**Table 9:** Wilcoxon rank-sum test statistical results for ablation experiments of BAGWO.

Compared algorithms BAGWO vs.	Different dimensions (+/=/-)			
	10	30	50	100
BAGWO_A	15/1/8	13/4/7	16/5/3	18/1/5
BAGWO_B	17/7/0	20/4/0	19/5/0	19/5/0
BAGWO_C	20/4/0	21/3/0	21/3/0	21/3/0

Based on the abovementioned analysis results, we have validated the effectiveness of the three improvement strategies in BAGWO. To further quantify the contribution of each strategy to the algorithm’s performance enhancement, this study conducted Friedman tests, with the results presented in Table 10. It is particularly noteworthy that the ranking of strategy contributions is inversely related to the statistical ranking in Table 10, and this ordering solely reflects the relative contribution levels of the three improvement strategies. The statistical analysis results demonstrate that in the performance improvement of BAGWO, “Switching strategy of antennae length decay rate” contributes most significantly, followed by “The frequency of local exploitation update strategy”, while “The charisma and its update strategy” shows relatively smaller contribution. The strategies validated in this study hold potential for enhancing the performance of other algorithms in future research.

**Table 10:** Friedman test results statistical results for ablation experiments of BAGWO.

Compared algorithms	Average rank under different dimensions				Average rank	Ranking
	10	30	50	100		
BAGWO	**1.713**	**1.572**	**1.458**	**1.401**	**1.536**	**1**
BAGWO_A	2.199	1.863	2.075	2.27	2.102	2
BAGWO_B	3.257	3.666	3.622	3.433	3.495	4
BAGWO_C	2.831	2.899	2.845	2.895	2.868	3

## BAGWO for classical engineering problems

In this section, the optimization effect of the proposed BAGWO in real-world engineering optimization problems are compared and verified. Optimization problems in the real world often come with various equality or inequality constraints, which significantly compress and partition the search space of feasible solutions for decision variables, greatly increasing the difficulty of optimization. The eight engineering problems selected in this section are typical representatives of such issues and are widely used as benchmark functions when evaluating and comparing the performance of various single-objective optimization algorithms. Therefore, this paper selects these eight engineering problems to comprehensively assess and test the performance of the BAGWO algorithm in handling real-world optimization problems. The algorithm demonstrates excellent overall optimization performance in these practical engineering optimization problems, indicating that it is likely to achieve good optimization results in other similar unknown engineering problems as well.

**Fig. 16:**
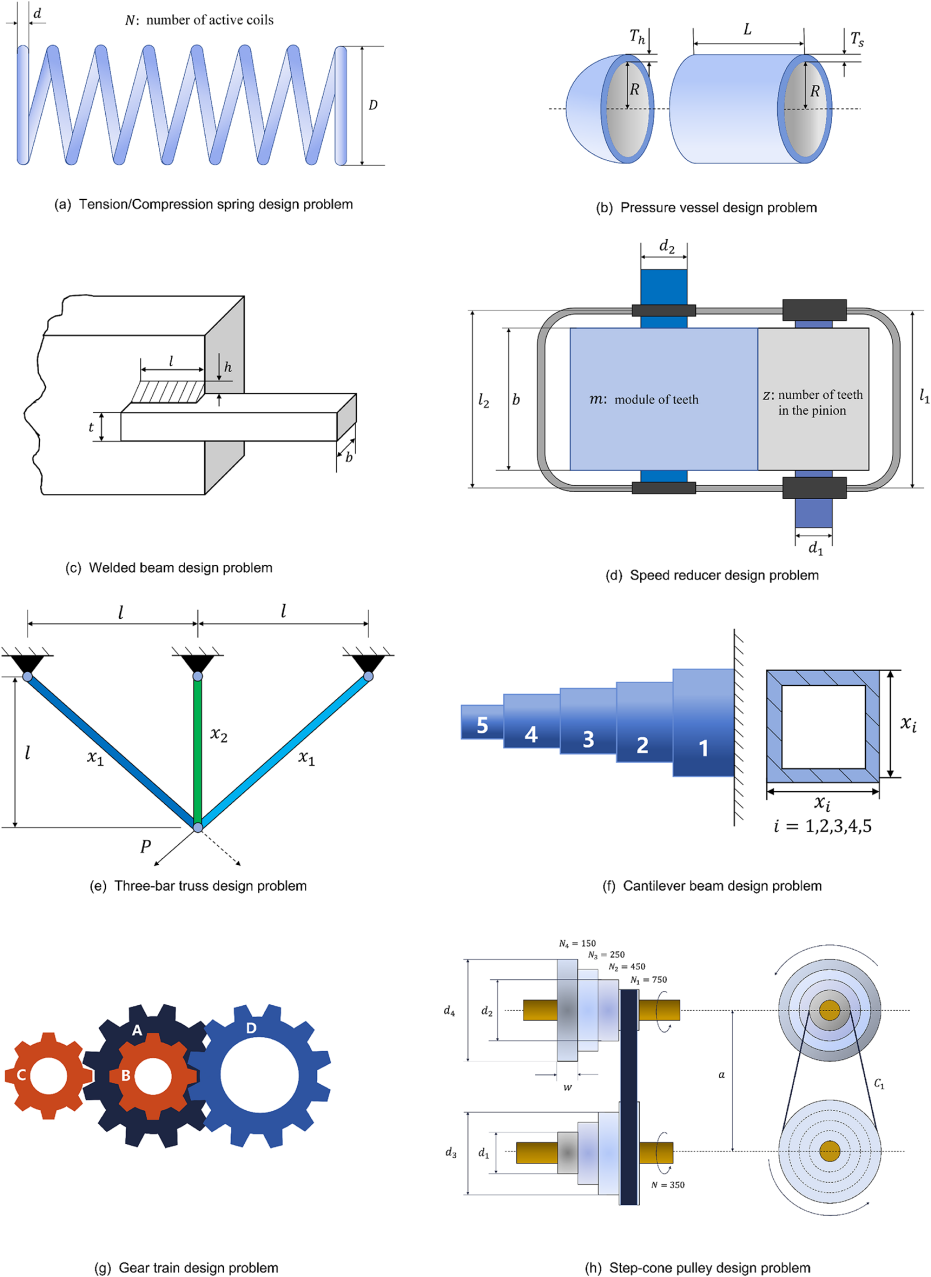
Eight engineering problems.

Eight engineering optimization problems commonly used in the literature are selected for this purpose. These cases include the Tension/Compression Spring Design problem (TCSD), Pressure Vessel Design problem (PVD), Welded Beam Design problem (WBD), Speed Reducer Design problem (SRD), Three-bar Truss Design problem (TTD), Cantilever Beam Design problem (CBD), Gear Train Design problem (GTD), Step-cone Pulley Design problem (SPD). The schematic diagrams of the eight engineering optimization cases are shown in Fig. 16, the characteristics of the eight engineering optimization problems mentioned are summarized as shown in Table 11. It can be observed that among the eight engineering problems, only the GTD problem is a discrete optimization problem, while the others are all continuous optimization problems. It should be noted that the minimum values corresponding to the $\:{f}_{\text{m}\text{i}\text{n}}$ column in Table 11 represent the best-known values. However, based on the needs of comparing different algorithms, we have used varying levels of numerical precision.

**Table 11:** A summary of the characteristics of eight engineering optimization problems.

Problems	Full Name	Dims	$\:{\varvec{n}}_{\mathbf{G}}$	$\:{\varvec{n}}_{\mathbf{H}}$	$\:{\varvec{f}}_{\mathbf{m}\mathbf{i}\mathbf{n}}$(Approximate value)	Opt. Type
TCSD	Tension/Compression spring design problem	3	4	0	0.012666	Continuous
PVD	Pressure vessel design problem	4	4	0	5884.39	Continuous
WBD	Welded beam design problem	4	7	0	1.692768	Continuous
SRD	Speed reducer design problem	7	11	0	2994.47	Continuous
TTD	Three-bar truss design problem	2	3	0	263.8915	Continuous
CBD	Cantilever beam design problem	5	1	0	1.339957	Continuous
GTD	Gear train design problem	4	1	1	0.0	Discrete
SPD	Step-cone pulley design problem	5	8	3	16.0856	Continuous

Note: In the first row of the table,$\:{n}_{\text{G}}$represents the number of inequality constraints,$\:{n}_{\text{H}}$represents the number of equality constraints.

All eight engineering optimization problems mentioned above are constrained optimization problems. When solving constrained optimization problems, it is necessary to address the constraints. According to research on constraint handling methods in evolutionary computation, constraint handling methods mainly include penalty function methods, feasibility rule methods, multi-objective methods, and so on. Among them, feasibility rule methods and multi-objective methods are primarily used for multi-objective constrained optimization problems. While they can also be applied to single-objective optimization, their handling is more complex and less frequently utilized. The penalty function method has a simple principle and is easy to implement; it can transform constrained optimization problems into unconstrained optimization problems, thereby simplifying the difficulty of solving the optimization problem. It is widely used for constraint handling in single-objective optimization problems. Therefore, the penalty function method is chosen as the constraint handling approach in this context.

The penalty function method involves adding a penalty function to the objective function, which transforms the constrained problem into an unconstrained one. Equation (24) is a common penalty function processing method. $\:{G}_{i}\left(\varvec{X}\right)$ is an inequality constraint, $\:{H}_{j}\left(\varvec{X}\right)$ is an equality constraint, $\:p$ is the number of inequality constraints, $\:q$ is the number of equality constraints, $\:{a}_{i}$ and $\:{b}_{j}$ are positive constants and penalty function coefficients, $\:m$ and $\:n$ are equal to 1 or 2. For the penalty function method shown in Eq. (24), when the candidate solution violates any constraint, the objective function value will increase, so that it is discarded in the optimization process. The setting of the penalty function coefficients $\:{a}_{i}$ and $\:{b}_{j}$ directly affects the final optimization results. If the values are set too low, the penalty strength is weak, making it easy for the algorithm to enter the infeasible region, which increases the risk of converging to an infeasible solution. Conversely, if the values are set too high, the algorithm may excessively avoid the infeasible areas, focusing only on satisfying the constraints while discarding potentially high-quality solutions. This can lead to getting trapped in local regions too early, reducing the global search capability and increasing the likelihood of missing the global optimal solution. Therefore, it is important to choose appropriate penalty coefficients. After referencing other related literature and conducting practical tests, this paper sets the penalty function coefficient $\:a$ and $\:b$ for the PVD problem at $\:{10}^{8}$. Since the GTD problem is a constraint-free problem, there is no need to set a coefficient. The coefficients for the remaining six problems are all set at $\:{10}^{6}$.

24 $$\:\begin{array}{c}\min F\left(\varvec{X}\right)=f\left(\varvec{X}\right)\pm\:\left(\sum\:_{i=1}^{p}{a}_{i}{G}_{i}\left(\varvec{X}\right)+\sum\:_{j=1}^{q}{b}_{j}{H}_{j}\left(\varvec{X}\right)\right)\end{array}$$

$$\:\text{s}.\text{t}.$$ $$\:\begin{array}{l}\:\:\:\:\:\:\:\:{G}_{i}\left(\varvec{X}\right)=\text{m}\text{a}\text{x}(0,{g}_{i}\left(\varvec{X}\right){)}^{m} \end{array} \\\:\begin{array}{l} \:{\:\:\:\:\:\:\:H}_{j}\left(\varvec{X}\right)=|{h}_{j}\left(\varvec{X}\right){|}^{n}\end{array}$$

In all the algorithms’ parameter settings related to the engineering optimization test, the maximum number of iterations for the algorithm is set to 500, and the number of search agents in the swarm is set to 30. The parameter settings for the all algorithms are detailed in Table 4. Similar to the evaluation of the optimization effectiveness of the benchmark functions in Sect. 4, in order to comprehensively evaluate the optimization effectiveness of the 15 optimization algorithms, including BAGWO, for the eight engineering problems, each optimization algorithm was run independently on each engineering optimization problem 30 times. The optimization effectiveness was then comprehensively evaluated using the mean, standard deviation, and statistical methods.

### Engineering optimization design case introduction

#### Tension/Compression spring design problem

In the TCSD problem, the optimization objective is to minimize the weight of the spring while satisfying constraints such as shear stress, deflection, and frequency limits. It is a classic optimization problem in mechanical engineering and has been widely used as a benchmark to evaluate the performance of optimization algorithms. The structure is schematically shown in Fig. 16(a). This problem contains three decision variables, wire diameter ($\:d$), mean coil diameter ($\:D$), and the number of active coils ($\:N$), and the mathematical description of this optimization problem is shown below.

**Consider**: $$\:\text{V}\text{a}\text{r}\text{i}\text{a}\text{b}\text{l}\text{e}\:\varvec{X}=\left[{x}_{1},{x}_{2,}{x}_{3}\right]=\left[d,D,N\right];$$

**Minimize**:

25 $$\:\begin{array}{c}f\left(\varvec{X}\right)=\left({x}_{3}+2\right){x}_{2}{x}_{1}^{2}\end{array}$$

**Subject to**:

26 $$\:\begin{array}{c}\begin{array}{c}{g}_{1}\left(\varvec{X}\right)=1-\frac{{x}_{3}{x}_{2}^{3}}{{71785x}_{1}^{4}}\le\:0\\\:\begin{array}{c}{g}_{2}\left(\varvec{X}\right)=\frac{4{x}_{2}^{2}-{x}_{1}{x}_{2}}{12566\left({x}_{2}{x}_{1}^{3}-{x}_{1}^{4}\right)}+\frac{1}{5108{x}_{1}}-1\le\:0\\\:{g}_{3}\left(\varvec{X}\right)=1-\frac{140.45{x}_{1}}{{x}_{2}^{2}{x}_{3}}\le\:0\\\:{g}_{4}\left(\varvec{X}\right)=\frac{{x}_{1}+{x}_{2}}{1.5}-1\le\:0\end{array}\end{array}\end{array}$$

**Variable range**: $$\:0.05\le\:{x}_{1}\le\:2;0.25\le\:{x}_{2}\le\:1.3;2\le\:{x}_{3}\le\:15;$$

#### Pressure vessel design problem

The PVD problem was first proposed by Kannan et al. in 1994^50^. Pressure vessels are widely used in industries such as chemical engineering, petroleum, natural gas, and energy. Optimizing their design can significantly reduce manufacturing costs, enhance safety, and minimize material waste. The optimization objective is to minimize the manufacturing cost of the pressure vessel while satisfying constraints such as pressure limits and geometric requirements. A schematic diagram of its structure is shown in Fig. 16(b). This problem contains four decision variables, thickness of the shell ($\:{T}_{s}$), thickness of the head ($\:{\text{T}}_{\text{h}}$), inner radius ($\:R$), and length of the cylindrical shape ($\:L$), and the mathematical description of this optimization problem is shown below.

**Consider**: $$\:\text{V}\text{a}\text{r}\text{i}\text{a}\text{b}\text{l}\text{e}\:\varvec{X}=\left[{x}_{1},{x}_{2,}{x}_{3,}{x}_{4}\right]=\left[{T}_{s},{T}_{h},R,L\right];$$

**Minimize**:

27 $$\:\begin{array}{c}f\left(\varvec{X}\right)=0.6224{x}_{1}{x}_{3}{x}_{4}+1.7781{x}_{2}{x}_{3}^{2}+3.1661{x}_{1}^{2}{x}_{4}+19.84{x}_{1}^{2}{x}_{3}\end{array}$$

**Subject to**:

28 $$\:\begin{array}{c}\begin{array}{c}{g}_{1}\left(\varvec{X}\right)=-{x}_{1}+0.0193{x}_{3}\le\:0\\\:\begin{array}{c}{g}_{2}\left(\varvec{X}\right)=-{x}_{2}+0.00954{x}_{3}\le\:0\\\:{g}_{3}\left(\varvec{X}\right)=-\pi\:{x}_{3}^{2}{x}_{4}-\frac{4\pi\:{x}_{3}^{3}}{3}+1,296,000\le\:0\\\:{g}_{4}\left(\varvec{X}\right)={x}_{4}-240\le\:0\end{array}\end{array}\end{array}$$

**Variable range**: $$\:0\le\:{x}_{i}\le\:99,i=\text{1,2};10\le\:{x}_{i}\le\:200,i=\text{3,4}$$

#### Welded beam design problem

The WBD problem is crucial in the design of welded beams, which are widely used in construction, bridge engineering, and heavy machinery manufacturing. Optimizing the design can enhance structural strength, reduce material costs, and simplify manufacturing processes. Due to its multi-constraint nature, this problem is extensively used to evaluate the optimization capabilities of algorithms. The objective of the WBD problem is to minimize the manufacturing cost of the welded beam while satisfying constraints such as bending stress, shear stress, and deflection limits. A schematic diagram of its structure is shown in Fig. 16(c). This problem contains four decision variables, thickness of weld ($\:h$), length of attached part of bar ($\:l$), the height of the bar ($\:t$), and thickness of the bar ($\:b$), constraints include shear stress ($\:\tau\:$), bending stress in the beam ($\:\sigma\:$), buckling load on the bar ($\:{P}_{c}$), end deflection of the beam ($\:{\updelta\:}$), and so on, and the mathematical description of this optimization problem is shown below.

**Consider**: $$\:\text{V}\text{a}\text{r}\text{i}\text{a}\text{b}\text{l}\text{e}\:\varvec{X}=\left[{x}_{1},{x}_{2,}{x}_{3,}{x}_{4}\right]=\left[h,l,t,b\right];$$

**Minimize**:

29 $$\:\begin{array}{c}f\left(\varvec{X}\right)=1.10471{x}_{1}^{2}{x}_{2}+0.04811{x}_{3}{x}_{4}\left(14.0+{x}_{2}\right)\end{array}$$

**Subject to**:

30 $$\:\begin{array}{c}\begin{array}{c}{g}_{1}\left(\varvec{X}\right)=\tau\:\left(\varvec{X}\right)-{\tau\:}_{max}\le\:0\\\:{g}_{2}\left(\varvec{X}\right)=\sigma\:\left(\varvec{X}\right)-{\sigma\:}_{max}\le\:0\\\:\begin{array}{c}{g}_{3}\left(\varvec{X}\right)=\delta\:\left(\varvec{X}\right)-{\delta\:}_{max}\le\:0\\\:{g}_{4}\left(\varvec{X}\right)={x}_{1}-{x}_{4}\le\:0\\\:\begin{array}{c}{g}_{5}\left(\varvec{X}\right)=P-{P}_{c}\left(\varvec{X}\right)\le\:0\\\:{g}_{6}\left(\varvec{X}\right)=0.125-{x}_{1}\le\:0\\\:{g}_{7}\left(\varvec{X}\right)=1.10471{x}_{1}^{2}{x}_{2}+0.04811{x}_{3}{x}_{4}\left(14.0+{x}_{2}\right)-5.0\le\:0\end{array}\end{array}\end{array}\end{array}$$

**Variable range**: $$\:0.1\le\:{x}_{i}\le\:2,i=\text{1,4};0.1\le\:{x}_{i}\le\:10,i=\text{2,3}$$

**Other variables**:

31 $$\:\begin{array}{c}\begin{array}{c}\tau\:\left(\varvec{X}\right)=\sqrt{{\left({\tau\:}^{{\prime\:}}\right)}^{2}+2{\tau\:}^{{\prime\:}}{\tau\:}^{{\prime\:}{\prime\:}}\frac{{x}_{2}}{2R}+{\left({\tau\:}^{{\prime\:}{\prime\:}}\right)}^{2}}\\\:{\tau\:}^{{\prime\:}}=\frac{p}{\sqrt{2{x}_{1}{x}_{2}}}\\\:\begin{array}{c}{\tau\:}^{{\prime\:}{\prime\:}}=\frac{MR}{J}\\\:M=P\left(L+\frac{{x}_{2}}{2}\right)\\\:\begin{array}{c}R=\sqrt{\frac{{x}_{2}^{2}}{4}+{\left(\frac{{x}_{1}+{x}_{2}}{2}\right)}^{2}}\\\:J=2\left[\sqrt{2}{x}_{1}{x}_{2}\left[\frac{{x}_{2}^{2}}{4}+{\left(\frac{{x}_{1}+{x}_{3}}{2}\right)}^{2}\right]\right]\\\:\begin{array}{c}\sigma\:\left(\varvec{X}\right)=\frac{6PL}{{x}_{3}^{2}{x}_{4}}\\\:\delta\:\left(\varvec{X}\right)=\frac{6P{L}^{3}}{{Ex}_{3}^{2}{x}_{4}}\\\:\begin{array}{c}{P}_{c}\left(\varvec{X}\right)=\frac{4.013E\sqrt{{x}_{3}^{2}{x}_{4}^{6}}}{{4 L}^{2}}\left(1-\frac{{x}_{3}}{2 L}\sqrt{\frac{E}{4G}}\right)\\\:P=6000\\\:\begin{array}{c}L=14\\\:{\delta\:}_{max}=0.25\\\:\begin{array}{c}E=30\times\:{10}^{6}\\\:G=12\times\:{10}^{6}\\\:\begin{array}{c}{\tau\:}_{max}=13,600\\\:{\sigma\:}_{max}=30,000\end{array}\end{array}\end{array}\end{array}\end{array}\end{array}\end{array}\end{array}\end{array}$$

#### Speed reducer design problem

The SRD problem is a complex multi-constrained optimization problem, widely used to evaluate the optimization capabilities of algorithms due to its numerous constraints. The speed reducer, as a core component of mechanical transmission systems, is extensively applied in industries such as automotive, aerospace, and industrial machinery. Optimizing its design can improve transmission efficiency, reduce noise, and minimize energy loss. The objective of the SRD problem is to minimize the weight of the speed reducer while satisfying constraints such as gear strength, shaft durability, and geometric requirements^51^. A schematic diagram of its structure is shown in Fig. 16(d). This problem contains seven decision variables, face width ($\:b$), module of teeth ($\:m$), number of teeth in the pinion ($\:z$), length of the first shaft between bearings ($\:{l}_{1}$), length of the second shaft between bearings ($\:{l}_{2}$), the diameter of the first shafts ($\:{d}_{1}$) and the diameter of second shafts ($\:{d}_{2}$), and the mathematical description of this optimization problem is shown below.

**Consider**: $$\:\text{V}\text{a}\text{r}\text{i}\text{a}\text{b}\text{l}\text{e}\:\varvec{X}=\left[{x}_{1}{,x}_{2},{x}_{3},{x}_{4}{,x}_{5},{x}_{6},{x}_{7}\right]=\left[b,m,z,{l}_{1},{l}_{2},{d}_{1},{d}_{2}\right];$$

**Minimize**:

32 $$\:\begin{array}{c}f\left(\varvec{X}\right)=0.7854{x}_{1}{x}_{2}^{2}\left(3.3333{x}_{3}^{2}+14.9334{x}_{3}-43.0934\right)-1.508{x}_{1}\left({x}_{6}^{2}+{x}_{7}^{2}\right)+7.4777\left({x}_{6}^{3}+{x}_{7}^{3}\right)+0.7854\left({x}_{4}{x}_{6}^{2}+{x}_{5}{x}_{7}^{2}\right)\end{array}$$

**Subject to**:

33 $$\:\begin{array}{c}\begin{array}{c}{g}_{1}\left(\varvec{X}\right)=\frac{27}{{x}_{1}{x}_{2}^{2}{x}_{3}}-1\le\:0\\\:{g}_{2}\left(\varvec{X}\right)=\frac{397.5}{{x}_{1}{x}_{2}^{2}{x}_{3}^{2}}-1\le\:0\\\:\begin{array}{c}{g}_{3}\left(\varvec{X}\right)=\frac{1.9{x}_{4}^{3}}{{x}_{2}{x}_{6}^{4}{x}_{3}}-1\le\:0\\\:{g}_{4}\left(\varvec{X}\right)=\frac{1.93{x}_{3}^{4}}{{x}_{2}{x}_{7}^{4}{x}_{3}}-1\le\:0\\\:\begin{array}{c}{g}_{5}\left(\varvec{X}\right)=\frac{\left[\right(745\left({x}_{4}/{x}_{2}{x}_{3}\right){)}^{2}+16.9\times\:{10}^{6}{]}^{\frac{1}{2}}}{110{x}_{6}^{3}}-1\le\:0\\\:{g}_{6}\left(\varvec{X}\right)=\frac{\left[\right(745\left({x}_{5}/{x}_{2}{x}_{3}\right){)}^{2}+157.5\times\:{10}^{6}{]}^{\frac{1}{2}}}{85{x}_{7}^{3}}-1\le\:0\\\:\begin{array}{c}{g}_{7}\left(\varvec{X}\right)=\frac{{x}_{2}{x}_{3}}{40}-1\le\:0\\\:{g}_{8}\left(\varvec{X}\right)=\frac{{5x}_{2}}{{x}_{1}}-1\le\:0\\\:\begin{array}{c}{g}_{9}\left(\varvec{X}\right)=\frac{{x}_{1}}{12{x}_{2}}-1\le\:0\\\:{g}_{10}\left(\varvec{X}\right)=\frac{{1.5x}_{6}+1.9}{{x}_{4}}-1\le\:0\\\:{g}_{11}\left(\varvec{X}\right)=\frac{{1.1x}_{7}+1.9}{{x}_{5}}-1\le\:0\end{array}\end{array}\end{array}\end{array}\end{array}\end{array}$$

**Variable rang**: $$\:2.6\le\:{x}_{1}\le\:3.6,\:\:0.7\le\:{x}_{2}\le\:0.8,\:17\le\:{x}_{3}\le\:28,\:\:7.3\le\:{x}_{4}\le\:8.3,\:\:7.3\le\:{x}_{5}\le\:\:8.3,\:\:2.9\le\:{x}_{6}\le\:3.9\:,\:\:5.0\le\:{x}_{7}\le\:5.5$$

#### Three-bar truss design problem

The TTD problem is a classic structural optimization problem, with the objective of minimizing the weight of the truss structure while satisfying constraints such as stress limits, displacement limits, and geometric requirements^52^. The three-bar truss structure plays a significant role in civil engineering and architectural applications, including bridges, towers, and roof structures. Optimizing its design can enhance structural stability, reduce material consumption, and lower construction costs. A schematic diagram of its structure is illustrated in Fig. 16(e). This problem contains two decision variables, edge rod length ($\:{A}_{1}$), central rod length ($\:{A}_{2}$), and the mathematical description of this optimization problem is shown below.

**Consider**: $$\:\text{V}\text{a}\text{r}\text{i}\text{a}\text{b}\text{l}\text{e}\:\varvec{X}=\left[{x}_{1},{x}_{2}\right]=[{A}_{1},{A}_{2}];$$

**Minimize**:

34 $$\:\begin{array}{c}f\left(\varvec{X}\right)=\left(2\sqrt{2}{x}_{1}+{x}_{2}\right)l\end{array}$$

**Subject to**:

35 $$\:\begin{array}{c}\begin{array}{c}{g}_{1}\left(\varvec{X}\right)=\frac{\sqrt{2}{x}_{1}+{x}_{2}}{\sqrt{2}{x}_{1}^{2}+{2{x}_{1}x}_{2}}P-\sigma\:\le\:0\\\:{g}_{2}\left(\varvec{X}\right)=\frac{{x}_{2}}{\sqrt{2}{x}_{1}^{2}+{2{x}_{1}x}_{2}}P-\sigma\:\le\:0\\\:{g}_{3}\left(\varvec{X}\right)=\frac{1}{\sqrt{2}{x}_{1}^{2}+{2{x}_{1}x}_{2}}P-\sigma\:\le\:0\end{array}\end{array}$$

**Variable range**: $$\:0\le\:{x}_{i}\le\:99,i=\text{1,2};$$

**Other variables**: $$\:l=100;P=2;\sigma\:=2;$$

#### Cantilever beam design problem

The CBD problem is a classic engineering optimization problem, with the objective of minimizing the weight of the cantilever beam while satisfying constraints such as stress limits, deflection limits, and geometric requirements^42^. Cantilever beams are widely utilized in fields such as architecture, mechanical engineering, and aerospace, with applications including aircraft wings, bridges, and robotic arms. Optimizing their design can enhance structural strength, reduce material consumption, and lower manufacturing costs. A schematic diagram of the CBD structure is shown in Fig. 16(f). This problem contains five decision variables corresponding to the side lengths of the different arm beams, the cantilever side lengths from the fixed end to the cantilever end are denoted by $\:{x}_{1},{x}_{2},{x}_{3},{x}_{4},{x}_{5}$ respectively, and the mathematical description of this optimization problem is shown below.

**Consider**: $$\:\text{V}\text{a}\text{r}\text{i}\text{a}\text{b}\text{l}\text{e}\:\varvec{X}=\left[{x}_{1},{x}_{2},{x}_{3},{x}_{4},{x}_{5}\right];$$

**Minimize**:

36 $$\:\begin{array}{c}f\left(\varvec{X}\right)=0.0624\left({x}_{1}+{x}_{2}+{x}_{3}+{x}_{4}+{x}_{5}\right)\end{array}$$

**Subject to**:

37 $$\:\begin{array}{c}g\left(\varvec{X}\right)=\frac{61}{{x}_{1}^{3}}+\frac{27}{{x}_{2}^{3}}+\frac{19}{{x}_{3}^{3}}+\frac{7}{{x}_{4}^{3}}+\frac{1}{{x}_{5}^{3}}-1\le\:0\end{array}$$

**Variable range**: $$\:0.01\le\:{x}_{i}\le\:100,i=\text{1,2},\text{3,4},5;$$

#### Gear train design problem

The GTD problem is a classic discrete optimization problem, aiming to minimize the gear ratio error in gear transmission systems^53^. Gear transmission systems play a crucial role in industries such as automotive, mechanical manufacturing, and energy. Optimizing their design can improve transmission efficiency, reduce noise, and extend service life. The objective of the gear transmission design problem is to determine the optimal number of gear teeth to achieve the desired gear ratio while satisfying the constraint that the number of gear teeth must be a positive integer, as illustrated in Fig. 16(g). This problem contains four decision variables, gear A tooth count ($\:{T}_{a}$), gear B tooth count ($\:{T}_{b}$), gear C tooth count ($\:{T}_{c}$), gear D tooth count ($\:{T}_{d}$), and the mathematical description of this optimization problem is shown below.

**Consider**: $$\:\text{V}\text{a}\text{r}\text{i}\text{a}\text{b}\text{l}\text{e}\:\varvec{X}=\left[{x}_{1},{x}_{2},{x}_{3},{x}_{4}\right]=\left[{T}_{a},{T}_{b},{T}_{c},{T}_{d}\right];$$

**Minimize**:

38 $$\:\begin{array}{c}f\left(\varvec{X}\right)={\left(\frac{1}{6.931}-\frac{{x}_{2}{x}_{3}}{{x}_{1}{x}_{4}}\right)}^{2}\end{array}$$

**Subject to**: $$\:{x}_{i}\in\:{N}_{+},i=\text{1,2},\text{3,4};$$

**Variable range**: $$\:12\le\:{x}_{i}\le\:60,i=\text{1,2},\text{3,4};$$

#### Step-cone pulley design problem

The SPDproblem is a complex engineering optimization problem involving multiple equality and inequality constraints. Its objective is to minimize the weight of the step-cone pulley while satisfying constraints related to transmission ratio, geometry, and strength^1,54^. Step-cone pulleys play a vital role in mechanical transmission systems, transferring power between shafts via a belt mechanism. They are widely used in applications such as machine tools, textile machinery, and conveyor equipment. Optimizing their design can enhance transmission efficiency, reduce energy loss, and lower manufacturing costs. A schematic diagram of the system is shown in Fig. 16(h). This problem contains five decision variables, pulley width ($\:w$), diameters of the four stepped pulleys ($\:{d}_{1},{d}_{2},{d}_{3},{d}_{4}$), and the mathematical description of this optimization problem is shown below.

**Consider**: $$\:\text{V}\text{a}\text{r}\text{i}\text{a}\text{b}\text{l}\text{e}\:\varvec{X}=\left[{x}_{1},{x}_{2},{x}_{3},{x}_{4},{x}_{5}\right]=\left[{w,d}_{1},{d}_{2},{d}_{3},{d}_{4}\right];$$

**Minimize**:

39 $$\:\begin{array}{c}f\left(\varvec{X}\right)=\rho\:w\left[{d}_{1}^{2}\left[11+{\left(\frac{{N}_{1}}{N}\right)}^{2}\right]+{d}_{2}^{2}\left[1+{\left(\frac{{N}_{2}}{N}\right)}^{2}\right]+{d}_{3}^{2}\left[1+{\left(\frac{{N}_{3}}{N}\right)}^{2}\right]+{d}_{4}^{2}\left[1+{\left(\frac{{N}_{4}}{N}\right)}^{2}\right]\right]\end{array}$$

**Subject to**:

40 $$\:\begin{array}{c}\begin{array}{c}{h}_{1}\left(\varvec{X}\right)={C}_{1}-{C}_{2}=0\\\:{h}_{2}\left(\varvec{X}\right)={C}_{1}-{C}_{3}=0\\\:\begin{array}{c}{h}_{3}\left(\varvec{X}\right)={C}_{1}-{C}_{4}=0\\\:{g}_{i=\text{1,2},\text{3,4}}\left(\varvec{X}\right)=-{R}_{i}\le\:2\\\:{g}_{i=\text{5,6},\text{7,8}}\left(\varvec{X}\right)=\left(0.75\times\:745.6998\right)-{P}_{i-1}\le\:0\end{array}\end{array}\end{array}$$

**Variable range**: $$\:0\le\:{x}_{i}\le\:60,i=\text{1,2};0\le\:{x}_{i}\le\:90,i=\text{3,4},5$$

**Other variables**:

41 $$\:\begin{array}{c}\begin{array}{c}{C}_{i}=\frac{\pi\:{d}_{i}}{2}\left(1+\frac{{N}_{i}}{N}\right)+\frac{{\left(\frac{{N}_{i}}{N}-1\right)}^{2}}{4a}+2a,i=\text{1,2},\text{3,4}\\\:{R}_{i}=\text{exp}\left(\mu\:\left[\pi\:-2{sin}^{-1}\left[\left(\frac{{N}_{i}}{N}-1\right)\frac{{d}_{i}}{2a}\right]\right]\right),i=\text{1,2},\text{3,4}\\\:\begin{array}{c}{P}_{i}=stw\left(1-{R}_{i}\right)\frac{\pi\:{d}_{i}{N}_{i}}{60},i=\text{1,2},\text{3,4}\\\:t=8\\\:\begin{array}{c}s=1.75\\\:\mu\:=0.35\\\:\begin{array}{c}\rho\:=7200\\\:a=3\end{array}\end{array}\end{array}\end{array}\end{array}$$

### Comparison of calculation results for engineering problems

**Fig. 17:**
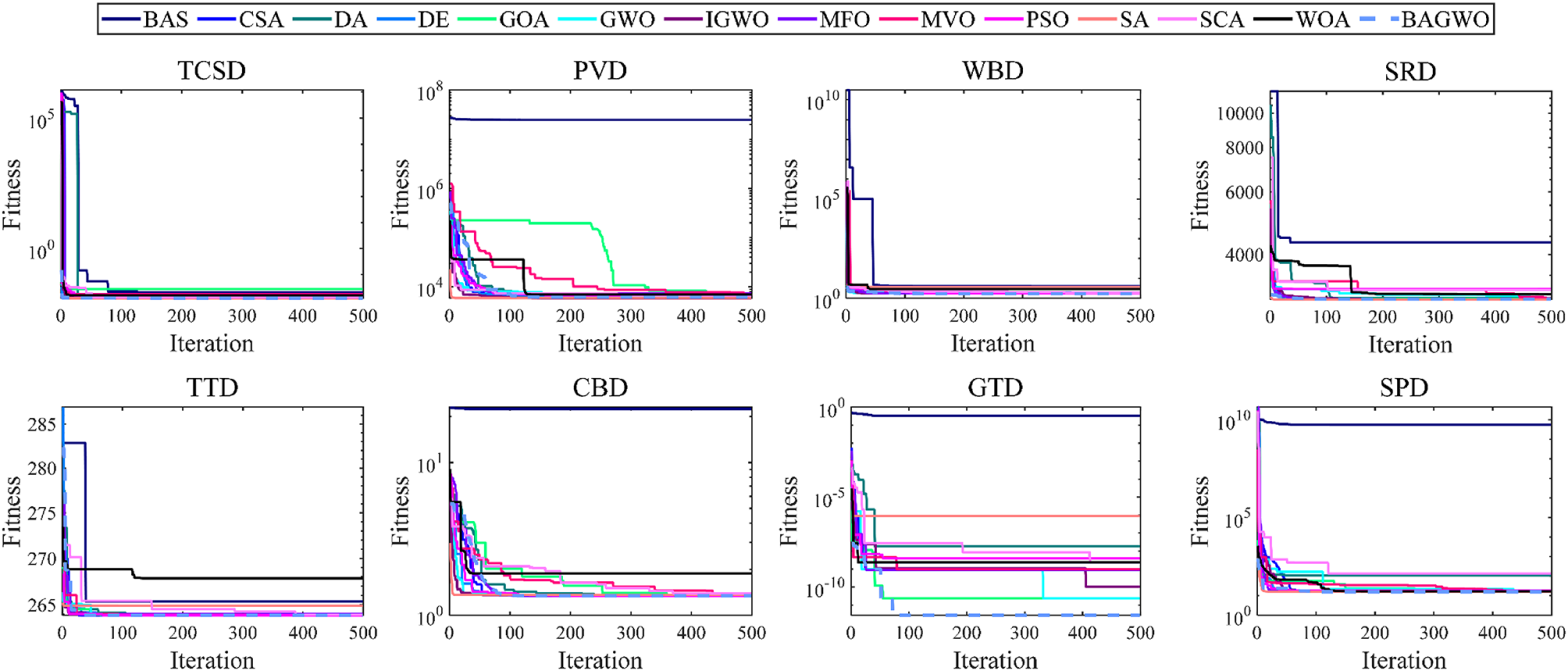
Convergence plots of eight engineering problems.

**Fig. 18:**
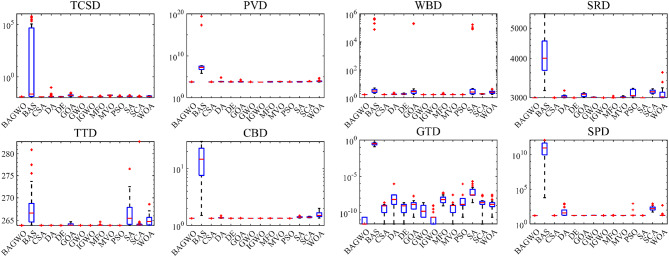
Boxplot of results for eight engineering problems.

The iterative process results for the engineering problems are illustrated in Fig. 17. As shown, BAGWO demonstrates good optimization performance across all problems, exhibiting relatively faster convergence rates compared to other benchmark algorithms while consistently ranking among the top performers in terms of final optimization results. The corresponding box plots in Fig. 18, which characterize the accuracy and stability of the computational results, reveal that BAGWO maintains excellent stability across all eight engineering problems without any noticeable performance degradation. This section primarily focuses on the qualitative analysis of the engineering problems. For a comprehensive quantitative analysis, please refer to Sect. 5.3, with detailed numerical data available in **Table A.6** of the supplementary materials. It should be noted that due to the poor performance of the GA on these eight engineering problems, the data curves of different algorithms were compressed into a very small region, making it difficult to discern meaningful information. Therefore, the data for the GA algorithm has been removed from Figs. 17 and 18. This is also clearly demonstrated in the data analysis of Sect. 5.3, where the overall performance of the GA algorithm is the worst.

### Engineering optimization problems statistical analysis results

In this subsection, the optimization results of eight engineering problems are statistically analyzed and processed. **Table A.6** in the Supplementary Material presents the mean and variance of the optimization results for the 14 algorithms across the eight engineering problems. It is observed that BAGWO exhibits the best optimization effect for four of the engineering problems, and its performance for the remaining four problems is also commendable. However, as mentioned in Sect. 4, the mean and standard deviation alone cannot provide a definitive comprehensive ranking of the optimization effectiveness of an algorithm when dealing with different optimization problem outcomes. Consequently, in this subsection, the Wilcoxon rank-sum test and Friedman test from the nonparametric statistical analysis method are also employed for a comprehensive comparison and analysis of the optimization performance.

#### Wilcoxon rank-sum test

The Wilcoxon rank-sum test is utilized to determine if there is a significant difference between the medians of two related samples. It is employed to assess the performance of the proposed algorithm against other algorithms. The test is conducted at a significance level of 5%. In the Wilcoxon rank-sum test statistical results, the symbol “+” indicates that BAGWO has better optimization performance than the algorithm being compared, the symbol “=” indicates that BAGWO has comparable optimization performance to the algorithm being compared, and the symbol “-” indicates that BAGWO has worse optimization performance than the algorithm being compared. In Table 12, the optimization performance comparison results of BAGWO are presented in comparison with other algorithms. From the statistical analysis data in the table, it is observed that the optimization performance of BAGWO is significantly superior to that of other algorithms involved in the comparison.

**Table 12:** Wilcoxon rank-sum test statistical results for eight engineering problems.

Compared Algorithms BAGWO vs.	better/comparable/worse (+/=/-)
BAS	8/0/0
CSA	4/1/3
DA	8/0/0
DE	6/1/1
GA	8/0/0
GOA	8/0/0
GWO	7/0/1
IGWO	5/0/3
MFO	6/1/1
MVO	8/0/0
PSO	6/1/1
SA	6/1/1
SCA	8/0/0
WOA	8/0/0
**Overall (+/=/-)**	**96/5/11**
**Dominant ratio**	**85.71%**

#### Friedman test

The Friedman test is used to determine if the overall distribution, represented by more than two groups of samples, is the same. It is also used to test for differences between the test results of the proposed BAGWO and other algorithms. The algorithm’s optimization performance is ranked on average, and the test is conducted at a 5% significance level. Table 13 presents the optimization performance comparison results of BAGWO relative to other algorithms. From the statistical analysis data, it can be seen that the comprehensive optimization performance ranking of BAGWO ranks first among the algorithms involved in the comparison.

The results of the Friedman test and Wilcoxon rank-sum test quantitatively demonstrate the superior comprehensive optimization performance of the proposed BAGWO in solving real-world engineering problems. Similarly, its effectiveness and outstanding optimization results in addressing constrained optimization problems are also confirmed. However, it should be noted that the real world presents a vast array of constrained optimization problems, and the constrained problems discussed in this chapter cannot represent all such cases. Therefore, the performance of BAGWO still needs to be tested and validated on a broader range of constrained optimization problems in the future. Nevertheless, based on the experimental data and analysis presented in this study, BAGWO has proven to be a highly competitive optimization algorithm.

**Table 13:** Friedman test results for engineering problems.

Compared Algorithms	Mean rank	Rank
BAGWO	2.917	1
IGWO	3.431	2
CSA	3.529	3
DE	5.615	4
GWO	6.088	5
PSO	6.3	6
MFO	7.002	7
MVO	7.965	8
GOA	9.031	9
DA	9.313	10
SA	9.333	11
WOA	10.256	12
SCA	10.696	13
BAS	13.525	14
GA	15	15

## Conclusion and future work

This paper proposes a novel hybrid optimization algorithm, BAGWO, which integrates and enhances the GWO and BAS. Three improvement strategies are introduced to boost its overall optimization performance, and ablation experiments confirm their effectiveness. Comprehensive evaluations on the CEC2005 and CEC2017 benchmark functions, along with eight real-world engineering problems, demonstrate that BAGWO outperforms other advanced algorithms in accuracy, stability, and convergence speed. It exhibits strong competitiveness in global optimization tasks and maintains robust performance across problems of varying dimensions, with relatively slow performance degradation, highlighting its adaptability to diverse optimization challenges.

While the CEC benchmark functions and engineering problems provide a fair evaluation framework, their scope remains limited and does not encompass all types of single-objective optimization problems. Despite this, due to the representativeness of the CEC benchmark functions and engineering optimization problems selected in this paper, the experimental data and statistical analysis presented in this paper still clearly establish BAGWO as a highly competitive algorithm, offering significant advantages in accuracy and stability for global optimization and providing an effective solution for real-world applications. It is worth noting that BAGWO’s competitive optimization performance comes at the cost of longer runtimes. Although reducing the frequency of local exploitation can alleviate this issue, it may lead to performance degradation in certain scenarios. As a result, BAGWO is particularly well-suited for optimization tasks where runtime sensitivity is not a critical constraint.

Overall, thanks to its competitive optimization performance, BAGWO has already been successfully applied to multi-design point optimization for variable cycle engines and parameter estimation under partial performance data. Making it a powerful mathematical tool for solving practical engineering challenges. In the future, BAGWO can be extended to other engineering optimization domains, such as robotics, unmanned aerial vehicles, and mechanical design optimization, further broadening its applicability and impact.

## Electronic supplementary material

Below is the link to the electronic supplementary material.

Supplementary Material 1

## Data Availability

Data is provided within the manuscript or supplementary information files.

## Abbreviations

ABC: Artificial Bee Colony
AFSA: Artificial Fish Swarm Algorithm
ALDR: Antennae Length Decay Rate
ANN: Artificial Neural Network
BAGWO: Beetle Antennae search and Grey Wolf Optimizer hybrid algorithm
BAS: Beetle Antennae Search algorithm
CBD: Cantilever Beam Design problem
CEC: Congress on Evolutionary Computation
CRO: Chemical Reaction Optimization
CSA: Chameleon Swarm Algorithm
DA: Dragonfly Algorithm
DE: Differential Evolution
ES: Evolution Strategy
FA: Firefly Algorithm
FECO: Five-Elements Cycle Optimization
GA: Genetic Algorithm
GOA: Grasshopper Optimization Algorithm
GSA: Gravitational Search Algorithm
GTD: Gear Train Design problem
GWO: Grey Wolf Optimizer
HBSA: Historically Best Search Agent
IGWO: Improved Grey Wolf Optimizer
IA: Immune Algorithm
LHS: Latin Hypercube Sampling
LSO: Light Spectrum Optimizer
MFO: Moth-Flame Optimization algorithm
MVO: Multi-Verse Optimizer
NFL: No Free Lunch
PSO: Particle Swarm Optimization
PVD: Pressure Vessel Design problem
SA: Simulated Annealing
SCA: Sine-Cosine Algorithm
SPD: Step-Cone Pulley Design problem
SRD: Speed Reducer Design problem
SSA: Sparrow Search Algorithm
TCSD: Tension/Compression Spring Design problem
TLBO: Teaching-Learning-Based Optimization
TTD: Three-Bar Truss Design problem
WBD: Welded Beam Design problem
WCA: Water Cycle Algorithm
WOA: Whale Optimization Algorithm
YYPO: Yin-Yang-Pair Optimization
