# Supplementary material for: Multiple strategy enhanced hybrid algorithm BAGWO combining beetle antennae search and grey wolf optimizer for global optimization
Source: Sci Rep. 2025 May 2;15:15460. doi: 10.1038/s41598-025-98816-0 (PMC12048570; doi:10.1038/s41598-025-98816-0)
Supplement: Supplementary file 1 — Supplementary Material 1 [file 41598_2025_98816_MOESM1_ESM.pdf]

Appendix A. Supplementary Material

The raw data from **Section 4** and **Section 5** are included in the Supplementary Material as follows:

Table A.1 Results and comparison of different algorithms on 5 benchmark functions with fixed dim (F8-F12)

Algorithms	Index	Functions					Dominant quantity
		F8	F9	F10	F11	F12	
BAGWO	Mean.	**9.98E-1**	-1.03E+0	-3.86E+0	**-1.02E+1**	**-1.05E+1**	**3**
	Std	**4.12E-17**	6.58E-16	2.68E-15	**6.45E-15**	**2.33E-15**	
BAS	Mean.	2.77E+2	-1.03E+0	-3.86E+0	-5.04E+0	-6.58E+0	0
	Std	2.41E+2	5.77E-6	1.69E-2	2.56E+0	3.14E+0	
CSA	Mean.	1.13E+0	-1.03E+0	-3.86E+0	-8.47E+0	-9.91E+0	0
	Std	4.31E-1	6.32E-16	2.57E-15	2.42E+0	1.96E+0	
DA	Mean.	1.23E+0	-1.03E+0	-3.86E+0	-7.61E+0	-7.58E+0	0
	Std	6.73E-1	2.52E-7	1.22E-3	2.82E+0	3.09E+0	
DE	Mean.	9.98E-1	-1.03E+0	-3.86E+0	-1.01E+1	-1.05E+1	0
	Std	0.00E+0	6.78E-16	2.71E-15	3.06E-1	1.11E-1	
GA	Mean.	1.21E+0	-9.54E-1	-3.23E+0	-1.19E+0	-1.30E+0	0
	Std	7.94E-1	9.92E-2	4.49E-1	7.42E-1	1.22E+0	
GOA	Mean.	1.03E+0	-1.03E+0	-3.81E+0	-5.05E+0	-5.74E+0	0
	Std	1.81E-1	3.17E-13	1.96E-1	3.04E+0	3.80E+0	
GWO	Mean.	4.23E+0	-1.03E+0	-3.86E+0	-9.81E+0	-1.00E+1	0
	Std	4.25E+0	2.91E-8	1.57E-3	1.28E+0	1.90E+0	
IGWO	Mean.	9.98E-1	-1.03E+0	**-3.86E+0**	-9.84E+0	-1.05E+1	**1**
	Std	1.93E-16	6.39E-16	**2.54E-15**	1.04E+0	1.03E-7	
MFO	Mean.	2.74E+0	-1.03E+0	-3.86E+0	-5.98E+0	-7.85E+0	0
	Std	2.58E+0	6.78E-16	2.71E-15	3.55E+0	3.62E+0	
MVO	Mean.	9.98E-1	-1.03E+0	-3.86E+0	-8.14E+0	-8.58E+0	0
	Std	2.49E-11	5.05E-7	3.44E-6	2.98E+0	3.12E+0	
PSO	Mean.	3.82E+0	**-1.03E+0**	-3.86E+0	-6.81E+0	-9.05E+0	**1**
	Std	2.79E+0	**4.79E-16**	2.73E-3	3.50E+0	2.80E+0	
SA	Mean.	1.06E+0	-1.03E+0	-3.84E+0	-5.62E+0	-4.89E+0	0
	Std	3.62E-1	3.78E-11	1.41E-1	3.18E+0	3.10E+0	
SCA	Mean.	1.40E+0	-1.03E+0	-3.86E+0	-1.97E+0	-3.77E+0	0
	Std	8.06E-1	3.67E-5	2.84E-3	1.52E+0	1.71E+0	
WOA	Mean.	2.31E+0	-1.03E+0	-3.85E+0	-7.00E+0	-6.68E+0	0
	Std	2.47E+0	1.49E-9	1.06E-2	2.63E+0	3.36E+0	

Table A.2 Results and comparison of different algorithms on 19 benchmark functions with dim 10 (F1-F7, F13-F24)

Functions	Index	BAGWO	BAS	CSA	DA	DE	GA	GOA	GWO	IGWO	MFO	MVO	PSO	SA	SCA	WOA
F1	Mean.	1.34E-32	2.99E+4	2.14E-8	1.04E+1	2.11E-19	8.59E+1	4.26E-4	6.65E-57	1.46E-60	2.80E-13	1.45E-2	5.58E-5	3.91E-16	1.78E-12	**9.97E-77**
	Std	4.20E-33	2.90E+4	5.72E-8	1.52E+1	1.82E-19	3.63E+2	1.95E-3	1.70E-56	4.92E-60	8.14E-13	5.04E-3	7.41E-5	1.40E-15	4.02E-12	**5.43E-76**
F2	Mean.	**3.91E-32**	1.11E+6	5.35E-1	4.99E+2	3.47E+1	3.48E+3	1.55E+0	1.28E-24	2.58E-26	6.67E+2	1.34E-1	7.60E-2	3.75E+1	1.71E-3	1.69E+2
	Std	**2.35E-32**	1.07E+6	6.14E-1	1.30E+3	2.26E+1	1.44E+3	2.51E+0	4.54E-24	1.40E-25	1.73E+3	1.26E-1	5.97E-2	4.69E+1	4.04E-3	3.23E+2
F3	Mean.	**1.40E-32**	2.53E+4	1.76E-8	1.00E+1	1.85E-19	2.91E+1	7.60E-5	1.67E-2	1.18E-9	2.89E-13	1.42E-2	4.06E-5	2.66E-17	4.46E-1	7.28E-3
	Std	**5.29E-33**	2.27E+4	4.90E-8	1.70E+1	1.86E-19	5.22E+1	1.81E-4	6.36E-2	7.75E-10	4.22E-13	5.75E-3	4.90E-5	1.05E-16	1.38E-1	3.54E-2
F4	Mean.	**1.04E+3**	6.58E+10	1.48E+3	1.71E+8	7.75E+3	2.88E+8	4.35E+3	4.57E+7	5.42E+4	1.30E+8	1.79E+4	2.62E+3	7.27E+3	9.58E+8	9.19E+7
	Std	**1.16E+3**	3.39E+10	2.11E+3	4.15E+8	9.12E+3	4.13E+8	4.06E+3	1.09E+8	2.34E+4	4.50E+8	8.34E+3	2.86E+3	4.68E+3	4.12E+8	1.26E+8
F5	Mean.	-3.37E+3	-6.47E+1	-2.89E+3	-2.82E+3	**-4.16E+3**	-1.31E+3	-2.68E+3	-2.71E+3	-4.02E+3	-3.26E+3	-2.96E+3	-2.49E+3	-4.15E+3	-2.13E+3	-3.06E+3
	Std	1.55E+2	1.98E+3	8.14E+2	3.52E+2	**5.10E+1**	2.50E+2	3.83E+2	3.87E+2	1.32E+2	4.25E+2	2.54E+2	4.07E+2	6.33E+1	1.65E+2	5.78E+2
F6	Mean.	4.71E-15	1.75E+1	1.22E+1	3.12E+0	2.38E-10	1.41E+1	9.75E-1	7.67E-15	5.18E-15	3.85E-2	4.76E-1	6.74E-3	8.84E-9	2.34E-6	**4.12E-15**
	Std	1.45E-15	3.81E+0	9.00E+0	1.24E+0	1.36E-10	4.72E+0	9.56E-1	2.18E-15	1.70E-15	2.11E-1	7.51E-1	4.06E-3	3.78E-8	7.40E-6	**2.38E-15**
F7	Mean.	**4.71E-32**	2.62E+8	1.43E+0	4.72E+0	3.41E-20	1.30E+0	1.70E+0	4.28E-3	2.15E-10	1.75E+0	2.16E-2	1.39E-6	1.04E-2	9.69E-2	8.66E-3
	Std	**1.67E-47**	5.53E+8	1.80E+0	5.69E+0	7.45E-20	1.69E+0	2.34E+0	8.04E-3	1.19E-10	9.32E+0	7.98E-2	1.82E-6	5.68E-2	3.88E-2	1.34E-2
F13	Mean.	**4.02E+2**	3.17E+4	4.08E+2	4.53E+2	4.07E+2	5.48E+2	4.10E+2	4.17E+2	4.04E+2	4.22E+2	4.05E+2	4.05E+2	4.21E+2	4.62E+2	4.63E+2
	Std	**7.58E-1**	2.53E+4	1.20E+1	5.86E+1	5.81E-1	1.14E+2	1.62E+1	1.95E+1	1.00E+0	2.94E+1	1.36E+0	2.84E+0	2.96E+1	1.89E+1	5.83E+1
F14	Mean.	6.00E+2	7.35E+2	6.07E+2	6.26E+2	**6.00E+2**	6.57E+2	6.13E+2	6.01E+2	6.00E+2	6.05E+2	6.03E+2	6.11E+2	6.00E+2	6.23E+2	6.41E+2
	Std	3.88E-5	6.04E+1	3.36E+0	1.48E+1	**3.74E-7**	1.41E+1	1.45E+1	1.26E+0	3.99E-2	7.60E+0	3.01E+0	1.01E+1	2.44E-3	5.19E+0	1.33E+1
F15	Mean.	**8.05E+2**	1.01E+3	8.13E+2	8.45E+2	8.18E+2	8.77E+2	8.27E+2	8.17E+2	8.10E+2	8.32E+2	8.23E+2	8.22E+2	8.25E+2	8.47E+2	8.45E+2
	Std	**2.04E+0**	1.13E+2	6.29E+0	1.60E+1	3.01E+0	1.48E+1	1.26E+1	8.22E+0	7.09E+0	1.28E+1	9.73E+0	8.59E+0	7.55E+0	7.16E+0	1.73E+1
F16	Mean.	**1.10E+3**	2.63E+8	1.19E+3	1.31E+3	1.11E+3	7.57E+3	1.19E+3	1.30E+3	1.11E+3	1.20E+3	1.14E+3	1.14E+3	1.16E+3	1.29E+3	1.25E+3
	Std	**2.15E+0**	3.79E+8	1.09E+2	1.74E+2	1.39E+0	8.31E+3	6.31E+1	8.05E+2	3.25E+0	1.39E+2	6.36E+1	1.81E+1	8.77E+1	1.04E+2	8.82E+1
F17	Mean.	3.59E+3	1.31E+10	**2.92E+3**	2.47E+4	6.76E+3	2.36E+4	1.87E+4	1.40E+4	2.94E+3	1.41E+4	1.46E+4	1.10E+4	1.32E+4	1.03E+5	1.65E+4
	Std	1.24E+3	9.99E+9	**3.51E+3**	1.87E+4	4.22E+3	1.36E+4	1.23E+4	9.83E+3	2.04E+3	1.14E+4	1.19E+4	8.33E+3	1.34E+4	8.68E+4	1.71E+4
F18	Mean.	1.62E+3	5.11E+9	1.83E+3	2.39E+4	2.22E+3	2.20E+4	6.18E+3	7.58E+3	**1.58E+3**	6.05E+3	3.33E+3	4.35E+3	1.24E+4	4.67E+3	9.14E+3
	Std	9.04E+1	5.04E+9	3.69E+2	1.95E+4	1.08E+3	2.13E+4	5.65E+3	6.97E+3	**4.06E+1**	4.40E+3	2.43E+3	3.82E+3	1.09E+4	3.13E+3	7.17E+3
F19	Mean.	1.72E+3	8.02E+5	1.75E+3	1.84E+3	**1.72E+3**	1.88E+3	1.87E+3	1.78E+3	1.74E+3	1.79E+3	1.79E+3	1.77E+3	1.77E+3	1.79E+3	1.82E+3
	Std	8.35E+0	1.81E+6	1.73E+1	7.55E+1	**7.44E+0**	9.48E+1	6.75E+1	4.42E+1	9.38E+0	5.75E+1	5.92E+1	3.12E+1	6.03E+1	1.98E+1	5.64E+1
F20	Mean.	**1.93E+3**	3.29E+10	2.09E+3	3.36E+4	2.47E+3	1.62E+4	3.17E+3	3.22E+4	1.97E+3	1.21E+4	3.45E+3	4.94E+3	1.48E+4	1.54E+4	1.80E+5
	Std	**3.16E+1**	3.46E+10	3.01E+2	4.10E+4	8.92E+2	1.66E+4	1.46E+3	7.14E+4	6.88E+1	2.07E+4	2.54E+3	3.72E+3	1.36E+4	9.30E+3	3.70E+5
F21	Mean.	2.30E+3	2.76E+3	2.30E+3	2.31E+3	2.29E+3	2.39E+3	2.30E+3	2.31E+3	2.30E+3	2.31E+3	2.31E+3	2.33E+3	2.33E+3	**2.28E+3**	2.34E+3
	Std	2.69E+1	3.12E+2	4.25E+1	7.02E+1	3.73E+1	4.41E+1	5.25E+1	3.45E+1	4.00E+1	5.77E+1	4.25E+1	5.48E+1	2.55E+1	**6.60E+1**	5.61E+1
F22	Mean.	**2.90E+3**	1.86E+4	2.92E+3	2.96E+3	2.93E+3	3.14E+3	2.92E+3	2.94E+3	2.91E+3	2.94E+3	2.92E+3	2.92E+3	2.94E+3	2.98E+3	2.96E+3
	Std	**1.46E-1**	1.60E+4	2.39E+1	2.92E+1	1.58E+1	1.31E+2	4.73E+1	1.65E+1	2.10E+1	2.59E+1	2.99E+1	2.35E+1	3.15E+1	1.66E+1	2.79E+1
F23	Mean.	**3.09E+3**	1.02E+4	3.10E+3	3.15E+3	3.09E+3	3.27E+3	3.10E+3	3.11E+3	3.09E+3	3.10E+3	3.10E+3	3.19E+3	3.11E+3	3.11E+3	3.14E+3
	Std	**6.55E-1**	6.87E+3	3.48E+0	3.77E+1	1.19E+0	5.80E+1	1.87E+1	2.02E+1	1.00E+0	3.73E+0	2.37E+1	4.51E+1	2.55E+1	3.83E+0	4.03E+1
F24	Mean.	**3.14E+3**	2.67E+7	3.18E+3	3.34E+3	3.20E+3	3.42E+3	3.29E+3	3.22E+3	3.16E+3	3.25E+3	3.24E+3	3.26E+3	3.25E+3	3.26E+3	3.45E+3
	Std	**6.00E+0**	4.87E+7	3.82E+1	9.61E+1	2.22E+1	1.18E+2	7.23E+1	4.45E+1	1.50E+1	6.36E+1	6.42E+1	6.69E+1	7.49E+1	5.67E+1	1.37E+2
**Dominant quantity**		**11**	0	1	0	3	0	0	0	1	0	0	0	0	1	2

Table A.3 Results and comparison of different algorithms on 19 benchmark functions with dim 30 (F1-F7, F13-F24)

Functions	Index	BAGWO	BAS	CSA	DA	DE	GA	GOA	GWO	IGWO	MFO	MVO	PSO	SA	SCA	WOA
F1	Mean.	1.40E-31	1.02E+5	1.59E+1	2.25E+3	4.39E-4	2.12E+4	4.01E+1	8.11E-28	2.48E-28	1.29E+3	1.21E+0	2.45E+0	4.17E-7	2.48E+1	**2.54E-75**
	Std	5.13E-32	1.03E+5	1.73E+1	1.03E+3	1.69E-4	5.65E+3	2.91E+1	7.90E-28	4.86E-28	4.26E+3	3.56E-1	1.09E+0	8.24E-7	4.89E+1	**1.35E-74**
F2	Mean.	1.02E+0	3.27E+7	2.03E+3	1.23E+4	3.31E+4	5.53E+4	3.59E+3	**4.71E-5**	1.76E-3	2.02E+4	1.97E+2	1.76E+2	7.79E+3	9.08E+3	3.80E+4
	Std	1.05E+0	2.99E+7	6.01E+2	8.49E+3	5.34E+3	1.36E+4	1.89E+3	**1.89E-4**	3.44E-3	1.35E+4	6.57E+1	5.49E+1	2.18E+3	6.02E+3	1.36E+4
F3	Mean.	**1.86E-31**	7.69E+4	1.94E+1	2.20E+3	4.02E-4	2.13E+4	3.27E+1	7.36E-1	1.62E-2	2.01E+3	1.27E+0	2.32E+0	7.14E-7	1.92E+1	4.70E-1
	Std	**9.13E-32**	8.22E+4	2.03E+1	1.11E+3	1.44E-4	5.82E+3	1.77E+1	3.92E-1	6.14E-2	4.85E+3	3.07E-1	1.11E+0	1.42E-6	2.66E+1	3.09E-1
F4	Mean.	**2.90E+3**	2.16E+11	1.97E+8	1.00E+10	1.05E+6	5.00E+10	8.15E+7	3.15E+9	4.00E+6	8.59E+9	2.09E+6	3.02E+6	1.01E+4	2.12E+10	5.65E+9
	Std	**2.65E+3**	1.25E+11	1.39E+8	3.63E+9	6.50E+5	1.95E+10	5.18E+7	2.11E+9	2.65E+6	6.70E+9	5.85E+5	1.53E+6	8.33E+3	3.36E+9	2.29E+9
F5	Mean.	-8.40E+3	2.22E+2	-7.33E+3	-5.22E+3	-1.01E+4	-2.15E+3	-7.22E+3	-5.69E+3	-8.14E+3	-8.66E+3	-7.55E+3	-6.25E+3	**-1.21E+4**	-3.80E+3	-9.96E+3
	Std	4.56E+2	6.41E+3	2.27E+3	7.17E+2	5.67E+2	5.23E+2	5.94E+2	9.76E+2	1.83E+3	9.53E+2	8.62E+2	1.43E+3	**2.44E+2**	2.70E+2	1.81E+3
F6	Mean.	2.15E-14	1.70E+1	2.00E+1	1.11E+1	5.69E-3	1.98E+1	5.40E+0	9.89E-14	6.02E-14	1.56E+1	1.76E+0	2.74E+0	4.92E-3	1.54E+1	**3.88E-15**
	Std	5.10E-15	4.42E+0	5.58E-11	1.85E+0	1.14E-3	4.29E-1	1.69E+0	1.96E-14	1.15E-14	6.10E+0	5.94E-1	4.59E-1	6.87E-3	7.55E+0	**1.98E-15**
F7	Mean.	**3.64E-31**	9.04E+8	9.62E+0	3.37E+1	1.38E-4	1.08E+2	3.01E+1	3.79E-2	3.91E-3	4.97E+1	5.99E+1	5.34E-2	3.46E-3	2.56E+0	2.71E-2
	Std	**3.30E-31**	1.68E+9	3.06E+0	1.64E+1	1.14E-4	3.63E+1	4.79E+1	1.75E-2	1.89E-2	3.66E+1	3.53E+1	5.62E-2	1.89E-2	2.69E+0	2.57E-2
F13	Mean.	**4.86E+2**	2.15E+5	5.96E+2	1.55E+3	5.19E+2	1.16E+4	5.42E+2	6.34E+2	5.09E+2	1.14E+3	4.97E+2	5.20E+2	5.04E+2	3.13E+3	1.50E+3
	Std	**8.42E+0**	2.57E+5	3.45E+1	5.65E+2	2.16E+1	7.38E+3	4.41E+1	8.87E+1	1.47E+1	7.50E+2	1.40E+1	3.22E+1	2.16E+1	1.21E+3	5.12E+2
F14	Mean.	6.03E+2	7.98E+2	6.36E+2	6.80E+2	6.00E+2	7.18E+2	6.57E+2	6.14E+2	6.02E+2	6.40E+2	6.35E+2	6.50E+2	**6.00E+2**	6.66E+2	6.78E+2
	Std	1.07E+0	8.06E+1	8.05E+0	9.69E+0	4.50E-2	1.71E+1	1.69E+1	5.98E+0	1.12E+0	1.06E+1	1.45E+1	6.89E+0	**1.75E-1**	7.14E+0	1.12E+1
F15	Mean.	**8.38E+2**	1.61E+3	9.13E+2	1.10E+3	9.94E+2	1.26E+3	9.83E+2	9.09E+2	8.77E+2	1.01E+3	9.41E+2	9.37E+2	9.43E+2	1.10E+3	1.08E+3
	Std	**8.68E+0**	2.85E+2	2.03E+1	5.18E+1	1.01E+1	5.51E+1	4.87E+1	4.46E+1	4.65E+1	3.88E+1	4.46E+1	2.77E+1	3.66E+1	2.06E+1	7.09E+1
F16	Mean.	**1.22E+3**	7.31E+10	1.53E+3	4.96E+3	1.82E+3	2.19E+4	1.56E+3	2.63E+3	1.24E+3	4.77E+3	1.34E+3	1.26E+3	5.04E+3	3.78E+3	1.00E+4
	Std	**2.87E+1**	1.28E+11	1.63E+2	2.41E+3	3.97E+2	1.05E+4	1.42E+2	1.22E+3	3.24E+1	3.47E+3	6.32E+1	3.44E+1	3.57E+3	8.47E+2	4.19E+3
F17	Mean.	4.05E+4	6.68E+10	8.84E+4	1.44E+8	4.34E+6	4.48E+9	1.96E+5	6.89E+6	1.66E+5	1.54E+8	1.72E+5	**2.88E+4**	4.04E+4	1.37E+9	1.20E+7
	Std	1.69E+4	4.12E+10	4.88E+4	2.14E+8	2.94E+6	3.65E+9	1.29E+5	2.29E+7	9.50E+4	5.25E+8	8.36E+4	**1.55E+4**	5.41E+4	1.08E+9	1.06E+7
F18	Mean.	2.35E+4	6.69E+10	3.92E+4	1.21E+6	8.81E+5	4.33E+8	8.34E+4	1.81E+6	4.52E+4	6.31E+4	7.15E+4	**1.19E+4**	2.12E+4	6.51E+7	1.10E+7
	Std	1.74E+4	5.69E+10	2.64E+4	2.91E+6	7.01E+5	7.18E+8	4.64E+4	6.48E+6	2.76E+4	5.38E+4	4.60E+4	**1.21E+4**	1.46E+4	6.01E+7	3.03E+7
F19	Mean.	**1.83E+3**	1.28E+8	2.08E+3	2.80E+3	2.23E+3	3.36E+3	2.35E+3	2.14E+3	1.95E+3	2.46E+3	2.16E+3	2.35E+3	2.50E+3	2.84E+3	2.75E+3
	Std	**4.90E+1**	2.68E+8	1.62E+2	2.44E+2	1.30E+2	4.25E+2	2.42E+2	1.80E+2	1.51E+2	2.55E+2	1.92E+2	2.34E+2	2.22E+2	1.89E+2	2.50E+2
F20	Mean.	1.24E+4	7.69E+10	1.48E+6	4.85E+7	7.18E+5	2.26E+8	5.81E+6	1.39E+6	2.93E+4	1.18E+6	2.00E+6	**8.45E+3**	2.50E+4	1.27E+8	1.88E+7
	Std	6.69E+3	7.44E+10	1.77E+6	8.58E+7	6.03E+5	2.78E+8	4.02E+6	1.50E+6	2.79E+4	2.27E+6	1.90E+6	**7.19E+3**	1.99E+4	9.97E+7	1.69E+7
F21	Mean.	**2.34E+3**	3.40E+3	2.43E+3	2.66E+3	2.49E+3	2.82E+3	2.48E+3	2.41E+3	2.38E+3	2.50E+3	2.42E+3	2.50E+3	2.44E+3	2.61E+3	2.64E+3
	Std	**1.21E+1**	2.22E+2	2.85E+1	7.98E+1	1.44E+1	9.43E+1	3.99E+1	2.31E+1	4.46E+1	4.53E+1	2.66E+1	3.90E+1	3.25E+1	2.58E+1	6.47E+1
F22	Mean.	**2.89E+3**	5.74E+4	3.02E+3	3.53E+3	2.90E+3	6.18E+3	2.97E+3	3.01E+3	2.90E+3	3.16E+3	2.90E+3	2.92E+3	2.90E+3	3.58E+3	3.22E+3
	Std	**5.25E-1**	5.99E+4	4.58E+1	2.63E+2	6.62E+0	1.52E+3	3.86E+1	4.51E+1	1.37E+1	2.28E+2	1.75E+1	1.96E+1	2.37E+1	2.06E+2	6.77E+1
F23	Mean.	**3.21E+3**	9.97E+3	3.30E+3	3.54E+3	3.23E+3	4.40E+3	3.28E+3	3.27E+3	3.21E+3	3.25E+3	3.23E+3	3.44E+3	3.24E+3	3.55E+3	3.51E+3
	Std	**5.89E+0**	6.45E+2	3.25E+1	1.80E+2	3.90E+0	3.86E+2	3.15E+1	2.30E+1	6.90E+0	2.12E+1	1.36E+1	1.47E+2	2.10E+1	7.33E+1	1.56E+2
F24	Mean.	**3.57E+3**	1.26E+8	4.22E+3	5.43E+3	4.15E+3	5.94E+3	4.35E+3	3.94E+3	3.62E+3	4.06E+3	4.09E+3	4.26E+3	3.99E+3	5.33E+3	5.40E+3
	Std	**6.68E+1**	2.37E+8	2.75E+2	6.84E+2	1.45E+2	7.17E+2	3.82E+2	2.35E+2	2.13E+2	2.07E+2	3.09E+2	2.96E+2	2.49E+2	3.50E+2	6.01E+2
**Dominant quantity**		**11**	0	0	0	0	0	0	1	0	0	0	3	2	0	2

Table A.4 Results and comparison of different algorithms on 19 benchmark functions with dim 50 (F1-F7, F13-F24)

Functions	Index	BAGWO	BAS	CSA	DA	DE	GA	GOA	GWO	IGWO	MFO	MVO	PSO	SA	SCA	WOA
F1	Mean.	2.88E-26	1.57E+5	4.67E+2	8.29E+3	1.63E+0	7.55E+4	1.43E+3	8.45E-20	2.27E-20	7.45E+3	9.76E+0	2.44E+1	2.15E-3	9.77E+2	**1.09E-73**
	Std	2.26E-26	1.58E+5	1.51E+2	4.06E+3	4.10E-1	1.60E+4	4.40E+2	6.66E-20	1.84E-20	6.91E+3	2.32E+0	7.92E+0	6.51E-3	2.07E+3	**3.91E-73**
F2	Mean.	2.41E+2	9.74E+7	1.01E+4	5.09E+4	1.08E+5	1.65E+5	1.53E+4	**4.93E-1**	1.51E+1	6.42E+4	6.00E+3	2.23E+3	2.99E+4	5.18E+4	1.95E+5
	Std	8.42E+1	1.03E+8	3.23E+3	1.89E+4	9.92E+3	4.44E+4	7.17E+3	**7.59E-1**	2.06E+1	2.08E+4	1.34E+3	4.90E+2	5.87E+3	1.71E+4	4.38E+4
F3	Mean.	**4.07E-26**	1.85E+5	3.81E+2	8.63E+3	1.65E+0	7.93E+4	1.33E+3	2.79E+0	1.21E+0	9.42E+3	9.49E+0	2.23E+1	7.34E-4	6.26E+2	1.38E+0
	Std	**3.30E-26**	1.57E+5	1.25E+2	3.61E+3	4.94E-1	1.56E+4	4.23E+2	7.50E-1	4.11E-1	7.75E+3	1.83E+0	5.77E+0	7.44E-4	8.01E+2	3.99E-1
F4	Mean.	**2.53E+3**	4.22E+11	5.01E+9	4.90E+10	6.41E+7	1.61E+11	5.39E+9	1.09E+10	9.88E+8	3.90E+10	1.71E+7	3.16E+8	9.33E+4	6.63E+10	2.17E+10
	Std	**2.44E+3**	2.06E+11	1.78E+9	1.50E+10	3.99E+7	3.41E+10	2.07E+9	3.85E+9	1.13E+9	1.75E+10	4.12E+6	9.53E+8	1.94E+5	8.50E+9	4.43E+9
F5	Mean.	-1.28E+4	2.36E+3	-1.15E+4	-7.10E+3	-1.19E+4	-2.75E+3	-1.14E+4	-9.26E+3	-1.04E+4	-1.32E+4	-1.26E+4	-9.50E+3	**-1.98E+4**	-4.94E+3	-1.66E+4
	Std	7.70E+2	1.07E+4	3.87E+3	6.34E+2	6.09E+2	5.05E+2	1.01E+3	8.34E+2	2.65E+3	1.23E+3	8.29E+2	2.21E+3	**3.61E+2**	3.71E+2	2.97E+3
F6	Mean.	8.04E-14	1.82E+1	2.00E+1	1.24E+1	5.33E-1	2.04E+1	1.02E+1	3.08E-11	2.85E-11	1.94E+1	2.89E+0	4.21E+0	7.53E-1	1.65E+1	**4.00E-15**
	Std	1.10E-14	4.48E+0	3.18E-10	2.42E+0	1.30E-1	2.30E-1	1.44E+0	1.74E-11	1.97E-11	1.03E+0	4.62E-1	2.74E-1	5.88E-1	7.26E+0	**2.09E-15**
F7	Mean.	1.77E+0	2.28E+9	1.31E+1	4.57E+1	2.23E+0	2.67E+2	3.13E+1	1.32E-1	2.16E-2	1.01E+2	1.18E+2	2.03E+0	**1.61E-2**	2.08E+1	3.78E-2
	Std	9.45E-1	2.77E+9	2.59E+0	3.37E+1	8.21E-1	5.28E+1	1.14E+1	1.00E-1	1.51E-2	3.46E+1	3.64E+1	1.79E+0	**3.94E-2**	2.15E+1	3.68E-2
F13	Mean.	**5.20E+2**	3.47E+5	1.48E+3	9.26E+3	7.35E+2	4.53E+4	1.12E+3	1.70E+3	7.10E+2	4.22E+3	6.10E+2	6.18E+2	6.28E+2	1.43E+4	4.33E+3
	Std	**1.88E+1**	3.93E+5	2.97E+2	3.68E+3	3.55E+1	1.43E+4	2.32E+2	8.18E+2	1.24E+2	2.79E+3	4.86E+1	7.37E+1	6.39E+1	2.25E+3	1.31E+3
F14	Mean.	6.13E+2	7.96E+2	6.53E+2	6.98E+2	6.03E+2	7.41E+2	6.73E+2	6.28E+2	6.07E+2	6.64E+2	6.55E+2	6.60E+2	**6.01E+2**	6.84E+2	6.95E+2
	Std	2.51E+0	6.79E+1	5.57E+0	1.15E+1	3.79E-1	1.36E+1	1.23E+1	5.65E+0	2.12E+0	9.46E+0	1.53E+1	5.93E+0	**3.80E-1**	6.98E+0	1.05E+1
F15	Mean.	**8.84E+2**	2.59E+3	1.11E+3	1.48E+3	1.23E+3	1.79E+3	1.23E+3	1.06E+3	1.01E+3	1.30E+3	1.08E+3	1.14E+3	1.08E+3	1.45E+3	1.43E+3
	Std	**1.75E+1**	1.04E+3	5.41E+1	5.23E+1	1.98E+1	1.26E+2	6.16E+1	7.59E+1	9.17E+1	6.75E+1	6.72E+1	4.30E+1	5.58E+1	4.52E+1	8.09E+1
F16	Mean.	1.50E+3	1.22E+7	6.55E+3	2.22E+4	7.38E+3	6.93E+4	4.47E+3	7.57E+3	1.73E+3	1.61E+4	1.67E+3	**1.47E+3**	1.47E+4	1.33E+4	8.43E+3
	Std	7.13E+1	2.16E+7	2.53E+3	7.04E+3	2.20E+3	2.11E+4	1.65E+3	2.86E+3	2.19E+2	1.33E+4	1.20E+2	**1.54E+2**	1.23E+4	3.43E+3	2.26E+3
F17	Mean.	4.64E+4	2.06E+11	1.33E+6	2.44E+9	1.29E+7	3.36E+10	8.41E+5	5.47E+8	1.39E+6	1.89E+9	3.67E+5	2.07E+6	**2.19E+4**	7.43E+9	4.78E+8
	Std	1.70E+4	8.24E+10	5.69E+6	2.25E+9	1.32E+7	1.61E+10	2.63E+6	1.83E+9	1.01E+6	2.61E+9	1.85E+5	5.95E+6	**1.46E+4**	2.74E+9	2.19E+8
F18	Mean.	2.20E+4	1.02E+11	4.77E+4	1.30E+8	1.68E+6	7.73E+9	8.24E+4	2.43E+7	1.34E+5	2.83E+7	1.09E+5	2.24E+4	**1.94E+4**	1.33E+9	6.40E+7
	Std	7.06E+3	6.88E+10	2.68E+4	3.01E+8	2.12E+6	5.32E+9	4.52E+4	3.17E+7	1.52E+5	7.91E+7	4.45E+4	1.23E+4	**1.63E+4**	6.45E+8	6.53E+7
F19	Mean.	**2.55E+3**	5.35E+8	3.41E+3	4.46E+3	3.61E+3	2.46E+4	3.54E+3	3.12E+3	2.92E+3	3.90E+3	3.29E+3	3.15E+3	3.49E+3	5.06E+3	4.45E+3
	Std	**1.91E+2**	1.28E+9	3.60E+2	4.66E+2	2.12E+2	2.29E+4	3.77E+2	3.94E+2	5.33E+2	4.36E+2	3.89E+2	3.46E+2	3.53E+2	3.09E+2	5.73E+2
F20	Mean.	6.86E+4	4.30E+10	3.02E+6	7.22E+7	7.17E+5	3.40E+9	1.13E+7	1.27E+7	1.77E+5	8.66E+7	6.16E+6	2.86E+4	**1.89E+4**	6.99E+8	2.99E+7
	Std	3.22E+4	3.32E+10	5.03E+6	6.75E+7	6.56E+5	1.80E+9	7.56E+6	3.08E+7	1.74E+5	2.53E+8	3.95E+6	1.71E+4	**1.79E+4**	3.43E+8	3.05E+7
F21	Mean.	**2.38E+3**	4.64E+3	2.64E+3	3.04E+3	2.74E+3	3.42E+3	2.77E+3	2.59E+3	2.49E+3	2.78E+3	2.58E+3	2.73E+3	2.58E+3	2.95E+3	3.06E+3
	Std	**1.36E+1**	7.92E+2	5.41E+1	8.73E+1	2.03E+1	1.14E+2	9.19E+1	8.63E+1	7.67E+1	1.02E+2	5.12E+1	7.38E+1	4.12E+1	4.59E+1	1.08E+2
F22	Mean.	**3.02E+3**	2.36E+5	3.91E+3	8.47E+3	3.17E+3	2.71E+4	3.59E+3	3.87E+3	3.22E+3	5.47E+3	3.07E+3	3.10E+3	3.07E+3	9.50E+3	5.36E+3
	Std	**1.34E+1**	2.75E+5	3.40E+2	1.56E+3	2.51E+1	6.84E+3	3.53E+2	3.58E+2	9.64E+1	2.23E+3	3.17E+1	3.52E+1	3.52E+1	1.32E+3	5.51E+2
F23	Mean.	**3.32E+3**	3.02E+4	3.92E+3	4.57E+3	3.58E+3	6.84E+3	3.71E+3	3.75E+3	3.36E+3	3.63E+3	3.47E+3	4.45E+3	3.52E+3	4.97E+3	5.08E+3
	Std	**2.06E+1**	1.70E+4	1.69E+2	3.79E+2	5.07E+1	1.12E+3	1.66E+2	1.08E+2	7.35E+1	1.26E+2	9.18E+1	5.16E+2	9.84E+1	2.75E+2	6.39E+2
F24	Mean.	**4.11E+3**	6.94E+8	6.07E+3	9.50E+3	5.57E+3	4.14E+4	5.91E+3	4.91E+3	4.16E+3	5.63E+3	5.27E+3	5.49E+3	4.58E+3	9.40E+3	9.98E+3
	Std	**1.33E+2**	1.22E+9	5.78E+2	2.75E+3	2.23E+2	8.11E+4	6.17E+2	3.17E+2	3.62E+2	5.59E+2	4.78E+2	5.27E+2	3.17E+2	1.65E+3	2.48E+3
**Dominant quantity**		**9**	0	0	0	0	0	0	1	0	0	0	1	6	0	2

Table A.5 Results and comparison of different algorithms on 19 benchmark functions with dim 100 (F1-F7, F13-F24)

Functions	Index	BAGWO	BAS	CSA	DA	DE	GA	GOA	GWO	IGWO	MFO	MVO	PSO	SA	SCA	WOA
F1	Mean.	4.44E-16	3.53E+5	4.86E+3	2.19E+4	1.80E+3	2.18E+5	1.35E+4	1.45E-12	2.31E-12	6.55E+4	1.67E+2	1.92E+2	2.34E+0	1.36E+4	**6.29E-71**
	Std	3.39E-16	2.93E+5	1.15E+3	1.24E+4	3.13E+2	2.85E+4	2.35E+3	1.21E-12	1.89E-12	1.67E+4	3.46E+1	3.22E+1	1.72E+0	7.23E+3	**2.17E-70**
F2	Mean.	5.30E+3	1.61E+9	4.23E+4	2.25E+5	4.32E+5	6.80E+5	7.70E+4	**8.64E+2**	6.51E+3	2.53E+5	6.57E+4	2.06E+4	1.46E+5	2.46E+5	1.02E+6
	Std	8.81E+2	1.16E+9	1.05E+4	8.75E+4	4.54E+4	1.63E+5	2.94E+4	**1.31E+3**	3.49E+3	6.65E+4	7.27E+3	4.68E+3	2.00E+4	5.31E+4	3.44E+5
F3	Mean.	**3.77E-16**	2.80E+5	5.28E+3	1.95E+4	1.80E+3	2.28E+5	1.26E+4	9.88E+0	7.78E+0	6.72E+4	1.57E+2	1.96E+2	1.80E+0	1.28E+4	4.20E+0
	Std	**2.41E-16**	2.49E+5	1.57E+3	1.03E+4	2.81E+2	2.00E+4	2.18E+3	9.23E-1	1.20E+0	1.49E+4	2.71E+1	3.56E+1	9.35E-1	7.18E+3	1.25E+0
F4	Mean.	**5.94E+3**	8.40E+11	5.60E+10	2.06E+11	4.76E+9	5.39E+11	9.07E+10	5.44E+10	2.43E+10	1.52E+11	3.25E+8	2.16E+9	1.11E+7	2.11E+11	1.11E+11
	Std	**5.04E+3**	3.96E+11	9.05E+9	2.64E+10	1.28E+9	5.63E+10	1.71E+10	1.08E+10	7.35E+9	5.11E+10	6.68E+7	2.77E+9	8.19E+6	1.45E+10	1.20E+10
F5	Mean.	-2.45E+4	2.98E+3	-2.41E+4	-1.02E+4	-1.63E+4	-3.79E+3	-1.85E+4	-1.58E+4	-1.50E+4	-2.28E+4	-2.30E+4	-1.84E+4	**-3.81E+4**	-6.89E+3	-3.46E+4
	Std	1.51E+3	1.88E+4	9.18E+3	1.14E+3	5.95E+2	8.89E+2	1.43E+3	3.13E+3	6.00E+3	2.11E+3	1.48E+3	5.58E+3	**5.58E+2**	6.04E+2	6.32E+3
F6	Mean.	6.74E-3	1.91E+1	2.00E+1	1.25E+1	7.42E+0	2.08E+1	1.33E+1	1.35E-7	1.52E-7	1.98E+1	9.01E+0	6.75E+0	9.58E+0	1.96E+1	**3.64E-15**
	Std	3.69E-2	3.64E+0	6.07E-10	2.59E+0	3.31E-1	1.07E-1	7.93E-1	6.02E-8	5.71E-8	1.86E-1	6.86E+0	3.86E-1	3.69E+0	3.23E+0	**2.53E-15**
F7	Mean.	7.16E+0	3.01E+9	2.01E+1	5.13E+1	7.64E+1	5.45E+2	3.75E+1	2.42E-1	1.38E-1	1.79E+2	2.08E+2	1.61E+1	1.20E-1	6.11E+1	**4.34E-2**
	Std	1.99E+0	6.07E+9	4.67E+0	3.34E+1	9.20E+0	7.65E+1	7.07E+0	5.06E-2	4.84E-2	2.52E+1	3.59E+1	5.58E+0	8.94E-2	2.69E+1	**2.51E-2**
F13	Mean.	**7.06E+2**	7.51E+5	8.40E+3	5.10E+4	2.10E+3	2.02E+5	1.16E+4	6.28E+3	2.74E+3	2.90E+4	8.78E+2	9.89E+2	7.58E+2	5.16E+4	2.12E+4
	Std	**2.77E+1**	5.45E+5	2.20E+3	1.40E+4	3.08E+2	5.60E+4	2.84E+3	2.12E+3	6.84E+2	1.03E+4	7.60E+1	2.76E+2	6.48E+1	7.42E+3	4.15E+3
F14	Mean.	6.33E+2	7.94E+2	6.74E+2	7.14E+2	6.22E+2	7.57E+2	6.91E+2	6.45E+2	6.24E+2	6.88E+2	6.80E+2	6.73E+2	**6.04E+2**	7.05E+2	7.09E+2
	Std	2.86E+0	5.68E+1	5.03E+0	9.30E+0	1.49E+0	1.05E+1	7.94E+0	5.36E+0	4.47E+0	7.41E+0	1.10E+1	4.36E+0	**1.36E+0**	5.06E+0	1.00E+1
F15	Mean.	**1.05E+3**	4.42E+3	1.88E+3	2.52E+3	1.94E+3	3.29E+3	2.16E+3	1.55E+3	1.43E+3	2.28E+3	1.63E+3	1.81E+3	1.55E+3	2.42E+3	2.41E+3
	Std	**3.71E+1**	1.36E+3	7.09E+1	1.22E+2	3.33E+1	1.60E+2	1.12E+2	6.39E+1	1.37E+2	1.79E+2	1.24E+2	7.37E+1	1.14E+2	6.16E+1	1.40E+2
F16	Mean.	3.60E+4	3.41E+14	1.22E+5	3.41E+5	2.57E+5	5.25E+5	1.01E+5	8.83E+4	3.17E+4	2.26E+5	**1.66E+4**	3.83E+4	1.47E+5	1.80E+5	2.81E+5
	Std	7.59E+3	5.32E+14	2.35E+4	1.01E+5	3.74E+4	1.43E+5	3.06E+4	1.87E+4	8.30E+3	7.29E+4	**5.00E+3**	1.17E+4	4.84E+4	3.30E+4	8.66E+4
F17	Mean.	**3.53E+4**	1.81E+11	7.03E+7	1.24E+10	4.95E+5	6.96E+10	2.90E+7	1.72E+9	1.33E+7	4.62E+9	1.28E+6	6.85E+7	1.96E+6	1.73E+10	2.98E+9
	Std	**9.19E+3**	9.60E+10	5.64E+7	5.02E+9	7.69E+5	1.60E+10	1.92E+7	1.42E+9	1.26E+7	3.28E+9	6.41E+5	1.65E+8	2.94E+6	2.66E+9	1.69E+9
F18	Mean.	**2.58E+4**	1.18E+11	3.55E+5	3.93E+9	4.59E+6	3.06E+10	8.92E+5	3.17E+8	7.52E+5	1.71E+9	4.21E+5	2.21E+5	2.53E+5	5.54E+9	4.67E+8
	Std	**7.31E+3**	7.19E+10	4.01E+5	2.27E+9	6.99E+6	8.38E+9	1.46E+6	4.29E+8	5.80E+5	1.48E+9	7.65E+5	1.49E+5	7.84E+5	1.20E+9	2.63E+8
F19	Mean.	**4.17E+3**	1.23E+10	6.42E+3	2.75E+5	8.02E+3	6.27E+6	6.29E+3	5.49E+3	5.41E+3	1.38E+4	5.73E+3	5.33E+3	5.70E+3	4.46E+4	3.30E+4
	Std	**3.75E+2**	2.46E+10	6.69E+2	6.66E+5	3.69E+2	1.26E+7	7.24E+2	5.21E+2	1.16E+3	1.57E+4	5.93E+2	4.73E+2	5.18E+2	3.53E+4	5.73E+4
F20	Mean.	3.82E+5	1.11E+11	2.78E+7	3.79E+9	6.86E+6	3.18E+10	3.14E+7	3.24E+8	3.54E+6	1.80E+9	2.17E+7	6.54E+6	**3.02E+4**	5.82E+9	4.33E+8
	Std	2.42E+5	6.54E+10	2.99E+7	2.55E+9	7.53E+6	1.08E+10	1.90E+7	4.39E+8	2.05E+6	1.79E+9	1.22E+7	2.03E+7	**5.47E+4**	1.68E+9	1.50E+8
F21	Mean.	**2.59E+3**	8.12E+3	3.43E+3	4.56E+3	3.48E+3	5.30E+3	3.81E+3	3.08E+3	2.96E+3	3.79E+3	3.21E+3	3.74E+3	3.08E+3	4.19E+3	4.49E+3
	Std	**4.62E+1**	1.95E+3	1.08E+2	2.28E+2	3.36E+1	2.43E+2	1.62E+2	6.94E+1	1.86E+2	1.53E+2	1.22E+2	1.51E+2	9.69E+1	1.03E+2	2.21E+2
F22	Mean.	**3.41E+3**	5.06E+5	8.21E+3	2.13E+4	6.10E+3	1.02E+5	1.00E+4	7.24E+3	5.01E+3	1.72E+4	3.60E+3	3.56E+3	3.49E+3	2.29E+4	1.08E+4
	Std	**4.15E+1**	5.72E+5	1.02E+3	5.12E+3	4.50E+2	1.75E+4	1.43E+3	9.80E+2	4.97E+2	6.25E+3	5.32E+1	7.67E+1	7.50E+1	2.44E+3	9.90E+2
F23	Mean.	3.59E+3	2.79E+4	4.89E+3	7.59E+3	4.36E+3	1.42E+4	4.24E+3	4.29E+3	3.70E+3	4.11E+3	3.74E+3	4.20E+3	**3.57E+3**	8.56E+3	6.60E+3
	Std	4.09E+1	4.35E+3	4.37E+2	1.03E+3	1.73E+2	1.86E+3	2.21E+2	1.72E+2	7.42E+1	2.42E+2	1.51E+2	3.66E+2	**6.73E+1**	4.98E+2	9.46E+2
F24	Mean.	7.21E+3	1.29E+9	1.30E+4	2.75E+4	1.06E+4	1.87E+6	1.22E+4	9.50E+3	7.31E+3	1.76E+4	9.11E+3	9.10E+3	**7.01E+3**	3.51E+4	2.08E+4
	Std	4.74E+2	2.44E+9	1.39E+3	1.35E+4	3.02E+2	2.42E+6	1.80E+3	1.74E+3	6.15E+2	2.17E+4	7.30E+2	8.11E+2	**6.04E+2**	1.33E+4	4.23E+3
**Dominant quantity**		**9**	0	0	0	0	0	0	1	0	0	1	0	5	0	3

Table A.6 Results and comparison of different algorithms on eight engineering problems

Algorithms	Index	Engineering problems								Dominant quantity
		TCSD	PVD	WBD	SRD	TTD	CBD	GTD	SPD	
BAGWO	Mean.	1.2715E-2	6.14E+3	**1.6937E+0**	3.00E+3	2.64E+2	**1.34E+0**	**9.49E-12**	**1.61E+1**	**4**
	Std	1.3117E-5	1.14E+2	**9.6914E-4**	1.29E+0	1.79E-7	**2.38E-7**	**9.77E-12**	**3.80E-7**	
BAS	Mean.	7.17E+4	8.88E+17	3.82E+4	4.15E+3	2.68E+2	1.45E+1	3.10E-1	2.83E+11	0
	Std	1.48E+5	4.82E+18	1.14E+5	6.10E+2	4.39E+0	8.77E+0	1.01E-1	3.72E+11	
CSA	Mean.	1.2696E-2	6.08E+3	1.70E+0	2.99E+3	**2.64E+2**	1.34E+0	7.97E-10	1.62E+1	**1**
	Std	2.5061E-5	2.12E+2	1.93E-2	1.83E+0	**1.14E-13**	5.88E-5	7.50E-10	2.02E-1	
DA	Mean.	1.68E-2	8.82E+3	1.81E+0	3.02E+3	2.64E+2	1.37E+0	4.06E-8	1.36E+2	0
	Std	1.51E-2	1.11E+4	1.40E-1	3.20E+1	1.17E-2	2.62E-2	1.62E-7	2.34E+2	
DE	Mean.	1.29E-2	6.20E+3	1.88E+0	**2.99E+3**	2.64E+2	1.34E+0	8.51E-10	1.66E+1	**1**
	Std	2.26E-4	2.53E+2	1.16E-1	**1.19E-13**	8.63E-5	1.01E-4	7.85E-10	2.84E-1	
GA	Mean.	9.98E+5	1.68E+20	5.35E+14	1.00E+30	1.00E+30	1.00E+30	7.32E-1	1.25E+12	0
	Std	1.25E+3	0.00E+0	0.00E+0	1.43E+14	1.43E+14	1.43E+14	4.52E-16	0.00E+0	
GOA	Mean.	1.81E-2	7.17E+3	1.35E+4	3.06E+3	2.64E+2	1.34E+0	2.67E-9	1.66E+1	0
	Std	4.55E-3	2.54E+3	5.13E+4	3.40E+1	2.30E-1	1.59E-3	4.45E-9	3.42E-1	
GWO	Mean.	1.28E-2	6.01E+3	1.70E+0	3.01E+3	2.64E+2	1.34E+0	6.17E-10	1.73E+1	0
	Std	1.26E-4	2.32E+2	2.51E-3	4.06E+0	4.04E-3	5.93E-5	6.93E-10	3.99E-1	
IGWO	Mean.	**1.2694E-2**	**5.89E+3**	1.6940E+0	2.99E+3	2.64E+2	1.34E+0	8.88E-11	1.63E+1	**2**
	Std	**1.3255E-5**	**2.66E+0**	5.2110E-4	1.18E-4	2.27E-4	4.01E-5	2.36E-10	1.14E-1	
MFO	Mean.	1.32E-2	6.51E+3	1.74E+0	3.00E+3	2.64E+2	1.34E+0	1.12E-8	1.72E+1	0
	Std	7.22E-4	6.15E+2	1.05E-1	7.67E+0	1.53E-1	1.11E-3	1.38E-8	5.07E-1	
MVO	Mean.	1.74E-2	7.14E+3	1.72E+0	3.01E+3	2.64E+2	1.34E+0	1.60E-9	1.67E+1	0
	Std	1.53E-3	5.36E+2	1.99E-2	1.13E+1	1.63E-3	8.24E-4	4.89E-9	3.52E-1	
PSO	Mean.	1.35E-2	6.19E+3	1.70E+0	3.08E+3	2.64E+2	1.34E+0	3.43E-8	5.22E+1	0
	Std	1.31E-3	3.39E+2	1.58E-2	7.69E+1	1.51E-5	2.31E-5	1.63E-7	1.55E+2	
SA	Mean.	1.39E-2	6.53E+3	1.52E+4	2.99E+3	2.67E+2	1.40E+0	2.39E-7	1.69E+1	0
	Std	1.66E-3	6.21E+2	4.14E+4	1.67E-6	3.10E+0	3.67E-2	5.07E-7	3.53E-1	
SCA	Mean.	1.33E-2	7.43E+3	1.89E+0	3.13E+3	2.65E+2	1.40E+0	4.52E-9	2.12E+2	0
	Std	5.53E-4	7.12E+2	6.40E-2	3.70E+1	3.43E+0	2.61E-2	6.50E-9	1.72E+2	
WOA	Mean.	1.40E-2	1.12E+4	2.66E+0	3.07E+3	2.65E+2	1.55E+0	4.01E-9	3.29E+1	0
	Std	1.16E-3	9.09E+3	7.15E-1	1.30E+2	1.13E+0	1.70E-1	6.88E-9	8.51E+1	
